# Enhanced rare-earth separation with a metal-sensitive lanmodulin dimer

**DOI:** 10.1038/s41586-023-05945-5

**Published:** 2023-05-31

**Authors:** Joseph A. Mattocks, Jonathan J. Jung, Chi-Yun Lin, Ziye Dong, Neela H. Yennawar, Emily R. Featherston, Christina S. Kang-Yun, Timothy A. Hamilton, Dan M. Park, Amie K. Boal, Joseph A. Cotruvo

**Affiliations:** 1 Department of Chemistry, The Pennsylvania State University, University Park, PA USA; 2 Critical Materials Institute, Physical and Life Sciences Directorate, Lawrence Livermore National Laboratory, Livermore, CA USA; 3 The Huck Institutes of the Life Sciences, The Pennsylvania State University, University Park, PA USA; 4 Department of Biochemistry and Molecular Biology, The Pennsylvania State University, University Park, PA USA

**Keywords:** Metalloproteins, X-ray crystallography, Biomaterials - proteins, Supramolecular chemistry, Sustainability

## Abstract

Technologically critical rare-earth elements are notoriously difficult to separate, owing to their subtle differences in ionic radius and coordination number^1–3^. The natural lanthanide-binding protein lanmodulin (LanM)^4,5^ is a sustainable alternative to conventional solvent-extraction-based separation^6^. Here we characterize a new LanM, from *Hansschlegelia quercus* (*Hans*-LanM), with an oligomeric state sensitive to rare-earth ionic radius, the lanthanum(III)-induced dimer being >100-fold tighter than the dysprosium(III)-induced dimer. X-ray crystal structures illustrate how picometre-scale differences in radius between lanthanum(III) and dysprosium(III) are propagated to *Hans*-LanM’s quaternary structure through a carboxylate shift that rearranges a second-sphere hydrogen-bonding network. Comparison to the prototypal LanM from *Methylorubrum extorquens* reveals distinct metal coordination strategies, rationalizing *Hans*-LanM’s greater selectivity within the rare-earth elements. Finally, structure-guided mutagenesis of a key residue at the *Hans-*LanM dimer interface modulates dimerization in solution and enables single-stage, column-based separation of a neodymium(III)/dysprosium(III) mixture to >98% individual element purities. This work showcases the natural diversity of selective lanthanide recognition motifs, and it reveals rare-earth-sensitive dimerization as a biological principle by which to tune the performance of biomolecule-based separation processes.

## Abstract (web summary)

A study biochemically and structurally characterizes a lanmodulin from *Hansschlegelia quercus* with an oligomeric state sensitive to rare-earth ionic radius.

## Main

The irreplaceable roles of rare-earth (RE) elements in ubiquitous modern technologies ranging from permanent magnets to light-emitting diodes and phosphors have renewed interest in one of the grand challenges of separation science—efficient separation of lanthanides^1^. The separation of these 15 elements is complicated by the similar physicochemical properties of their predominating +III ions, with ionic radii decreasing only 0.19 Å between La^III^ and Lu^III^ (ref. ^7^), which also leads to these metals co-occurring in RE-bearing minerals. Conventional hydrometallurgical liquid–liquid extraction methods for RE production utilize organic solvents such as kerosene and toxic phosphonate extractants and require dozens or even hundreds of stages to achieve high-purity individual RE oxides^3,8^. The inefficiency and large environmental impact of RE separations^9^ have stimulated research efforts into alternative ligands with larger separation factors between adjacent REs^10–14^, and greener process designs to achieve RE separation in fewer stages^15^ and using all-aqueous chemistry^6,16–20^.

The discovery of the founding member of the LanM family of lanthanide-binding proteins demonstrated that nature has evolved macromolecules surpassing the selectivity of synthetic f-element chelators^4^. The prototypal LanM, from *M. extorquens* AM1 (*Mex*-LanM), is a small (12-kDa), monomeric protein that undergoes a selective conformational response to picomolar concentrations of lanthanides^4,18^ and actinides^21–24^, has facilitated understanding of lanthanide uptake in methylotrophs^25^, and has served as a technology platform for f-element detection^26^, recovery^18,27^ and separation^6^. Unusually among RE chelators, *Mex*-LanM favours the larger and more abundant light REs (LREs), especially La^III^–Sm^III^, over heavy REs (HREs)^4^. Our recent demonstration that even single substitutions to the metal-binding motifs of *Mex*-LanM can improve actinide/lanthanide separations^23^ spurred us to investigate whether orthologues of *Mex*-LanM might possess distinct, and potentially useful, metal selectivity trends.

Herein, we report that the LanM from *Hansschlegelia quercus* (*Hans*-LanM), a methylotrophic bacterium isolated from English oak buds^28^, exhibits enhanced RE separation capacity relative to *Mex-*LanM. Whereas *Mex-*LanM is always monomeric, *Hans*-LanM exists in a monomer/dimer equilibrium, the position of which depends on the specific RE bound. Three X-ray crystal structures of LanMs and structure-guided mutagenesis explain *Hans*-LanM’s RE-dependent oligomeric state and its greater separation capacity than that of *Mex*-LanM. Finally, we leverage these findings to achieve single-stage *Hans*-LanM-based separation of the critical neodymium/dysprosium pair. These results illustrate how intermolecular interactions—common in proteins but rare in small molecules—may be exploited to improve RE separations.

## *Hans*-LanM’s distinct selectivity profile

**Fig. 1:**
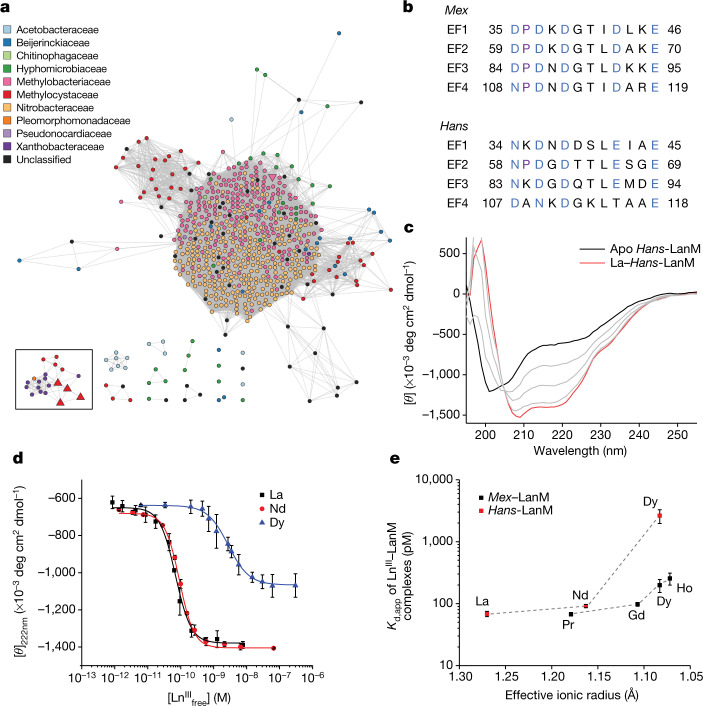
*Hans*-LanM diverges from *Mex*-LanM in sequence and RE versus RE selectivity. **a**, Sequence similarity network of core LanM sequences indicates that *Hans*-LanM forms a distinct cluster. The sequence similarity network includes 696 LanM sequences connected with 48,647 edges, thresholded at a BLAST *E* value of 1 × 10^−5^ and 65% sequence identity. The black box encloses nodes clustered with *Hans*-LanM. The LanM sequence associated with *Mex* (downtriangle) and four within *Hansschlegelia* (uptriangle) are enlarged compared to other nodes (circles). Colours of the nodes represent the family from which the sequences originate. **b**, Comparison of the sequences of the four EF hands of *Mex*- and *Hans*-LanMs. Residues canonically involved in metal binding in EF hands are in blue; Pro residues are in purple. **c**, Circular dichroism spectra from a representative titration of *Hans*-LanM with La^III^, showing the metal-associated conformational response increasing helicity; apoprotein is bold black, La^III^-saturated protein is bold red. **d**, Circular dichroism titration of *Hans*-LanM with La^III^, Nd^III^ and Dy^III^ (pH 5.0). Each point represents the mean ± s.d. from three independent experiments. **e**, Comparison of *K*_d,app_ values (pH 5.0) for *Mex*-LanM (black^18^) and *Hans-*LanM (red), plotted versus ionic radius^7^. Mean ± s.e.m. from three independent experiments. Source Data

**Extended Data Fig. 1:**
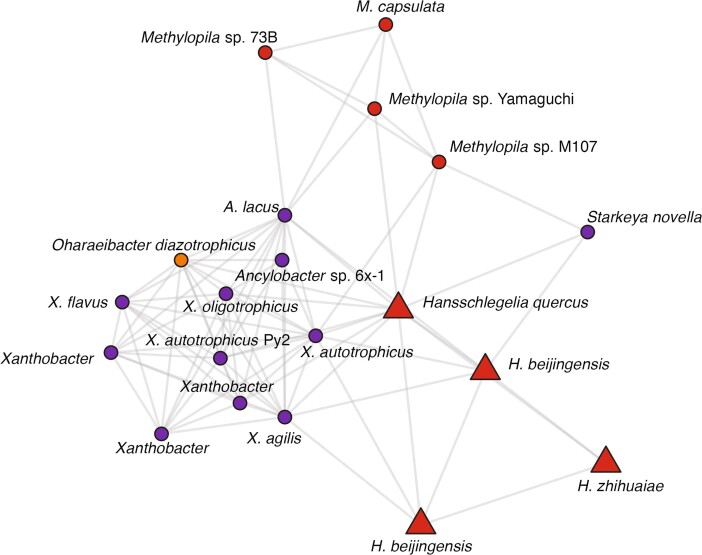
Expanded view of the inset from Fig. 1a (*Hans* cluster) including 20 sequences and 190 edges. The *Hans* cluster includes LanMs from bacteria from genera *Hansschlegelia*, *Ancylobacter*, *Methylopila*, *Oharaeibacter*, *Starkeya*, and *Xanthobacter*. Although these genera are restricted to this cluster, members at the family level are found dispersed throughout the network, including one *Xanthobacteraceae* and 42 *Methylocystaceae*.

We have proposed^4^ several hallmarks of a LanM. First, LanMs possess four EF-hand motifs. EF hands comprise 12-residue, carboxylate-rich metal-binding loops flanked by α-helices, which traditionally respond to Ca^II^ binding;^29^ in *Mex*-LanM, however, EF hands 1–3 bind lanthanide(III) ions with low-picomolar affinity and 10^8^-fold selectivity over Ca^II^, resulting in a large, lanthanide-selective disorder-to-order conformational transition^4^. EF4 binds with only micromolar affinity. Second, adjacent EF hands in LanMs are separated by 12–13 residues—rather than the typical ≈25 residues in Ca^II^-responsive EF-hand proteins—resulting in an unusual three-helix bundle architecture with the metal-binding sites on the periphery^5^. Third, at least one EF hand contains proline at the second position (in *Mex*-LanM, all four EF hands feature P_2_ residues). We searched sequence databases using the first two criteria and a sequence length of <200 residues, identifying 696 putative LanMs. These sequences were visualized using a sequence similarity network^30^ to identify LanM sequences that cluster separately from *Mex*-LanM. Notably, at a 65% identity threshold, a small cluster of sequences that is remote from the main cluster of 642 sequences is formed (Fig. 1a). This exclusive cluster (the *Hans* cluster), includes bacteria from several genera, including *Hansschlegelia* and *Xanthobacter* (Extended Data Fig. 1), all of which are facultative methylotrophs^31^.

*Hans*-LanM features low (33%) sequence identity with *Mex*-LanM (Supplementary Fig. 1) and divergent EF-hand motifs, particularly at the first, second and ninth positions (Fig. 1b), which are important positions in *Mex*-LanM^23,26^ and other EF-hand proteins^29^. Therefore, *Hans*-LanM presented an opportunity to determine features essential for selective lanthanide recognition in LanMs.

*Hans*-LanM was expressed in *Escherichia coli* as a 110 amino acid protein (Supplementary Fig. 1). La^III^ and Nd^III^ were selected as representative LREs and Dy^III^ was selected as a representative HRE for complexation studies. The protein binds about three equivalents of La^III^ and Nd^III^, and slightly less Dy^III^, by inductively coupled plasma mass spectrometry (Supplementary Table 1), as does *Mex*-LanM^4^. Also like *Mex*-LanM^4^, *Hans*-LanM exhibits little helical content in the absence of metal, as judged by the circular dichroism signal at 222 nm (Fig. 1c). Unexpectedly, only two equivalents of La^III^ or Dy^III^ were sufficient to cause *Hans*-LanM’s complete conformational change (Supplementary Fig. 2), indicating that the third binding equivalent is weak and does not increase helicity.

**Extended Data Fig. 2:**
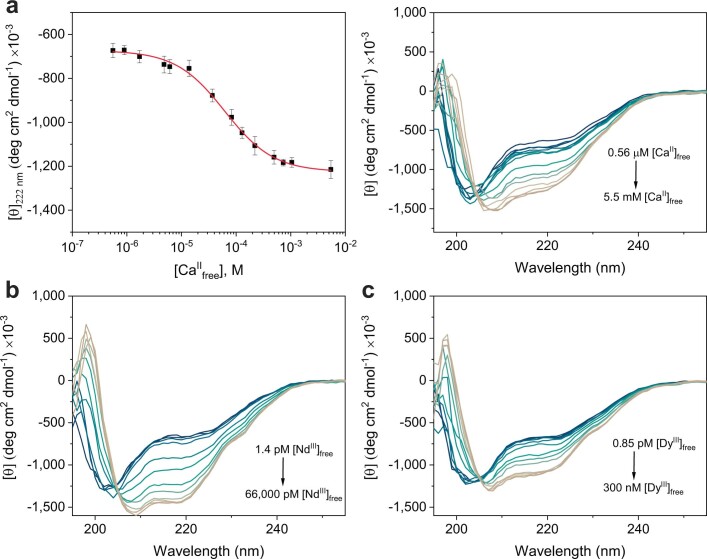
Circular dichroism titrations of *Hans*-LanM with chelator-buffered solutions of (a) Ca^II^, (b) Nd^III^, and (c) Dy^III^. Both Dy^III^ (up to 0.3 µM) and Ca^II^ (up to 5.5 mM) induce a similar, incomplete conformational change in the protein, relative to the conformational change induced by Nd^III^ and La^III^. The data in the right panel of **a** is a representative titration from the 3 datasets used to generate the plot in the left panel. The data in **b** and **c** are representative titrations from the 3 datasets used to generate the plot in Fig. 1d. Conditions: 15 μM protein, 20 mM acetate, 100 mM KCl, 10 mM EDTA (for Ca and Nd titrations) or EGTA (for Dy titration), 0—10 mM metal ion. Each data point in **(a**, left panel) is the mean ± s.d. for three independent measurements. Source Data

The apparent dissociation constants (*K*_d,app_) determined by circular dichroism spectroscopy^4^ reflect the RE versus RE, and RE versus non-RE, selectivities of *Mex*-LanM under competitive RE recovery conditions^6,18^. Therefore, similar determinations of *K*_d,app_ with free metal concentrations controlled by a competitive chelator^4,32^ were applied to *Hans*-LanM; the results (Fig. 1d and Supplementary Table 2) diverged from those for *Mex*-LanM. Binding of La^III^ and Nd^III^ to *Hans*-LanM increases molar ellipticity at 222 nm by 2.3-fold, the full conformational change evident in stoichiometric titrations. The conformational change is cooperative (Hill coefficients, *n*, of 2; Supplementary Table 2), and the *K*_d,app_ values are similar, 68 and 91 pM, respectively. By contrast, even though Dy^III^ induces the same overall response as La^III^ in stoichiometric titrations (Supplementary Fig. 2), in the chelator-buffered Dy^III^ titrations *Hans*-LanM exhibits a lesser conformational response (1.8-fold increase). This difference indicates that at least one of the Dy^III^-binding sites is very weakly responsive (*K*_d,app_ > 0.3 µM, the highest concentration accessible in the chelator-buffered titrations). The main response to Dy^III^ occurs at 2.6 nM, >30-fold higher than with the LREs, and with little or no cooperativity (*n* = 1.3). By contrast, *Mex*-LanM shows only a modest preference for LREs (about fivefold; Fig. 1e; ref. ^4^), and all lanthanides and Y^III^ induce similar conformational changes and cooperativity^18^. *Hans*-LanM responds to calcium(II) weakly (*K*_d,app_ = 60 µM), with the same lack of cooperativity (*n* = 1.0) and partial conformational change evident with Dy^III^ (Extended Data Fig. 2). Therefore, *Hans*-LanM discriminates more strongly between LREs and HREs than does *Mex*-LanM, with the HRE complexes exhibiting lower affinity, lesser cooperativity and a lesser primary conformational change.

## LRE-selective dimerization

**Fig. 2:**
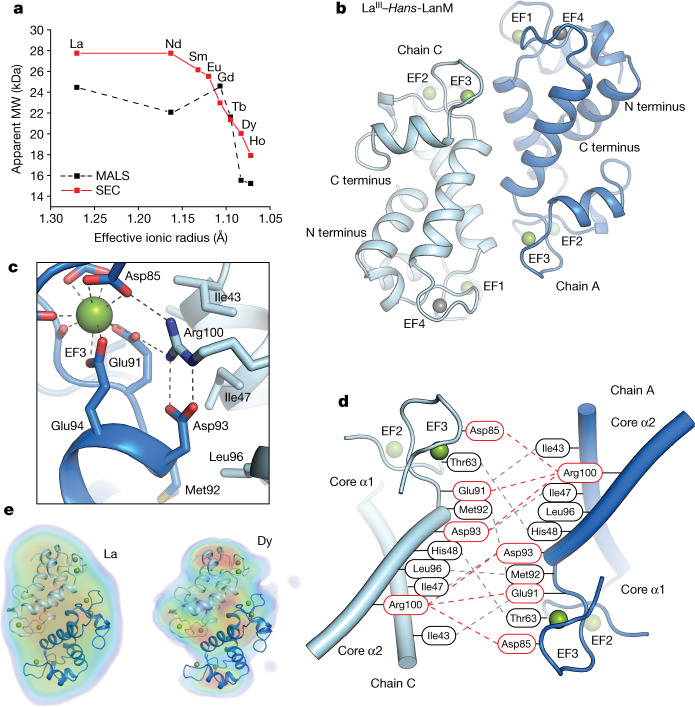
A dimerization equilibrium sensitive to LRE versus HRE or non-RE coordination. **a**, Apparent molecular weight of *Hans*-LanM complexes with REs as determined by analytical SEC (red lines) or SEC–MALS (black dashed line). See Supplementary Table 1 for conditions. Each individual data point is the result of a single experiment. **b**, The La^III^-bound *Hans*-LanM dimer as determined by X-ray crystallography. La^III^ ions are shown as green spheres and Na^I^ ions are shown as grey spheres. **c**, Detailed view of the dimer interface near EF3 of chain A (blue cartoon). Arg100 from chain C (light blue cartoon) anchors a hydrogen-bonding network involving Asp93 of chain A and two EF3 La^III^ ligands (Glu91 and Asp85). These interactions constitute the sole polar contacts at the dimer interface, providing a means to control the radius of the lanthanide-binding site at EF3. **d**, Schematic of the interactions at the dimer interface. Red dashed lines indicate hydrogen-bonding interactions and grey dashed lines indicate hydrophobic contacts. **e**, DENSS projections of electron density from small-angle X-ray scattering datasets for La^III^-bound (left) and Dy^III^-bound (right) *Hans*-LanM, overlaid with a PyMOL-generated ribbon diagram of the dimeric La^III^–*Hans*-LanM crystal structure.

The distinct behaviours of the LRE– and HRE–*Hans*-LanM complexes suggested mechanism(s) of LRE versus HRE selectivity not present in *Mex*-LanM. As *Mex*-LanM is a monomer in complex with LREs and HREs alike^4,5^, we considered that LREs and HREs might induce different oligomeric states in *Hans*-LanM. In the presence of three equivalents of La^III^, *Hans*-LanM elutes from a size-exclusion chromatography (SEC) column not at the expected molecular weight (MW) of 11.9 kDa but instead at 27.8 kDa, suggestive of a dimer (Supplementary Figs. 3 and 4a). Starting gradually after Nd^III^ but sharply at Gd^III^, the apparent MW decreases towards that expected for a monomer (Fig. 2a, Supplementary Fig. 4 and Supplementary Table 3). Notably, lanthanides heavier than Gd^III^ do not seem to support growth of RE-utilizing bacteria^33–35^.

To provide further support for preferential dimerization in the presence of physiologically relevant LREs, RE complexes of *Hans*-LanM were analysed using multi-angle light scattering (MALS; Fig. 2a and Supplementary Fig. 5). The La^III^, Nd^III^ and Gd^III^ complexes have MWs of 22–25 kDa, indicative of dimers, but MWs decrease starting with Tb^III^ and continue to Dy^III^ and Ho^III^, at about 15 kDa (Extended Data Table 1), in agreement with the SEC data. Ca^II^-bound *Hans*-LanM also indicated a MW of 14.7 kDa. The HRE–, Ca^II^– and apo *Hans*-LanM complexes are still one-third larger than expected for a monomer, however, suggesting that these forms exist in a rapid equilibrium with ≈2:1 monomer/dimer ratio under these conditions. We next determined the *K*_d_ for dimerization (*K*_dimer_) of apo, La^III^-bound and Dy^III^-bound *Hans*-LanM by isothermal titration calorimetry (Extended Data Table 2 and Supplementary Figs. 6–8). The apoprotein and Dy^III^-bound protein weakly dimerize, with *K*_dimer_ values of 117 µM and 60 µM, respectively, consistent with the ratios of monomer and dimer reflected in the SEC and MALS traces. In the presence of La^III^, however, the dimer was too tight to be able to observe monomerization by isothermal titration calorimetry, which indicates that *K*_dimer_ <0.4 µM (Supplementary Fig. 8). Thus, La^III^ favours *Hans*-LanM’s dimerization by >100-fold over Dy^III^.

**Extended Data Fig. 3:**
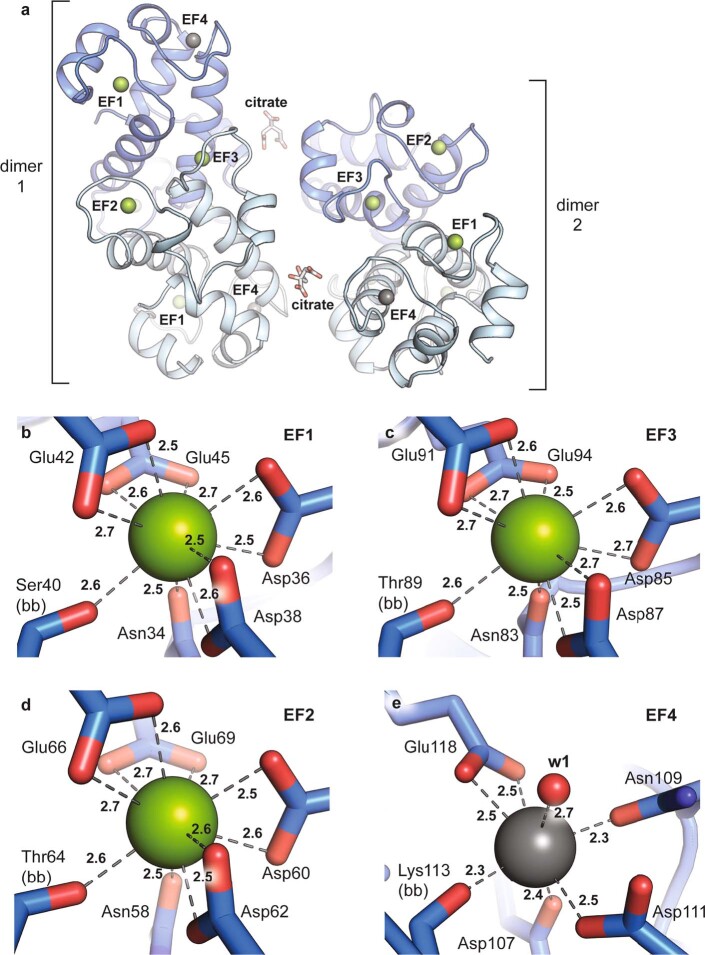
X-ray crystal structure of La^III^-bound *Hans*-LanM, solved at 1.8 Å resolution. **a**, Overall structure of the asymmetric unit, which consists of two *Hans*-LanM dimers and two citrate molecules from the crystallization solution. The structure of each monomer of the dimer is consistent with the NMR solution structure of Y^III^-bound *Mex*-LanM with EF-hands 2 and 3 paired and EF-hands 1 and 4 paired^5^. **b–e**, Details of metal coordination in the four EF-hands of La^III^-*Hans*-LanM. The coordination spheres of the La^III^ ions in EF-hands 1, 2, and 3 are constituted by the side chain Oδ of N_1_ (monodentate), the carboxylate side chains from D_3_, D_5_, E_9_, and E_12_ (all bidentate), and a backbone carbonyl from S_7_ (EF1) or T_7_ (EF2 and EF3), for a total coordination number of 10. All La^III^-ligand distances are 2.5-2.7 Å. The crystal radius for 10-coordinate La^III^ is given as 1.41 Å by Shannon;^7^ given 1.26 Å as radius of 6-coord O^2-^, 2.57 Å is estimated for the La^III^-O distance, consistent with our results. The metal ion in EF4 was modeled as Na^I^ because of the shorter metal-ligand distances, lower coordination number, and the presence of sodium in the crystallization solution. Ca^II^ cannot be completely ruled out as it was present earlier in the protein purification; however, the protein was treated with Chelex at the end of the purification, and the crystallographic data were consistent with the Na^I^ assignment as determined by the CheckMyMetal server^65^. This ion is coordinated in distorted pentagonal bipyramidal geometry by monodentate D_1_, N_3_, and D_5_ sidechains, the bidentate E_12_ sidechain, the backbone carbonyl of Lys113, and a single solvent molecule for a total coordination number of 7. The Na^I^-protein ligand distances are 2.3-2.5 Å, with a solvent molecule at 2.7 Å. In the case of *Mex*-LanM, biochemical data^4,26^ and NMR spectroscopy^5^ have also supported EF4 as a poor lanthanide-binding site, and it was modeled without a metal ion in the NMR solution state structure^5^.

A 1.8-Å-resolution X-ray crystal structure of *Hans*-LanM in complex with La^III^ confirms LRE-induced dimerization (Extended Data Fig. 3 and Supplementary Table 4). Two LanM monomers interact head-to-tail (Fig. 2b), burying about 600 Å^2^ of surface area through hydrophobic and polar contacts (Fig. 2c,d). These interactions occur largely between side chains contributed by the core helices α1 (between EF1 and EF2) and α2 (between EF3 and EF4; Supplementary Fig. 9). Residues at the dimer interface make direct contact with only one of the four metal-binding sites, EF3; three residues of EF3 in each monomer form a hydrogen-bonding network with Arg100 of the other monomer (Fig. 2c), suggesting that occupancy and coordination geometry at this site may control oligomeric state.

*Hans*-LanM and its complexes with three equivalents of La^III^, Nd^III^ and Dy^III^ were also analysed by small-angle X-ray scattering (Supplementary Figs. 10 and 11). The calculated solvent envelopes^36^ from the small-angle X-ray scattering data fit well to the crystallographic *Hans*-LanM dimer for La^III^–*Hans*-LanM, adequately for Nd^III^–*Hans*-LanM, but poorly for Dy^III^–*Hans*-LanM (Fig. 2e and Supplementary Figs. 12–14). The weaker dimerization of Dy^III^–*Hans*-LanM is also supported by quantitative metrics, such as the Porod volume (Supplementary Figs. 15 and 16 and Supplementary Tables 5 and 6). Together, the biochemical and structural results indicate that *Hans*-LanM’s dimerization equilibrium depends strongly on the particular RE bound.

## Structural basis for dimerization

The structure of La^III^–*Hans*-LanM also provides one of the first detailed views of the coordination environments in a LanM, and indeed any natural biomolecule tasked with reversible lanthanide recognition. The previous NMR structure of *Mex*-LanM^5^ revealed the protein’s unusual fold, but it could not provide molecular-level details about the metal-binding sites. To understand the basis for LRE versus HRE discrimination, we also determined a 1.4-Å-resolution structure of Dy^III^–*Hans*-LanM. Finally, we report a 1.01-Å-resolution structure of Nd^III^–*Mex*-LanM, which rationalizes *Mex*-LanM’s shallower RE selectivity trend^4^.

**Fig. 3:**
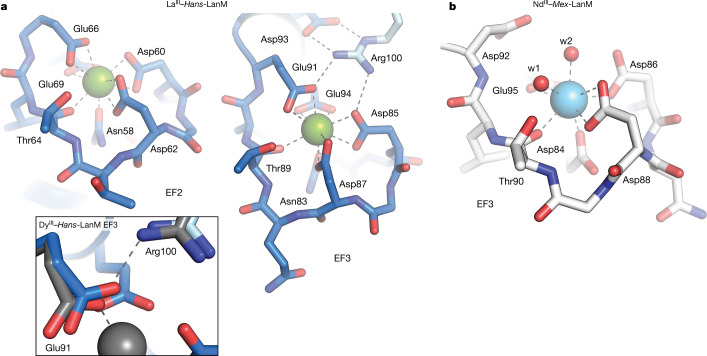
*Hans*-LanM uses an extended hydrogen-bonding network to control lanthanide selectivity. **a**, Zoomed-in views of EF2 (left) and EF3 (right) in La^III^–*Hans*-LanM. La^III^ ions are shown as green spheres. Coordination bonds and hydrogen bonds are shown as dashed lines. Residues contributed by chain A are shown in blue and those contributed by chain C (in the case of EF3) are shown in light blue. Inset: overlay of La^III^–*Hans*-LanM (blue and light blue) with Dy^III^–*Hans*-LanM (grey), showing the carboxylate shift of Glu91 from bidentate (La) to monodentate (Dy). Coordination and hydrogen bonds (dashed lines) are shown only for the Dy case. **b**, Representative metal-binding site (EF3) in Nd^III^–*Mex*-LanM. Nd^III^ ion is shown as an aqua sphere. Solvent molecules are shown as red spheres.

In La^III^–*Hans*-LanM, EF1–3 are occupied by La^III^ ions (Extended Data Fig. 3b–e). EF4 is structurally distinct, does not exhibit anomalous difference density consistent with La^III^ and was modelled with Na^I^ (Supplementary Fig. 17a). Each La^III^-binding site is ten-coordinate, as observed in structures of lanthanide-dependent methanol dehydrogenases^33,37^ (Supplementary Fig. 18). A monodentate Asn (N_1_ position), four bidentate Asp or Glu residues (D_3_, D_5_, E_9_ and E_12_) and a backbone carbonyl (T_7_ or S_7_) complete the first coordination sphere in EF1–3 (Fig. 3a). Exogenous solvent ligands are not observed (Supplementary Fig. 17b); luminescence studies of Eu^III^–*Hans*-LanM to determine the number of coordinated solvent molecules (*q*) yielded *q* = 0.11, consistent with the absence of solvent ligands in the X-ray structure (Supplementary Fig. 19).

The lanthanide-binding sites in *Hans*-LanM additionally share extensive second-sphere interactions that may further constrain the positions of the ligands and the size of the metal-binding pore (Supplementary Fig. 20). This phenomenon is most obvious in EF3, at which the dimer interface mediates an extended hydrogen-bonding network involving several ligands. Arg100, contributed by the adjacent monomer, projects into the solvent-exposed side of EF3 to contact two carboxylate ligands, Asp85 (D_3_) and Glu91 (E_9_), enforcing their bidentate binding modes. Arg100 is also buttressed by Asp93 (EF3 D_11_), unique to EF3 within *Hans*-LanM and not observed in *Mex*-LanM. We tested the importance of this network in *Hans*-LanM dimerization by making the minimal substitution, R100K. *Hans*-LanM(R100K) had nearly identical *K*_d,app_ values and response to Nd^III^ and Dy^III^ as wild-type *Hans*-LanM, but the *K*_d,app_ for La^III^ was twofold weaker (Supplementary Fig. 21 and Supplementary Table 7). SEC–MALS analysis indicated MWs of 10–13 kDa for apo, La^III^– and Dy^III^–*Hans*-LanM(R100K) (Supplementary Fig. 22 and Supplementary Table 8), indicative of increased monomerization, especially for the La^III^ complex, and suggesting that weaker dimerization may be responsible for the lower La^III^ affinity. All four residues comprising the Arg100–EF3 network are completely conserved in the *Hans* cluster (Supplementary Fig. 23), suggesting that these interactions may contribute to dimerization in these LanMs.

**Extended Data Fig. 4:**
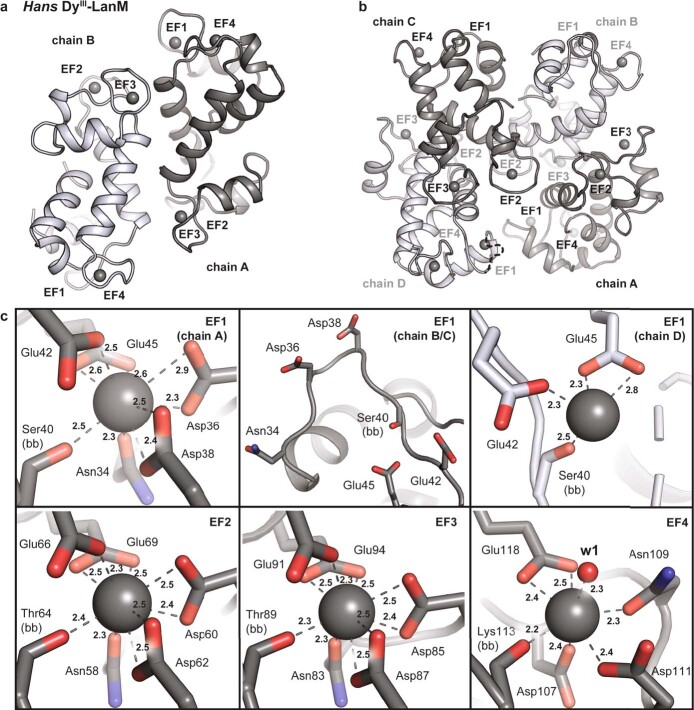
X-ray crystal structure of Dy^III^-bound *Hans*-LanM, solved at 1.4 Å resolution. **a**, One of the dimers in the asymmetric unit, comprising chains A and B. Note that EF4 is unexpectedly occupied with Dy^III^ while EF1 is occupied only in chain A. **b**, Overall structure of the asymmetric unit, which consists of two *Hans*-LanM dimers. Unlike the La^III^-*Hans*-LanM structure, the two dimers – and the monomers within each dimer – display significant differences in Dy^III^-*Hans*-LanM. EF2-4 are occupied by Dy^III^ in all chains, whereas EF1 is only occupied and ordered in chain A; in chains B and C, no metal ion is bound in the EF-hand, and in chain D, a Dy^III^ ion is bound but the first five residues of EF1 (Asn34 – Asp38) could not be modeled. Our decision to model Dy^III^ into all four EF-hands is supported by anomalous diffraction datasets (Supplementary Tables 9–10, Supplementary Figs. 25–26). The biochemical data suggest that, in solution, at least one Dy^III^ binding site is weak (see Fig. 1d and Supplementary Fig. 2), and it is likely based on studies of *Mex-*LanM that EF2/3 are the tighter binding sites. This proposal is supported by the Dy anomalous data (Supplementary Table 10), and the occupancy of weak metal-binding sites likely results from the high protein concentration used for crystallography. **c**, Details of metal coordination in the EF-hands of Dy^III^-*Hans*-LanM. In the top row, the three different EF1 structures in the asymmetric unit are shown. Only in chain A is the EF1 metal site nearly identical to the sites in EF2 and EF3 (contrary to La^III^-*Hans*-LanM, where EF1-3 sites are very similar, Extended Data Fig. 3). In EF1 (chain A), EF2, and EF3, the coordinating ligands are the same as with La^III^-*Hans*-LanM, except that the E_9_ residues (Glu42, Glu66, and Glu91) have shifted to monodentate coordination, resulting in 9-coordination. The lower coordination number with Dy^III^ is consistent with the lanthanide contraction^68,69^ and is observed with other ligands (as one recent example, ref. ^39^). The Dy^III^-ligand distances are mostly 2.3-2.5 Å, ~0.2 Å shorter than for La^III^-*Hans*-LanM. Consistent with this observation, the crystal radius for 9-coordinate Dy^III^ is given as 1.22 Å by Shannon^7^, 0.19 Å shorter than for 10-coordinate La^III^ (Extended Data Fig. 3). The carboxylate shift of the 9^th^ position Glu residue is noteworthy as this position is important for gating affinity and selectivity in other EF-hand proteins^70^. In EF4, Dy^III^ is 7-coordinate with pentagonal bipyramidal geometry, similar to the sodium site in La^III^-*Hans*, but with slightly shorter metal-ligand distances (2.2-2.5 Å); again, these distances are consistent with the expectation for 7-coordinate Dy^III^ (ref. ^7^).

The structure of Dy^III^–*Hans*-LanM confirms the importance of second-sphere control of ligand positioning (Extended Data Fig. 4, Supplementary Figs. 24–26 and Supplementary Tables 9 and 10). The overall structure of Dy^III^–*Hans*-LanM is largely superimposable with that of La^III^–*Hans*-LanM, and the coordination spheres of the Dy^III^ ions in EF1–3 are similar to those in La^III^–*Hans*-LanM (Fig. 3a, inset), with the notable exception of E_9_ (for example, Glu91 in EF3). This residue shifts from bidentate with La^III^ to monodentate with the smaller Dy^III^ ions, yielding a nine-coordinate distorted capped square antiprismatic geometry; the lower coordination number with a HRE ion is consistent with the case of other RE complexes^38,39^. In EF3, this carboxylate shift lengthens the distance between Arg100 and the proximal Oε of Glu91 from 2.9 Å (in La^III^–*Hans*-LanM) to 3.2 Å (Supplementary Fig. 27). The rearrangement of this second-sphere hydrogen-bonding network suggests a structural basis for RE-dependent differences in *K*_dimer_ values.

The metal-binding sites of *Mex*-LanM differ substantially from those of *Hans*-LanM. In *Mex*-LanM, all four EF hands are occupied by nine-coordinate (EF1–3) or ten-coordinate (EF4) Nd^III^ ions, each including two solvent ligands, not present in *Hans-*LanM (Fig. 3b and Supplementary Fig. 28). The observation of the two solvent molecules per metal site and the hydrogen bond to the D_9_ residue validates recent spectroscopic studies^21,23,26^. The difference in coordination number between EF1–3 and EF4 is due to the D_3_ carboxylate being monodentate in EF1–3 but bidentate in EF4. Although the Nd^III^ sites of *Mex*-LanM share the nine- and ten-coordination observed in Dy^III^– and La^III^–*Hans*-LanM, they structurally resemble the seven-coordinate Ca^II^-binding sites of calmodulin (Supplementary Fig. 18). The increased coordination numbers in *Mex*-LanM relative to calmodulin result from bidentate coordination of D_5_ and an additional solvent ligand. These similarities suggest that much of LanM’s unique 10^8^-fold selectivity for REs over Ca^II^ results from subtle differences in second-coordination-sphere and other more distal interactions. Finally, the exclusively protein-derived first coordination sphere in *Hans*-LanM, particularly due to coordination by E_9_, yields more extended hydrogen-bonding networks (Supplementary Figs. 20 and 29) and probably enhances control over the radius of the binding site. Thus, the structures rationalize the extraordinary RE versus non-RE selectivity of *Mex*-LanM and *Hans*-LanM while also accounting for their differences in LRE versus HRE selectivity.

## Single-stage Nd^III^/Dy^III^ separation

**Fig. 4:**
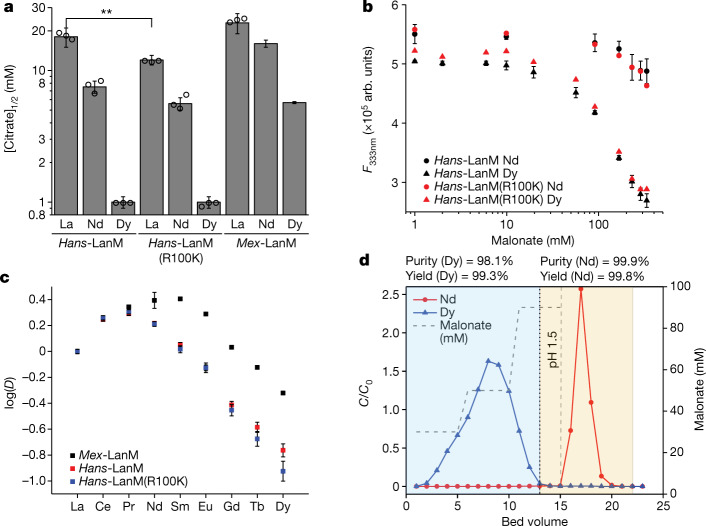
Leveraging *Hans*-LanM to separate Nd/Dy in a single-stage process. **a**, *Hans*-LanM and the R100K variant exhibit greater differences in Nd versus Dy complex stability than *Mex*-LanM against desorption by citrate. Mean ± s.e.m. for three independent trials. **Significant difference between [citrate]_1/2_ for La^III^ between *Hans*-LanM and *Hans*-LanM(R100K) (20 µM protein) shows the impact of dimerization of La^III^ complex stability (*P* < 0.01, analysis of variance with Bonferroni post-test). *Mex*-LanM Nd and Dy data from ref. ^6^. **b**, Spectrofluorometric titration of *Hans*-LanM and R100K variant (*λ*_ex_ = 280 nm, *λ*_em_ = 333 nm) at pH 5.0, depicting the malonate-induced desorption of a 2:1 metal–protein complex. Mean ± s.e.m. for three independent trials, except those with R100K, which were single trials of each condition. **c**, Comparison of distribution factors (pH 5.0, about 0.33 mM each RE, La^III^–Dy^III^) for immobilized *Hans*-LanM, *Hans*-LanM(R100K) and *Mex*-LanM. Each point represents mean ± s.d. for three independent trials. **d**, Separation of a 95:5 mixture of Nd^III^/Dy^III^ using immobilized *Hans*-LanM(R100K) and a desorption scheme of three stepped concentrations of malonate followed by pH 1.5 HCl. One bed volume was 0.7 ml. Source Data

**Extended Data Fig. 5:**
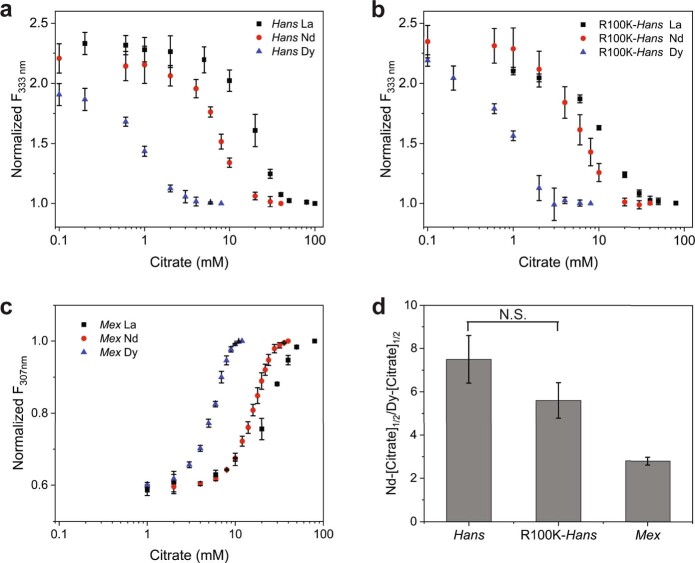
Spectrofluorometric titrations of RE-LanM (*Hans*-LanM, *Hans*-LanM(R100K), and *Mex*-LanM) complexes with citrate as a competitor, monitored by intrinsic protein fluorescence. Emission values are normalized to 1.0 for the fluorescence of the apoprotein. Note that the fluorescence intensity of *Hans*-LanM’s Trp residues decreases going from the RE-bound to apo state (Supplementary Fig. 30), whereas the intensity of *Mex*’s Tyr residue increases going from the RE-bound to apo state^4,6^. Initial conditions: 20 μM protein, 40 μM RE, 20 mM acetate, 100 mM KCl, pH 5.0, for all experiments, into which increasing concentrations of citrate were titrated. The citrate concentrations at which 50% of each metal is desorbed under these conditions ([citrate]_1/2_) are summarized in Supplementary Table 11 and plotted in Fig. 4a. **a**, *Hans*-LanM. **b**, *Hans*-LanM(R100K). The compressed difference between the [citrate]_1/2_ values for La and Nd in *Hans-*LanM(R100K) vs. wild-type *Hans*-LanM illustrates the role of dimerization in enhancing affinity differences for the LREs, especially La^III^. **c**, *Mex-*LanM. Nd and Dy data were reported in Dong et al^6^. **d**, Comparison of the ratios of [citrate]_1/2_ value for Nd to that for Dy, for each protein, illustrating the greater Nd/Dy selectivity of *Hans*-LanM relative to *Mex*-LanM. The ratios for wild-type *Hans*-LanM and the R100K variant are not significantly different (*p* > 0.05) by two-tailed t-test, suggesting that the hydrogen-bonding network involving Arg100 contributes relatively little to Nd^III^/HRE selectivity, though it does impact La^III^ selectivity significantly (Fig. 4a). All data are shown as mean ± s.d. (a—c) or s.e.m. (d) for data from 3 independent experiments. Source Data

The differences in stability and structure between *Hans-*LanM’s LRE versus HRE complexes suggested that *Hans*-LanM (wild type and/or R100K) would outperform *Mex*-LanM in RE/RE separations. We focused on separating the RE pair of Nd^III^ and Dy^III^ used in permanent magnets. We first assayed the stabilities of the wild-type *Hans*-LanM and *Hans*-LanM(R100K) RE complexes against citrate, previously used as a desorbent with *Mex*-LanM^6^. RE–*Hans*-LanM complexes are generally less stable against citrate than those of *Mex*-LanM, as expected on the basis of lower affinity (Fig. 1e), but the difference in stability between the Nd^III^–*Hans*-LanM and Dy^III^–*Hans*-LanM complexes—expressed as the ratio of citrate concentration required for 50% desorption of each metal ([citrate]_1/2_), as reported by the fluorescence of *Hans*-LanM’s two Trp residues (Supplementary Fig. 30)—is twofold greater than for *Mex*-LanM complexes (Fig. 4a, Supplementary Table 11 and Extended Data Fig. 5). Furthermore, the R100K substitution significantly destabilizes *Hans*-LanM’s La^III^ complex against citrate, whereas it only slightly affects the Nd^III^ complex and does not affect the Dy^III^ complex. This result confirms that dimerization selectively stabilizes *Hans*-LanM’s LRE complexes (and especially the La^III^ complex), a factor abrogated by the R100K substitution. Using malonate, a weaker chelator than citrate, Dy^III^ can be readily desorbed from both *Hans*-LanM and R100K with 10–100 mM chelator without significant Nd^III^ desorption, suggesting conditions for Nd^III^/Dy^III^ separation (Fig. 4b).

**Extended Data Fig. 6:**
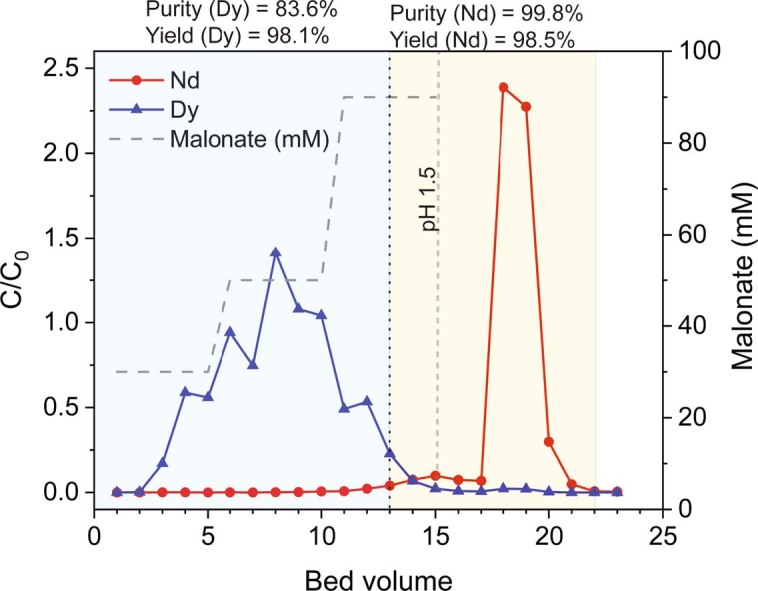
Separation of a 95:5 mixture of Nd:Dy using immobilized *Hans*-LanM. The desorption scheme consisted of three stepped concentrations of malonate (30, 50, 90 mM; see right axis) followed by pH 1.5 HCl. The results revealed that slightly lower purity Dy was generated using *Hans*-LanM compared to the R100K variant (83.6% vs 98% Dy purity at similar yield, respectively; compare to Fig. 4d). While similar selectivity profiles were observed for the immobilized proteins for La through Gd in equilibrium binding experiments with La-Dy, the selectivity pattern diverged at Tb (Fig. 4c). The selectivity difference between *Hans*-LanM and the R100K variant was confirmed by using a Nd/Dy binary system, as the uncertainties in the distribution factor determination for Dy in the 9-element RE group precluded the ability to distinguish small differences in the Dy/Nd separation factor between proteins (Supplementary Tables S13–S14). In this binary Nd/Dy experiment (Extended Data Table 3), we determined a separation factor of 8.12 ± 0.40 for *Hans*-LanM and 12.7 ± 1.3 for the R100K variant, which is consistent with the improved Dy separation efficacy of R100K. While consistent with the values derived from the 9-element experiment, the results differ slightly from the equilibrium binding results with the free *Hans*-LanM and *Hans*-LanM(R100K) proteins, which revealed similarly high selectivity for Nd over Dy (Fig. 4a,b), likely reflecting weaker LRE-induced dimerization in the R100K variant at the low protein concentration (20 µM) of the solution experiments with free protein. The La/Nd selectivity on-column is also distinct from that observed with the apparent *K*_d_ values of the free proteins (wild-type and R100K) in solution, although the experiments with free proteins utilized single element solutions and effects from mixed metal binding may impact the on-column data. The R100K variant is also better behaved on the column, as evidenced by the 2:1 RE:protein stoichiometry. One possible explanation for these results could be that immobilization interferes with dimerization; however, Fig. 2b shows the N- and C-termini of the *Hans*-LanM dimer, indicating that the C-termini are ~20 Å from the nearest part of the dimer interface, suggesting that immobilization per se would not be expected to disrupt this interface. It must be considered, however, that a functional dimer would require two C-termini to be immobilized in close proximity, which is unlikely at the immobilization densities of our columns. Therefore, on balance, we suspect that the dimerization equilibrium is only applicable in a minority of protein units immobilized on the column. We posit that more fully exploiting the dimerization equilibrium in the column format would yield even more robust separations. The surest way to obtain homogeneous populations of dimers on-column would likely be to link two monomers together (e.g., with a polypeptide chain), tuning dimerization affinity through mutagenesis of the residues contributing to inter-monomer interactions, and immobilizing this dimer through a single attachment point. Dimerization could also be exploited in other separation formats. These directions are the subject of current efforts. Source Data

Although a twofold modulation of RE versus RE selectivity by dimerization may seem small, such differences provide opportunity to decrease the number of separation stages, increasing efficiency of a separation process^3,12^. Therefore, *Hans*-LanM and the R100K variant were immobilized through a carboxy-terminal Cys residue on maleimide-functionalized agarose beads, as described previously^6^, and tested for Nd^III^/Dy^III^ separation. Immobilized *Hans*-LanM bound about one equivalent of RE, unlike in solution and compared with two equivalents for *Mex-*LanM^6^ and *Hans*-LanM(R100K) (Supplementary Fig. 31). *Hans*-LanM and R100K exhibited similar separation ability in the La–Gd range—although R100K exhibits greater separation ability in the Gd–Dy range—as determined by the on-column distribution ratios (*D*) of a mixed RE solution at equilibrium (Fig. 4c, Extended Data Table 3 and Supplementary Tables 12–14). These Nd/Dy separation factors are nearly double (*Hans*-LanM) and triple (*Hans*-LanM(R100K)) that of *Mex*-LanM (Extended Data Table 3). Immobilized *Hans*-LanM was loaded to 90% of breakthrough capacity with a model electronic waste mixture of 5% dysprosium and 95% neodymium and, guided by Fig. 4b, eluted with a short, stepwise malonate gradient, followed by complete desorption using pH 1.5 HCl. In a single purification stage, Dy was upgraded from 5% to 83% purity and Nd was recovered at 99.8% purity (both >98% yield; Extended Data Fig. 6). This significantly outperformed the comparable *Mex*-LanM-based process, which achieved only 50% purity in a first separation stage and required a second stage to obtain >98% purity^6^. The immobilized R100K variant performed even better, achieving baseline separation of Dy^III^ and Nd^III^ to >98% purity and >99% yield in a single stage (Fig. 4d). The R100K variant’s better performance was unexpected and may point to the unlikelihood of functional dimers on the column at this immobilization density (see the caption of Extended Data Fig. 6 for a discussion). Thus, despite substantially improved performance versus *Mex*-LanM enabled by characterization of *Hans*-LanM’s mechanism of dimerization, fully exploiting the dimerization phenomenon on-column may involve, for example, tethering of two monomers on a single polypeptide chain, which is under investigation.

## Conclusion

Biochemical and structural characterization of *Hans*-LanM’s mechanism of metal-sensitive dimerization provides a new, allosteric mechanism for LRE versus HRE selectivity in biology, extending concepts in dimer-dependent metal recognition recently emerging from synthetic lanthanide complexes^11^ and engineered transition metal-binding proteins^40^ and showing that these principles are hard-wired into nature. Our work also shows that dimerization strength, and thus metal selectivity, can be rationally modulated. *Hans*-LanM evolved LRE-selective dimerization at physiological protein concentrations closer to those in our biochemical assays (10–20 µM) rather than those on the column (about 3 mM). Therefore, leveraging dimerization in a separation process would be assisted by shifting dimerization sensitivity to the higher concentration regime, such as by tuning hydrophobic interactions at the dimerization interface. Furthermore, our studies establish that LanMs with as low as 33% identity are easily predicted yet have useful differences in metal selectivity; further mining of this diversity may reveal yet additional mechanisms for tuning RE separations. Finally, the solvent-excluded coordination spheres of *Hans*-LanM should outperform *Mex*-LanM in RE/actinide separation^23^, luminescence-based sensing^21,26^ and stabilization of hydrolysis-prone ions. Continued characterization of the coordination and supramolecular principles of biological f-element recognition will inspire design of ligands with higher RE versus RE selectivities and their implementation in new RE separation processes.

## Methods

### General considerations

See the Supplementary Methods for details.

### Bioinformatics methods

#### Protein and genome sequence data

The sequence of LanM from *M. extorquens* AM1 was used as a query to conduct PSI-BLAST searches against the National Center for Biotechnology Information non-redundant protein sequence (nr) and metagenomic protein (env_nr) databases until convergence^41^. The resulting 3,047 protein sequences were then manually curated for those that are less than 200 residues long, have at least one pair of EF hands separated by less than 14 residues, and have 4 EF hands. Signal peptides of LanM sequences were predicted using SignalP (v6.0)^42^, and then removed before further analysis of the sequences.

#### Construction of sequence similarity networks

The Enzyme Function Initiative-Enzyme Similarity Tool was used to calculate the similarities between all peptide sequence pairs with an *E*-value threshold of 1 × 10^−5^ (ref. ^30^). The resulting sequence similarity network of 696 nodes and 241,853 edges was then constructed and explored using the organic layout through Cytoscape (v3.9.1)^43^ and visualized in R (v4.1.0)^44^. The edge percentage identity threshold was gradually increased from 40% to 90% to yield distinct clusters.

#### Multiple sequence alignment and phylogenetic analysis

LanM sequences were aligned using MUSCLE (v5.1)^45^ with default parameters. The model used for phylogeny construction was selected using ModelFinder in IQ-TREE (v2.2.0.3)^46,47^ with --mset set to beast2. Bayesian phylogeny was generated on the basis of these results using BEAST (v2.6.7)^48^. The resulting phylogeny was evaluated using 10^7^ generations and discarding a burn-in of 25%, and then visualized using ggtree (v3.2.1)^49^.

### Expression and purification of *Hans*-LanM and its R100K variant

The gene encoding *Hans-*LanM, codon optimized for expression in *E. coli* without its native 23-residue signal peptide (see Supplementary Table 15), was obtained from Twist Bioscience and inserted into pET-29b(+) using the restriction sites NdeI/XhoI. *Hans*-LanM was overexpressed on a 2-l scale and purified using the established protocol for *Mex*-LanM^50^, with one modification: after the final SEC step, the protein was concentrated to 5 ml and dialysed against 5 g Chelex 100 in 500 ml of 30 mM HEPES, 100 mM KCl, 5% glycerol, pH 8.4, to remove Ca^II^ and trace metal contaminants. This procedure resulted in approximately 15 ml of 550 μM protein, which was not concentrated further. The final yield was 45 mg of protein per litre of culture. Protein concentrations were calculated using an extinction coefficient of 11,000 M^−1^ cm^−1^, based on the ExPASy ProtParam tool^51^. *Hans-*LanM(R100K) was purified using the same procedure, yielding 30 mg of protein per litre of culture.

### Circular dichroism spectroscopy

Circular dichroism spectra of *Hans*-LanM were collected as described previously^32^, at 15 µM (monomer concentration) in Chelex 100-treated buffer A (20 mM acetate, 100 mM KCl, pH 5.0), unless otherwise indicated. Buffered metal solutions were prepared as described previously^4,23,25,32^. Additional details are available in the Supplementary Information.

### Preparation of protein samples for SEC–MALS and small-angle X-ray scattering (SAXS)

Samples of wild-type *Hans*-LanM were prepared by adding 3.0 equivalents of metal slowly (0.5 equivalent at a time followed by mixing) to 1.0 ml of concentrated stock of *Hans*-LanM (550 μM). At these protein concentrations, slight precipitation was observed for LRE samples (for example, La^III^) whereas significant precipitation was observed for HRE samples (for example, Dy^III^). Samples were centrifuged at 12,000*g* for 2 min to remove precipitate and then purified using gel filtration chromatography (HiLoad 10/300 Superdex 75 pg, 1-ml loop, 0.8 ml min^−1^) in buffer B (30 mM MOPS, 100 mM KCl, 5% glycerol, pH 7.0). *Hans*-LanM-containing peaks (ranging from 12.0 to 15.0 ml elution volume) were collected to avoid the high-MW aggregate peaks, yielding 2.0 ml of metalated *Hans-*LanM ranging between 114 μM and 128 μM (1.37–1.53 mg ml^−1^).

Samples of *Hans-*LanM(R100K) do not form high-MW species or precipitate on metal addition. To prepare samples of this protein, a 500 μM protein solution was diluted to 250 μM (3 mg ml^−1^) in buffer B containing 0.75 mM of a specific RECl_3_, yielding a final solution of 3 mg ml^−1^ protein, with a 1:3 metal ratio, which was analysed directly by SEC–MALS.

For calcium conditions, proteins were diluted to 250 μM (3 mg ml^−1^), 5 mM CaCl_2_ was added, and the samples were incubated at room temperature for 1 h. The buffer used for SEC–MALS was the same as above, except that it also contained 5 mM CaCl_2_.

### In-line SEC and MALS

SEC–MALS experiments were conducted using an Agilent 1260 Infinity II HPLC system equipped with an autosampler and fraction collector, and the Wyatt SEC hydrophilic column had 5-µm silica beads, a pore size of 100 Å and dimensions of 7.8 × 300 mm. Wyatt Technology DAWN MALS and Wyatt Optilab refractive index detectors were used for analysing the molar mass of peaks that eluted from the column. The SEC–MALS system was equilibrated for 5 h with buffer B. The system was calibrated with bovine serum albumin (monomer MW: 66 kDa) in the same buffer and normalization and alignment of the MALS and refractive index detectors were carried out. A volume of 15 µl of each sample was injected at a flow rate of 0.8 ml min^−1^ with a chromatogram run time of 25 min. Data were analysed using the ASTRA software (Wyatt). When small-angle X-ray scattering (SAXS) analysis was desired, a second run was carried out with 150 µl protein (about 4 mg ml^−1^) injected, and 200-µl fractions of the main peak were collected. BioSAXS data were subsequently collected in triplicate.

### Isothermal titration calorimetry

The dissociation constants for the dimers of apo, La^III^-bound and Dy^III^-bound *Hans*-LanM were determined by dilutive additions of a concentrated protein stock, followed using isothermal titration calorimetry on a TA Instruments Low-volume Auto Affinity isothermal titration calorimeter. The syringe contained 300 μM protein (apo or 2 equivalents of Dy^III^ bound) or 150 µM or 540 µM (2 equivalents of La^III^ bound), and the cell contained 185 μl of a matched buffer (30 mM MOPS, 100 mM KCl, pH 7.0). Titrations were carried out at 30 °C. Titrations consisted of a first 0.2-μl injection followed by 17 × 2-μl injections, unless otherwise noted, with stirring at 125 r.p.m. and 180 s equilibration time between injections. The data were fitted using NanoAnalyze using the Dimer Dissociation model, yielding the dimer dissociation constant (*K*_dimer_), enthalpy of dissociation (Δ*H*) and entropy of dissociation (Δ*S*). All parameters are shown in Extended Data Table 2.

*K*_dimer_ is defined as the dissociation constant for the equilibrium *D* $\rightleftharpoons$ 2*M*, such that *K*_dimer_ = [*M*]^2^/[*D*], in which [*D*] is the concentration of the dimer and [*M*] is the concentration of the monomer, and the total protein concentration [*P*] (as measured using the extinction coefficient for the monomer) is given by [*P*] = [*M*] + 2[*D*]. Therefore, *K*_dimer_ = 2[*M*]^2^/([*P*] − [*M*]) or

1 $$2{[M]}^{2}+{K}_{{\rm{dimer}}}[M]-{K}_{{\rm{dimer}}}[P]=0$$

This equation can be used to estimate monomer and dimer concentrations during SEC–MALS experiments, using *K*_dimer_ values calculated from isothermal titration calorimetry experiments and [*P*] from the SEC–MALS trace. This equation can also be used to estimate the maximum possible *K*_dimer_ for La^III^-bound protein, given the SEC–MALS data.

### SAXS

SAXS data were collected on RE-complexed *Hans*-LanM, at protein concentrations given in Supplementary Table 5 using equipment and under conditions described in the Supplementary Methods.

The forward scattering *I*(0) and the radius of gyration (*R*_g_) are listed in Supplementary Table 5 and were calculated using the Guinier approximation, which assumes that at very small angles (*q* < 1.3/*R*_g_) the intensity is approximated as *I*(*q*) =  *I*(0)exp[−1/3(*qR*_g_)^2^]. In the La^III^-, Nd^III^- and Dy^III^-bound conditions, this agrees with the calculated size of 17.9 Å for the crystallographic dimer. The molecular mass was estimated using a comparison with SAXS data of a bovine serum albumin standard. The data files were analysed for Guinier *R*_g_, maximum particle dimension (*D*_max_), Guinier fits, Kratky plots and pair-distance distribution function using the ATSAS software^52^. GNOM, within ATSAS, was used to calculate the pair-distance distribution function *P*(*r*), from which *R*_g_ and *D*_max_were determined. Solvent envelopes were computed using DENSS^36^. The theoretical scattering profiles of the constructed models were calculated and fitted to experimental scattering data using CRYSOL^53^. OLIGOMER^54^ was used to estimate the monomer and dimer fractions.

### Preparation of protein samples for crystallography

To *Hans-*LanM (2 ml, 1.16 mM, buffer B), 3.0 equivalents of LaCl_3_ or DyCl_3_ were added slowly, 0.5 equivalents at a time with mixing, to minimize precipitation. Precipitate was removed by centrifugation at 12,000*g* for 2 min. Any soluble aggregates were removed and the protein was exchanged into buffer lacking glycerol (buffer C: 30 mM MOPS, 50 mM KCl, pH 7.0) by gel filtration chromatography (HiLoad 16/600 Superdex 75 pg, 1-ml loop, 0.75 ml min^−1^). The peak in the 70–85 ml range was pooled, and the fractions were concentrated to about 500 μl with a final concentration of about 1.3 mM.

*Mex*-LanM was purified as described previously^50^ and was exchanged into buffer C before crystallization. The protein was loaded with 3.5 equivalents of Nd^III^ (NdCl_3_).

### General crystallographic methods

Diffraction datasets were collected at the Life Sciences Collaborative Access Team ID-G beamline and processed with the HKL2000 package^55^. In all structures, phase information was obtained with phenix.autosol^56,57^ through the single-wavelength anomalous diffraction method, in which lanthanide ions identified with HySS^58^ were used as the anomalous scatterers. Initial models were generated with phenix.autobuild^59^ with subsequent rounds of manual modification and refinement in Coot^60^ and phenix.refine^61^. In the final stages of model refinement, anisotropic displacement parameters and occupancies were refined for all lanthanide sites^62^. Model validation was carried out with the Molprobity server^63^. Figures were prepared using the PyMOL molecular graphics software package (Schrödinger, LLC).

### La-bound *Hans*-LanM structure determination

Crystals were obtained by using the sitting drop vapour diffusion method, in which 1 μl of protein solution (15 mg ml^−1^) was mixed with 1 μl 10 mM tri-sodium citrate, pH 7.0, and 27% (w/v) PEG 6000 in a 24-well plate from Hampton Research (catalogue number HR1-002) at room temperature. Thin plate-shaped crystals appeared in 3 days. Crystals suitable for data collection were mounted on rayon loops, soaked briefly in a cryoprotectant solution consisting of the well solution supplemented with 10% ethylene glycol, and flash-frozen in liquid N_2_.

La^III^-loaded *Hans-*LanM crystallized in the *P*2_1_ space group (*β* = 90.024°) with four monomers in the asymmetric unit. The initial figure of merit and Bayesian correlation coefficient were 0.563 and 0.56, respectively^64^. The final model consists of residues 24–133 in each chain, 12 La^III^ ions (3 per chain in the first, second and third EF hands), 4 Na^I^ ions^65^ (1 per chain in the fourth EF hand), 273 water molecules and 2 molecules of citrate. Of the residues modelled, 100% are in allowed or preferred regions as indicated by Ramachandran statistical analysis.

### Dy-bound *Hans-*LanM structure determination

Crystals were obtained by using the sitting drop vapour diffusion method, in which 1 μl of protein solution (15 mg ml^−1^) was mixed with 1 μl of 250 μM tri-sodium citrate, pH 7.0, and 27% (w/v) PEG 6000 in a 24-well plate from Hampton Research at room temperature. Thin plate-shaped crystals appeared within 1 month. Crystals suitable for data collection were mounted on rayon loops, soaked briefly in a cryoprotectant solution consisting of the well solution supplemented with perfluoropolyether cryo oil from Hampton Research (catalogue number HR2-814) and flash-frozen in liquid N_2_.

Dy^III^-loaded *Hans-*LanM crystallized in the *P*2_1_ space group (*β* = 93.567°) with four monomers in the asymmetric unit. The initial figure of merit and Bayesian correlation coefficient were 0.748 and 0.58, respectively^64^. The final model consists of residues 24–133 in each chain (except for chain D, for which residues 34–38 cannot be modelled), 14 Dy^III^ ions (4 in chains A and D, 3 in the second, third and fourth EF hands of chains B and C) and 656 water molecules. Of the residues modelled, 100% are in allowed or preferred regions as indicated by Ramachandran statistical analysis.

Collection of anomalous datasets is described in the Supplementary Methods.

### Nd-bound *Mex*-LanM structure determination

Crystals were obtained by using the sitting drop vapor diffusion method, in which 1 μl of protein solution (35 mg ml^−1^) was mixed with 1 μl of 0.1 M ammonium sulfate, 0.1 M Tris pH 7.5, and 20% (w/v) PEG 1500 in a 24-well plate from Hampton Research at room temperature. Thin plate-shaped crystals appeared within 6 months. Crystals suitable for data collection were mounted on rayon loops, soaked briefly in a cryoprotectant solution consisting of the well solution supplemented with perfluoropolyether cryo oil from Hampton Research and flash-frozen in liquid N_2_.

Nd^III^-loaded *Mex-*LanM crystallized in the *P*2_1_2_1_2_1_ space group with one monomer in the asymmetric unit. The initial figure of merit and Bayesian correlation coefficient were 0.799 and 0.56, respectively^64^. The final model consists of residues 29–133, 4 Nd^III^ ions and 171 water molecules. Of the residues modelled, 100% are in allowed or preferred regions as indicated by Ramachandran statistical analysis.

### Fluorescence spectroscopy

All fluorescence data were collected using a Fluorolog-QM fluorometer in configuration 75-21-C (Horiba Scientific) equipped with a double monochromator on the excitation arm and single monochromator on the emission arm. A 75-W xenon lamp was used as the light source for steady-state measurements and a pulsed xenon lamp was used for time-resolved measurements. Ten-millimetre quartz spectrofluorometry cuvettes (Starna Cells, 18F-Q-10-GL14-S) were used to collect data at 90° relative to the excitation path.

Fluorescence lifetime measurements were carried out using established methods^26,66^. In short, a solution of *Hans-*LanM with 2 equivalents of Eu^III^ added, totalling 4.5 ml, was prepared in 100% H_2_O matrix (buffer: 25 mM HEPES, 75 mM KCl, pH 7.0). Half of this initial protein mixture (2.25 ml) was retained for future use and the remainder was exchanged to D_2_O through lyophilization to remove H_2_O and resuspension in 99.9% D_2_O two times. The resulting protein solutions (in 100% H_2_O and about 99% D_2_O) were mixed in varying ratios to produce D_2_O contents of 0%, 25%, 50% and 75%. The protein concentration was 20 µM. For each sample, the luminescence decay time constant (*τ*) was measured (*λ*_ex_ = 394 nm, *λ*_em_ = 615 nm) with 5,000 shots over a time span of 2,500 μs. *τ* was determined using the FelixFL Powerfit-10 software (Horiba Scientific) using a single exponential fit. 1/*τ* was plotted against percentage composition of D_2_O, and the slope of the resulting line (*m*) was determined. The *q* value was determined using the following equation from ref. ^67^:

2 $$q=1.11[{\tau }_{{\rm{H}}2{\rm{O}}}^{-1}-{\tau }_{{\rm{D}}2{\rm{O}}}^{-1}-0.31+0.45{n}_{{\rm{OH}}}+0.99{n}_{{\rm{NH}}}+0.075{n}_{{\rm{O}}\mbox{--}{\rm{CNH}}}]$$

in which *τ*^−1^_H2O_ and *τ*^−1^_D2O_ are the inverses of the time constants in 100% H_2_O and D_2_O, respectively (the latter extrapolated using the equation of the fitted line), in ms^–1^; and *n*_OH_ = 0, *n*_NH_ = 0, and *n*_O–CNH_ = 1 (resulting from the metal-coordinated Asn residues), on the basis of the *Hans*-LanM crystal structures. This equation simplifies to:

3 $$q=1.11[-m-0.31+0.075]$$

For fluorescence competition experiments, a solution of 20 μM *Hans*-LanM or the R100K variant was prepared in buffer A (pH 5.0) with two equivalents of metal (40 μM). Fluorescence emission spectra were collected with settings: *λ*_ex_ = 278 nm, *λ*_em_ 300–420 nm, integration time = 0.5 s, step size = 1 nm. Titrations were carried out through addition of at least 0.6 μl of titrant (from concentrated stock solutions of 10 mM–1 M citrate or malonate, pH 5.0). Spectra were corrected for dilution. Each experiment was carried out in triplicate.

### Purification of Cys-containing variants

*Hans*-LanM(R100K)-Cys was expressed and purified as described for *Mex-*LanM-Cys (ref. ^6^), with a final yield of 50 mg of protein per litre of culture. For *Hans*-LanM-Cys, the protein was purified by incorporating the same modifications from above, minus the dialysis step, to our previously described *Mex*-LanM-Cys purification, except that the SEC step was run using a reducing buffer (30 mM MOPS, 100 mM KCl, 5 mM TCEP, pH 7.0) with 5 mM EDTA, and frozen under liquid N_2_ before immobilization.

### Maleimide functionalization of agarose beads

The maleimide functionalization of amine-functionalized agarose beads was described previously^6^. See the Supplementary Information for complete details.

### Immobilization of *Hans*-LanM and the R100K variant

*Hans*-LanM(R100K) immobilization was carried out using a thiol-maleimide conjugation reaction as described previously^6^. In the case of *Hans*-LanM, a final protein concentration of about 0.4 mM (8 ml) was combined with 1 ml of maleimide–microbeads and the conjugation reaction was carried out for 16 h at room temperature. Unconjugated *Hans*-LanM was removed by washing with coupling buffer, and the *Hans-*LanM microbeads were stored in coupling buffer for subsequent tests. To quantify *Hans*-LanM immobilization yield, Pierce BCA Protein Assay (ThermoFisher Scientific) was used to determine the LanM concentration in the reaction solution before and after the conjugation reaction as previously described.

### Batch experiment to determine separation factors

LanM-immobilized microbeads were washed with deionized water. Feed solution (5 ml, equimolar REs La–Dy, 3 mM total, pH 5.0) was added to 1 ml microbeads and incubated for 2 h. The liquid at equilibrium was collected and RE concentrations were determined by inductively coupled plasma mass spectrometry as [*M*]_ad_. Then 4 ml of 0.1 M HCl was used to desorb REs from the microbeads and concentrations were measured by inductively coupled plasma mass spectrometry as [*M*]_de_.

The RE distribution factor (*D*) between the LanM phase and the solution phase was calculated as:

4 $$D=\frac{{[M]}_{{\rm{LanM}}}}{{[M]}_{{\rm{Liquid}}}}$$

in which [*M*]_LanM_ and [*M*]_Liquid_ are the molar concentrations of each metal ion in the LanM phase and the solution phase at equilibrium, respectively. To account for the free liquid that absorbed on the agarose microbeads, the following correction was applied: [*M*]_Liquid_ = [*M*]_ad_; [*M*]_LanM_ = (4 × [*M*]_de_ – [*M*]_ad_)/4.

The separation factor is defined as:

5 $${\rm{SF}}=\frac{{D}_{{\rm{RE1}}}}{{D}_{{\rm{RE2}}}}$$

in which *D*_RE1_ and *D*_RE2_ are the distribution factors of RE1 and RE2, respectively.

### Breakthrough column experiments

Columns were filled and run, and metal concentrations analysed, as described in our previous work^6^; details are available in the Supplementary Methods.

For the RE pair separation experiments, the metal ion purity and yield are defined as:

6 $${{\rm{Purity}}}_{{\rm{RE}}1}=\frac{{C}_{{\rm{RE}}1}}{{C}_{{\rm{RE}}1}+{C}_{{\rm{RE}}2}}$$

7 $${{\rm{Y}}{\rm{i}}{\rm{e}}{\rm{l}}{\rm{d}}}_{{\rm{R}}{\rm{E}}1}=\frac{{\rm{R}}{\rm{E}}1\,{\rm{r}}{\rm{e}}{\rm{c}}{\rm{o}}{\rm{v}}{\rm{e}}{\rm{r}}{\rm{e}}{\rm{d}}}{{\rm{T}}{\rm{o}}{\rm{t}}{\rm{a}}{\rm{l}}\,{\rm{R}}{\rm{E}}1\,{\rm{l}}{\rm{o}}{\rm{a}}{\rm{d}}{\rm{e}}{\rm{d}}}$$

in which *C*_RE1_ and *C*_RE2_ are the molar concentrations of RE1 and RE2, respectively.

### Reporting summary

Further information on research design is available in the Nature Portfolio Reporting Summary linked to this article.

## Online content

Any methods, additional references, Nature Portfolio reporting summaries, source data, extended data, supplementary information, acknowledgements, peer review information; details of author contributions and competing interests; and statements of data and code availability are available at 10.1038/s41586-023-05945-5.

### Supplementary information

Supplementary Information This file contains Supplementary Methods, Supplementary Figs 1-31, Supplementary Tables 1-16 and Supplementary References.

Reporting Summary

Source Data for Supplementary Fig. 3

Source Data for Supplementary Fig. 10

Source Data for Supplementary Fig. 19

Source Data for Supplementary Fig. 21

Source Data for Supplementary Fig. 30

Source Data for Supplementary Table 12

Source Data for Supplementary Table 13

Source Data for Supplementary Table 14

### Source data

Source Data Fig. 1

Source Data Fig. 4

Source Data Extended Data Fig. 2

Source Data Extended Data Fig. 5

Source Data Extended Data Fig. 6

Source Data Extended Data Table 3

## Figures and Tables

**Extended Data Table 1:**
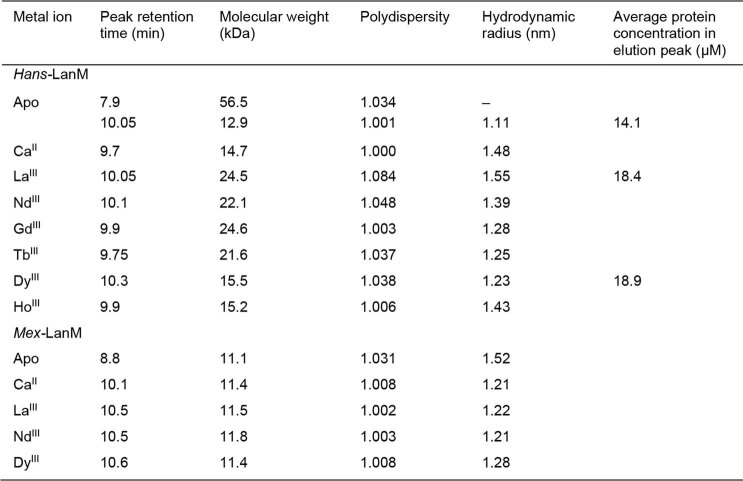
Characterization of *Hans-* and *Mex*-LanM metal complexes using SEC-MALS

The concentrations of the protein samples loaded to the column were: 1.2—1.5 mg/mL for apo- and RE-bound *Hans*-LanM, 3 mg/mL for Ca^II^-bound *Hans*-LanM, and 3 mg/mL for *Mex*-LanM. For RE-containing samples, protein was pre-incubated with 3 equiv. of the appropriate RE^III^ ion. In the case of Ca^II^, 5 mM CaCl_2_ was added to the running buffer. The apoprotein elutes in two peaks, the first being a minor contribution (10% of protein, 56.5 kDa) and the second being the major peak (90% of protein, 12.9 kDa). See Materials and Methods for full details of sample preparation. The values for *Hans*-LanM are plotted in Fig. 2a; raw data for La, Nd, and Dy are shown in Supplementary Fig. 5.

**Extended Data Table 2:**
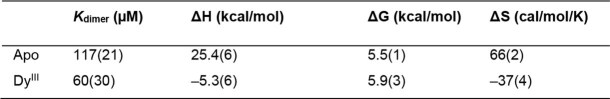
Thermodynamic parameters for apo- and Dy^III^-bound *Hans*-LanM, obtained by fitting ITC thermograms to the dimer dissociation model

These values cannot be determined for the La^III^-bound protein because no changes in the measured heats are observed during the titration experiment (Supplementary Fig. 8). Values are reported as the mean with standard deviation from three independent titrations.

**Extended Data Table 3:**
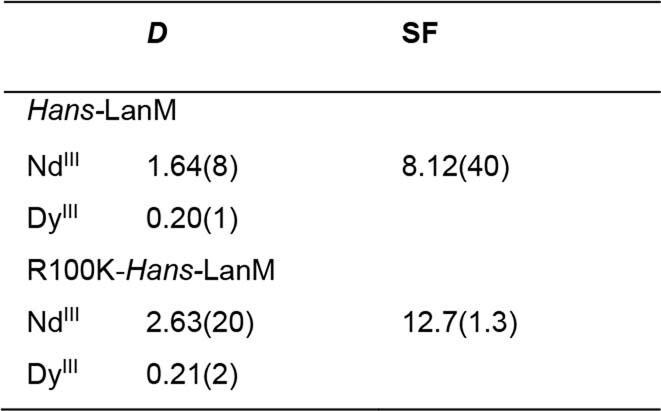
Distribution factors (*D*) and separation factors (SF) for equilibration of a binary Nd/Dy solution using a *Hans*-LanM column or *Hans*-LanM(R100K) column

The volumes of the *Hans*-LanM column and *Hans*-LanM(R100K) column were 0.9 mL and 0.7 mL, respectively. The feed solution for this experiment was 5.0 mL with a composition of 1.42(4) mM Nd and 1.62(32) mM Dy, as determined by ICP-MS analysis. The pH was 5.0. This experiment confirms that the immobilized R100K variant exhibits better on-column separation properties than wild-type *Hans*-LanM. See Supplementary Table 12 legend for details on uncertainty values in parentheses.

Source Data

## Data Availability

All data are available in the main text or the Supplementary Information. Coordinates have been deposited in the Protein Data Bank with accession codes 8DQ2 (La^III^–*Hans*-LanM), 8FNR (Dy^II^–*Hans*-LanM) and 8FNS (Nd^III^–*Mex*-LanM). Source data are provided with this paper.
