# Supplementary material for: Enhanced rare-earth separation with a metal-sensitive lanmodulin dimer
Source: Nature. 2023 May 31;618(7963):87–93. doi: 10.1038/s41586-023-05945-5 (PMC10232371; doi:10.1038/s41586-023-05945-5)
Supplement: Supplementary file 1 — This file contains Supplementary Methods, Supplementary Figs 1-31, Supplementary Tables 1-16 and Supplementary References. [file 41586_2023_5945_MOESM1_ESM.pdf]

nature portfolio

https://doi.org/10.1038/s41586-023-05945-5

Supplementary information

Enhanced rare-earth separation with a metal-sensitive lanmodulin dimer

In the format provided by the authors and unedited

Supplementary Information for

Enhanced rare-earth separation with a metal-sensitive lanmodulin dimer

Joseph A. Mattocks,[1] Jonathan J. Jung,[1] Chi-Yun Lin,[1] Ziye Dong,[2] Neela H. Yennawar,[3] Emily R. Featherston,[1] Christina S. Kang-Yun,[2] Timothy A. Hamilton,[1] Dan M. Park,[2]* Amie K. Boal,[1,4]* and Joseph A. Cotruvo, Jr.[1]*

[1]Department of Chemistry, The Pennsylvania State University; University Park, Pennsylvania 16802, United States

[2]Critical Materials Institute, Physical and Life Sciences Directorate, Lawrence Livermore National Laboratory; Livermore, California 94550, United States

[3]The Huck Institutes of the Life Sciences, The Pennsylvania State University; University Park, Pennsylvania 16802, United States

[4]Department of Biochemistry and Molecular Biology, The Pennsylvania State University; University Park, Pennsylvania 16802, United States

*Corresponding author. Email: juc96@psu.edu, akb20@psu.edu, park36@llnl.gov

SUPPLEMENTARY METHODS

General considerations. Chemical reagents were obtained, at the highest purity available, from Millipore Sigma, unless noted otherwise. Chemically competent *E. coli* BL21 (DE3) cells were obtained from NEB. Biochemical experiments and column-based experiments were performed using RE chloride salts and buffers obtained at a minimum purity of 99.9% from Millipore Sigma. Anion exchange chromatography was performed using Q Sepharose Fast Flow resin obtained from Millipore Sigma. Automated protein chromatography was carried out on a GE Healthcare Biosciences Akta Pure fast protein liquid chromatography (FPLC) system using either a HiLoad Superdex 75 pg 16/600 column for preparative scale or a Superdex 75 pg Increase 10/300 GL column for analytical scale. REs were quantified using inductively coupled plasma mass spectrometry (ICP-MS; Thermo Scientific iCAP RQ) with He in KED mode. The ICP-MS is housed in the Laboratory for Isotopes and Metals in the Environment (LIME), in the Earth and Environmental Systems Institute at the Pennsylvania State University. For protein immobilization, amine-functionalized agarose beads were purchased from Nanocs Inc. *N*-Succinimidyl 4-(maleimidomethyl) cyclohexane-1-carboxylate (SMCC) was purchased from Chem-Impex International, Inc. and used without further purification.

Circular dichroism (CD) spectroscopy. CD spectra of *Hans*-LanM were collected as described.[1] In short, a Jasco J-1500 CD spectrometer was used to scan samples from 195 to 255 nm using the following settings: 1 nm bandwidth, 0.5 nm data pitch, 50 nm/min scan rate, 4 s average time. For all buffered metal and stoichiometric titrations, the cuvette contained 15 μM protein (monomer concentration). For stoichiometric titrations, the protein was diluted into Chelex-treated **Buffer A** (20 mM acetate, 100 mM KCl, pH 5.0) and titrated with 0.5-4.0 equiv. of each metal ion from a 1.5 mM solution in the same buffer.

Buffered metal solutions were prepared as described.[1-4] EDTA was used as the chelator for titrations with Ca^{II}, La^{III}, and Nd^{III}; EGTA was used for Dy^{III}. Protein was added to 15 μM in the respective "high" and "low" metal solutions for each metal ion. These solutions were then combined to a final volume of 200 μL, in various ratios, to produce a range of free metal concentrations in the presence of *Hans*-LanM. These solutions were incubated overnight at 4° C. CD spectra were collected and the CD signal at 222 nm was plotted against the free metal concentration, yielding binding curves that were fitted using the Hill equation.

BioSAXS data acquisition. Data were collected at a wavelength of 1.54 Å on the home source at the Penn State X-Ray Crystallography Facility, with X-rays generated by a Rigaku MM007 rotating anode housed with the BioSAXS2000^{nano} Kratky camera system. The system includes OptiSAXS confocal max-flux optics that are designed specifically for SAXS and a HyPix-3000 Hybrid Photon Counting detector. The sample capillary-to-detector distance was 495.5 mm and was calibrated using silver behenate powder (The Gem Dugout, State College, PA). The useful *q*-space range (4πsinθ/λ, where 2θ is the scattering angle) was generally from $q_{min} = 0.008$ Å^{-1} to $q_{max} = 0.3$ Å^{-1}. The energy of the X-ray beam was 1.2 keV, with the Kratky block attenuation of 22% and a beam diameter of ~100 μm.

Protein samples were loaded using the autosampler onto a quartz capillary flow cell, mounted on a stage maintained at 22 °C and aligned in the X-ray beam. The sample cell and full X-ray flight path, including beam stop, were kept in vacuo (< 1×10^{-3} torr) to eliminate air scatter. The Rigaku SAXSLAB software was programmed for automated data collection of each protein

sample and matched buffers, with rigorous cleaning with 1 M NaOH prior to the start of the run and with water and ethanol between samples. Data reduction, including image integration and normalization, and background buffer data subtraction were also carried out using the SAXSLAB software. Six 10-min images from protein and buffer samples were collected and averaged after ensuring that no X-ray radiation damage had occurred. SAXS data overlays showed that there was no radiation decay over the 60 min of data collection. This was followed by reference buffer subtraction to obtain the raw SAXS scattering curve from only the protein.

Dy-bound *Hans*-LanM structure determination. Anomalous scattering datasets were collected at National Cancer Institute Structural Biology Facility (GM/CA) beamline ID-D at the Advanced Photon Source (Argonne National Laboratory, Argonne, IL). The X-ray absorption spectrum was first measured (**Fig. S25**), and two energies, 7793.5 (L_{III} edge) and 7760 (pre-edge) eV, were chosen accordingly to maximize the differences in absorption (f''). The diffraction data at the two energies were collected alternately using inverse-beam geometry with 30-degree wedges to minimize radiation damage and subsequently processed with HKL2000.

The coordinates determined from high-resolution data were further refined against the new dataset collected at 7793.5 eV using phenix.refine to account for batch variations. As opposed to the high-resolution structure, no heavy element can be found in EF1 of chain D, while the remaining 13 dysprosium ions were retained. No other visible structural changes were observed. The anomalous difference map was prepared with phenix.maps, and the corresponding peak intensities (**Table S10**) were inspected via Coot. The large differences in anomalous peak intensities determined at the pre-edge and on-edge energies corresponding to Dy strongly indicate that the metals bound at EF hands are indeed Dy.

Maleimide functionalization of agarose beads. The maleimide functionalization of amine-functionalized agarose beads was described previously.[5] Briefly, agarose microbeads (1.2 mL) were aliquoted into 5 mL Eppendorf tubes and preconditioned with pH 7.4 phosphate-buffered saline (PBS) and resuspended at a final volume of ~1.7 mL (1.2 mL microbeads and 0.5 mL PBS supernatant). SMCC (0.15 g) powder was dissolved in 3.4 mL DMSO and combined with the microbeads. After 2.5 h incubation on a rocker mixer at room temperature, the modified agarose microbeads were washed with DMSO three times to remove unreacted SMCC and then washed with coupling buffer (50 mM HEPES, 50 mM KCl, pH 7.0) three times to remove DMSO. The maleimide-microbeads were then used for LanM immobilization within 2 h.

Breakthrough column experiments. Econo-Column glass chromatography columns (Bio-Rad; 5 cm × 0.5 cm) were filled with MilliQ water (18.2 MΩ cm^{-1}) and LanM-microbeads were added gravimetrically. Columns were washed with 25 mM HCl, MilliQ water, and conditioned with **Buffer D** (10 mM homopiperazine-1,4-bis(2-ethanesulfonic acid), pH 5.0) before conducting breakthrough experiments. RE stock solutions were prepared by dissolving individual RE chloride salts in 1 mM HCl. The stock solutions were diluted in Buffer D. The RE solutions were pumped at 0.5 mL/min unless otherwise specified and the column effluent was collected in 1.0 mL aliquots. A washing step with 5 bed volumes of MilliQ water was included before performing desorption experiments with the stated concentrations of chelator or HCl. For single RE ion solutions, RE ion concentrations were quantified using the Arsenazo III assay. Specifically, 40 µL of sample was combined with 40 µL of 12.5 wt.% trichloroacetic acid (TCA) and then added to 120 µL of filtered 0.1 wt.% Arsenazo in 6.25 wt.% TCA. Absorbance at 652 nm was measured and compared to

standards to determine the RE metal ion concentrations. The accuracy of the colorimetric assay was also confirmed in our previous work[5] by ICP-MS. For experiments with RE mixtures, the metal ion concentrations were determined by ICP-MS (**Extended Data Table 3, Table S16**).

SUPPLEMENTARY FIGURES

```
Mex LanM     22 APTTTTKVDIAAFDPDKDGTIDLKEALAAGSAAFDKLDPDKDGTLDAKELKGRVSEADLK 81
Hans LanM    24 ---ASGADALKALNKDNDDSLEIAEVIHAGATTFTAINPDGDTTLESGETKGRLTEKDWA 80
                   ::    : *:: *:*.:::: *.: **:::*  ::** * **:: * ***::* *

Mex LanM     82 KLDPDNDGTLDKKEYLAAVEAQFKAANPDNDGTIDARELASPAGSALVNLIR- 133
Hans LanM    81 RANKDGDQTLEMDEWLKILRTRFKRADANKDGKLTAAELDSKAGQGVLVMIMK 133
                : : *.* **: .*:*  :.::** *: ::**.: * ** * **..:: :*
```

Fig. S1. Sequence alignment of *Mex*-LanM and *Hans*-LanM (after signal peptide removal), showing 33% sequence identity. EF-hands are shown in bold. See **Table S12** for the full-length *Hans*-LanM sequence including the predicted signal peptide. The full-length *Mex*-LanM sequence is given in Cotruvo et al.[2] The *Hans*-LanM protein used in the present study consisted of residues A24-K133.

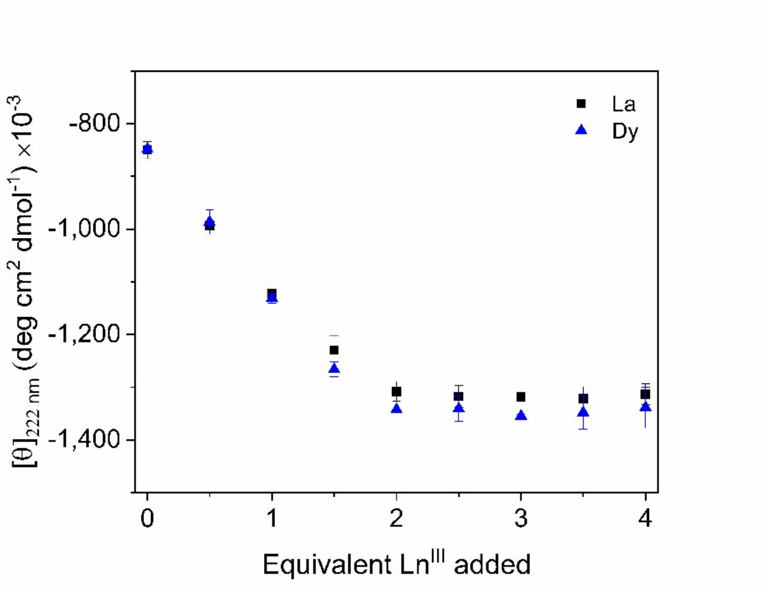

Fig. S2. Stoichiometric metal titrations of *Hans*-LanM (15 μM) with La^{III} and Dy^{III}, monitored by CD spectroscopy. Molar ellipticity at 222 nm is plotted against equivalents of metal added. Experimental conditions: 20 mM acetate, 100 mM KCl, pH 5.0. Each data point is the mean ± SD for two independent measurements. Taken together with **Fig. 1**, these observations suggest the following model for *Hans*-LanM's interactions with RE^{III} ions: tight (though preferential for LREs) metal binding to one site results in a conformational change; a second site responds to LREs cooperatively with the first to give the complete conformational response, but it responds to Dy non-cooperatively and only at >0.5 μM concentrations; and a third site does not cause an observable conformational change with any RE. We postulate that the third site is EF1 based on prior work with *Mex*-LanM and the crystal structures described herein; we cannot definitively distinguish EF2 and EF3 as the first and second sites. This more complex response profile than that of *Mex*-LanM[6] appears tuned to ensure a full, cooperative response *only* to LREs.

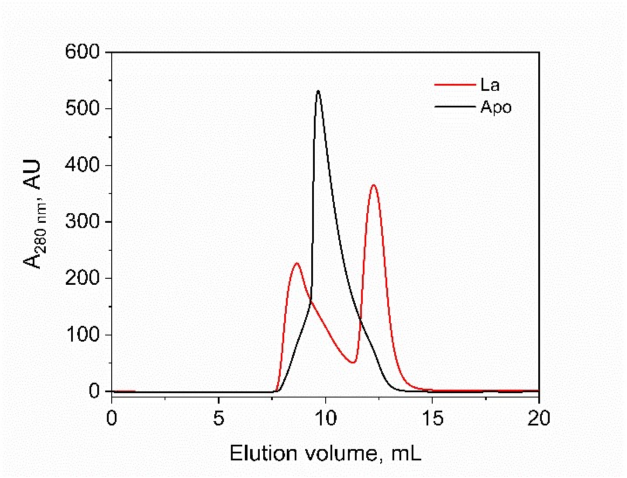

Fig. S3. Size-exclusion chromatograms of apo *Hans*-LanM (black) and *Hans*-LanM metalated with 3.0 equiv. La^{III} (red). The S75 column volume was 24 mL. The apoprotein, like apo-*Mex* LanM, elutes over a wide molecular weight range (30-70 kDa), suggestive of multiple, disordered conformations. The La^{III}-bound protein displays soluble higher molecular weight species that form when REs are added to *Hans*-LanM at high concentrations, as well as a symmetrical peak at ~28 kDa suggestive of a dimer.

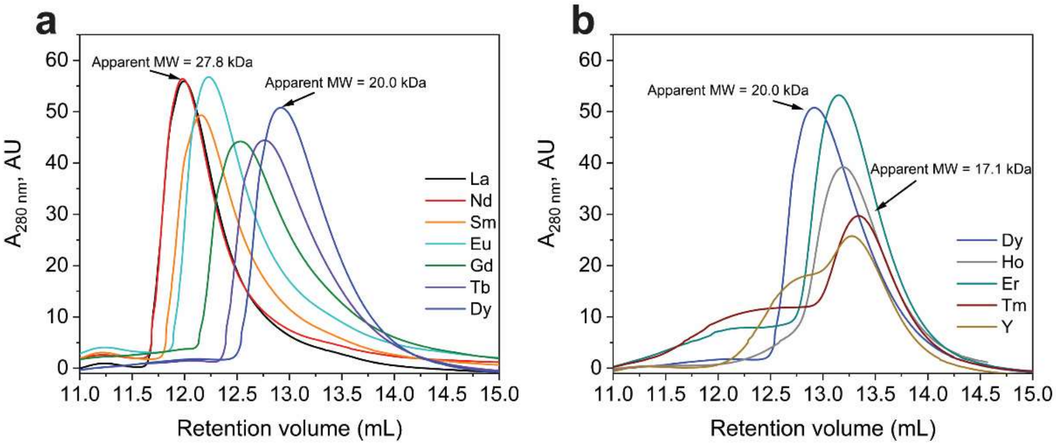

Fig. S4. Size-exclusion chromatography of RE^{III}-*Hans*-LanM complexes (RE = La, Nd, Sm, Eu, Gd, Tb, Dy, Ho, Er, Tm, Y). Apo-Hans (590 μM, 100 μL) was metalated with 3.0 equiv. RE, 0.5 equiv. at a time with mixing, and loaded to a 24 mL analytical S75 column calibrated as described.[2] **a,** Chromatograms for RE = La-Dy show an apparent molecular weight suggestive of a *Hans*-LanM dimer for La and Nd, which progressively shifts to lower apparent molecular weight, likely indicating decreasing proportion of dimer in rapid equilibrium with monomer. **b,** Beyond Dy, the speciation of the complexes is complicated but suggests emergence of a more extended monomer or a slower-exchanging dimer population, which was not probed further in the present work.

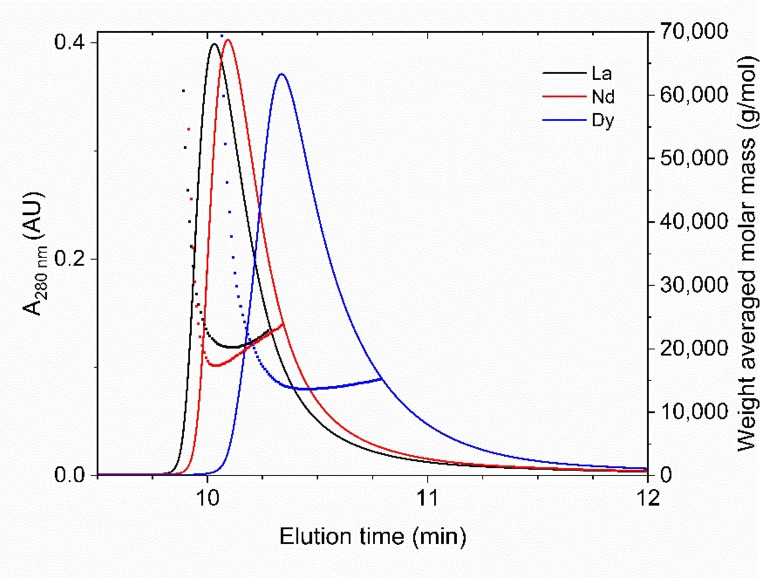

Fig. S5. SEC-MALS traces for La-, Nd-, and Dy-bound *Hans*-LanM, illustrating the later elution and lower weight-average molar mass of the Dy complex (see **Table S3**). Apo-*Hans*-LanM was incubated with 3 equiv. each RE^{III} ion, precipitate and aggregate were removed by centrifugation and analytical SEC, and the protein was injected to the column at a concentration of 114-128 μM.

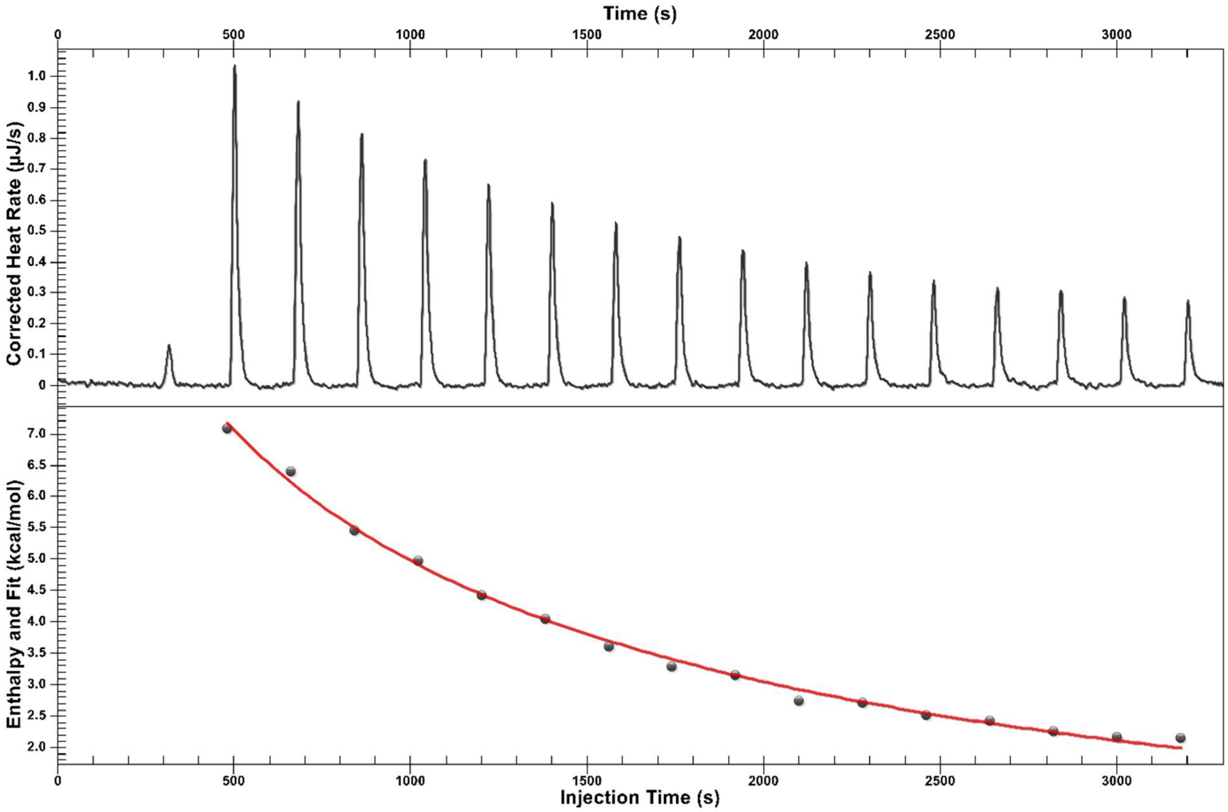

Fig. S6. Dimer dissociation of Apo *Hans*-LanM followed by ITC. (Top) Representative ITC trace for titration of 300 μM protein into buffer. (Bottom) Thermogram derived from the data above fitted to the dimer dissociation model using the NanoAnalyze software, with parameters presented in **Extended Data Table 2**. Conditions: 30 mM MOPS, 100 mM KCl, pH 7.0, 30 °C.

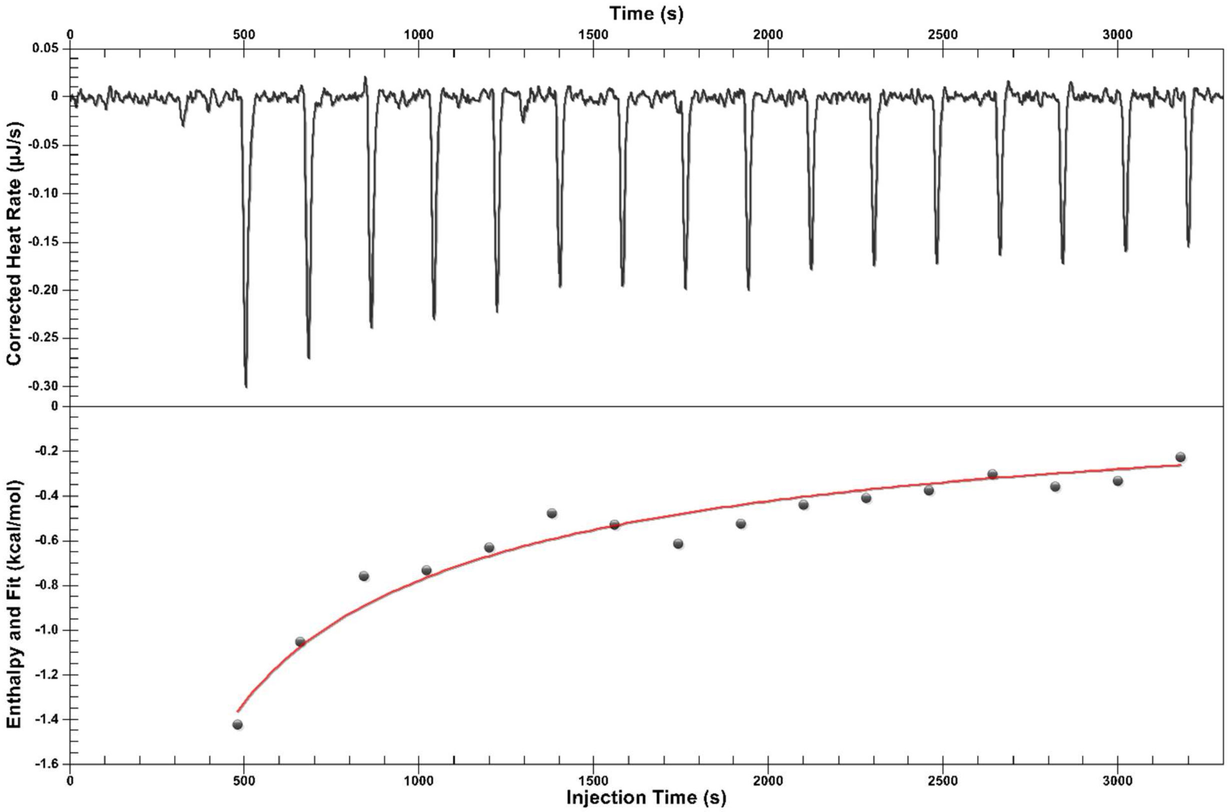

Fig. S7. Dimer dissociation of Dy^{III}_2-*Hans*-LanM followed by ITC. (Top) Representative ITC trace for titration of 300 μM protein into buffer. (Bottom) Thermogram derived from the data above fitted to the dimer dissociation model using the NanoAnalyze software, with parameters presented in **Extended Data Table 2**. Conditions: 30 mM MOPS, 100 mM KCl, pH 7.0, 30 °C.

Utilizing Eq. 1, the concentrations of Dy^{III}-*Hans*-LanM monomer and dimer under the conditions of SEC-MALS can be calculated, given [P] = 18.9 μM (obtained from **Extended Data Table 1**) and K_{dimer} = 60 μM obtained from ITC (**Extended Data Table 2**). We obtain [M] = 13.5 μM and thus [D] = 3.3 μM. Because [D] is ~25% of [M], this result corresponds well to the average mass of 15.5 kDa obtained by SEC-MALS for this form, which is ~25% higher than the expected MW of 11.9 kDa, suggesting partial dimerization. This, in turn, supports our interpretation that the SEC-MALS results indicate rapid monomer-dimer equilibria where the individual monomer and dimer components cannot be resolved, therefore manifesting as a weighted average of the two populations.

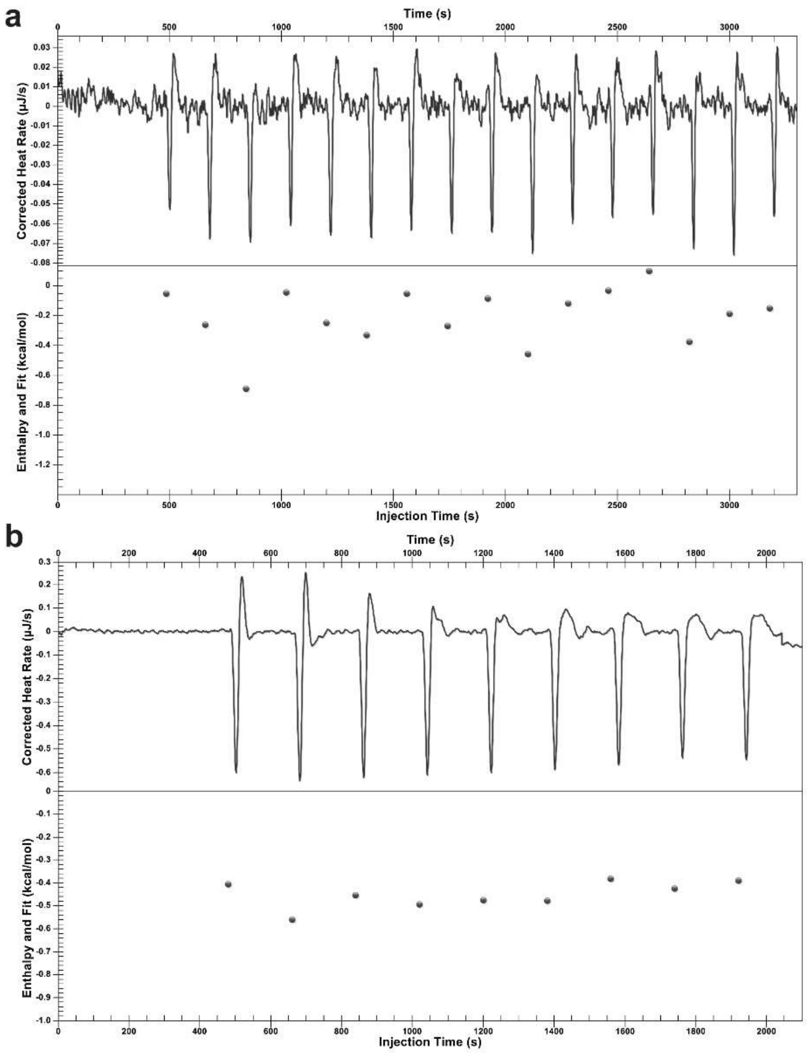

Fig. S8. Titration of La^{III}_2-*Hans* LanM followed by ITC. (**a**, Top) Representative ITC trace for titration of 150 μM protein into buffer. (**a**, Bottom) Thermogram derived from the data above. (**b**, Top) Representative ITC trace for titration of 540 μM protein into buffer with an initial 0.2 μL injection followed by 9 × 5.0 μL additions. (**b**, Bottom) Thermogram derived from the data above. The inability to observe differences between the heats of each injection in these experiments reinforces the very tight binding in the La^{III}-bound dimer. Conditions: 30 mM MOPS, 100 mM KCl, pH 7.0, 30 °C.

We can estimate the maximum dimer dissociation constant for La^{III}-*Hans*-LanM by using Eq. 1, the peak concentration from SEC-MALS, [P] = 18.4 μM (**Extended Data Table 1**), and conservatively assuming that the minimum threshold for observable monomer [M] is 10% of the total protein concentration (1.84 μM), because it would appear in the SEC-MALS as a roughly 1 kDa difference from the theoretical monomer MW of 11.9 kDa (such a difference is observable in the case of apo-*Hans*-LanM, see **Extended Data Table 1**). In this case, solving for $\boldsymbol{K_{\mathrm{dimer}}}$ **yields 0.4 μM**, which represents the maximum dissociation constant for the dimer of the La^{III}-bound protein. Therefore, *Hans*-LanM exhibits a more than 100-fold enhanced dimerization in the presence of La^{III} versus Dy^{III}.

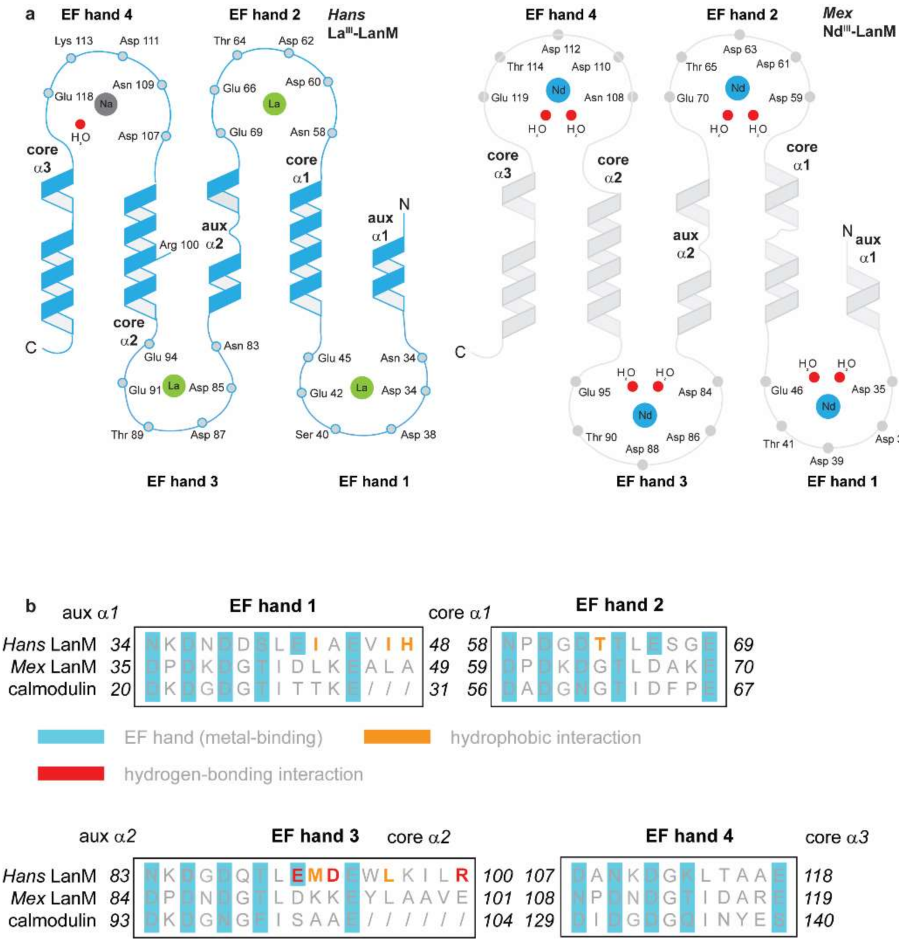

Fig. S9. Comparison of topology and key residues in *Hans*- and *Mex*-LanMs. **a**, Topology diagrams for La^{III}-*Hans*-LanM and Nd^{III}-*Mex*-LanM. Despite possessing only 33% sequence identity, the overall topologies of the two proteins are very similar, with three core helices (α1-3) forming the central three-helix bundle, and decorated with two auxiliary helices, preceding EF1 and EF3. As explained in **Fig. S28**, the presence of a Nd^{III} ion in EF4 of *Mex*-LanM is a result of the high protein concentration used for crystallization. **b,** Sequence alignment of key regions of *Hans*- and *Mex*-LanMs and *H. sapiens* calmodulin. The similarity of metal-binding residues in these proteins is highlighted in light blue; note the presence of Glu residues at the 9th position of each EF-hand in *Hans*-LanM, uniquely relative to the other proteins. The residues that are involved in interactions at the dimer interface in *Hans*-LanM are bolded in red (hydrogen bonding interactions: E91, D93, R100) and orange (hydrophobic interactions: I43, I47, H48, T63, M92, L96). The charges of residues corresponding to *Hans*-LanM D93 and R100 are reversed (K94, E101) in *Mex*-LanM. Several of the residues involved in hydrophobic interactions (I43, I47, L96) in *Hans*-LanM correspond to similar residues in *Mex*-LanM (L44, L48, L97), whereas the others (H48, T63, M92) correspond to residues that either lack bulky sidechains (A49, G64) or are charged (K94). This analysis also highlights another potentially important difference, that calmodulin possesses highly conserved Gly residues at both the 4th and 6th positions of each EF-hand; Gly residues are conserved at those positions in 81-100% of predicted calmodulin sequences,

depending on the EF-hand/residue position.[7] By contrast, *Hans*- and *Mex*-LanM EF-hands only have a glycine at one of those positions (or none, in *Hans*-LanM EF1).

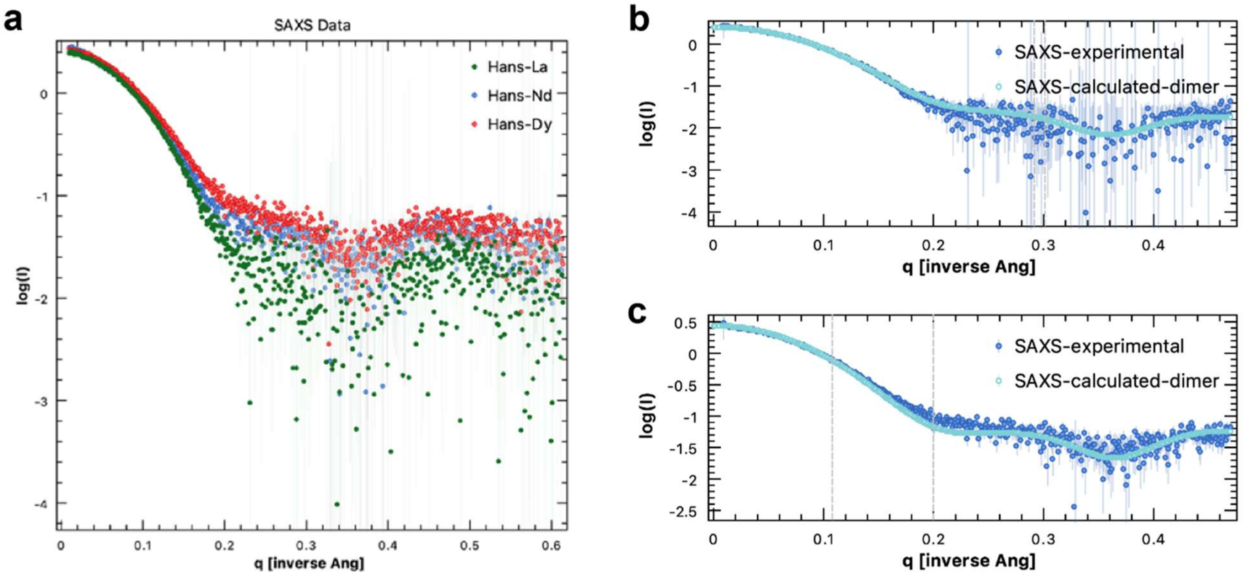

Fig. S10. SAXS raw data for La-, Nd-, and Dy-bound *Hans*-LanM. SAXS datasets were collected on SEC-MALS fractions in presence of different metal ions in 30 mM MOPS, 100 mM KCl, 5% glycerol, pH 7.0, on an in-house Rigaku BioSAXS2000^{nano}. **a,** The La-bound protein (green) was 1.5 mg/mL, the Nd-bound protein (blue) was 1.4 mg/mL, and the Dy-bound protein (red) was 1.6 mg/mL. SAXS data sets were collected for 60 min with six 10-min images using the autosampler quartz flow cell. Buffer SAXS data were collected over 60 min using the same flow cell and were used for the reference subtraction. The overlays of the six 10-min images that were averaged in each case showed that there was no X-ray radiation damage. **b,** and **c,** CRYSOL[8] (ATSAS) fits of the La and Dy SAXS data to the crystallographic La-*Hans*-LanM and Dy-*Hans*-LanM dimer models. Chi-squared values are listed in **Table S6**.

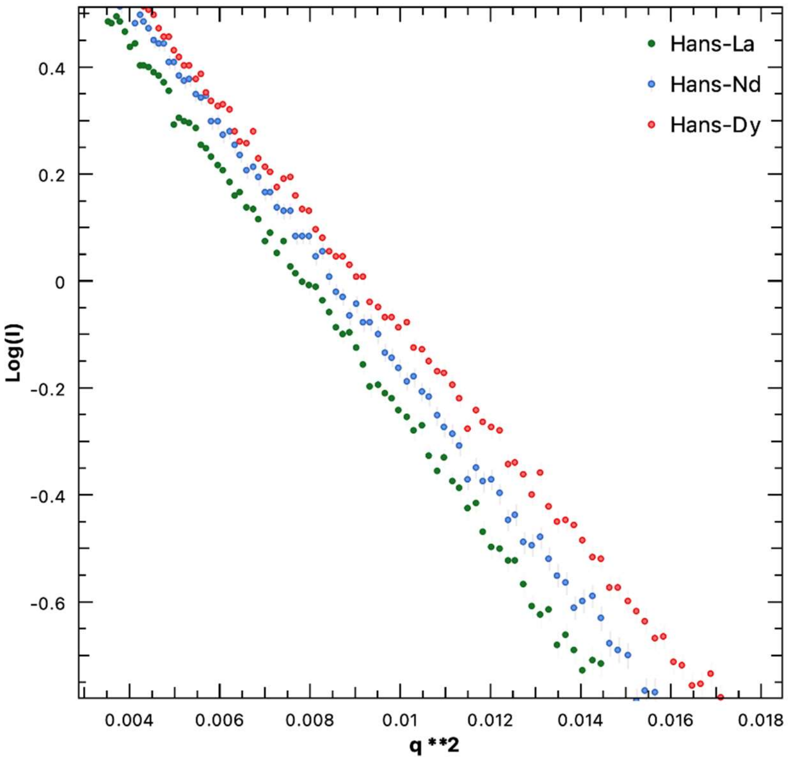

Fig. S11. Overlay of the Guinier plots for La- (green), Nd- (blue), and Dy-bound (red) *Hans*-LanM. The fits point to radii of gyration (R_g) of 18.5 ± 2.5 Å (La), 18.7 ± 2.7 Å (Nd), and 17.8 ± 2.6 Å (Dy) (see **Table S4**). Note that there is less difference between the R_g values for the Dy and the La/Nd complexes than might be expected based on SEC-MALS (**Extended Data Table 1** suggests a ~2-3 Å difference in hydrodynamic radius for La/Nd vs. Dy), because the protein concentrations used for SAXS are 5-fold higher than those for SEC-MALS, and therefore the population of Dy-bound dimer is substantially larger in the SAXS experiment. Nevertheless, these RE-dependent differences are within the uncertainty of the SAXS R_g values.

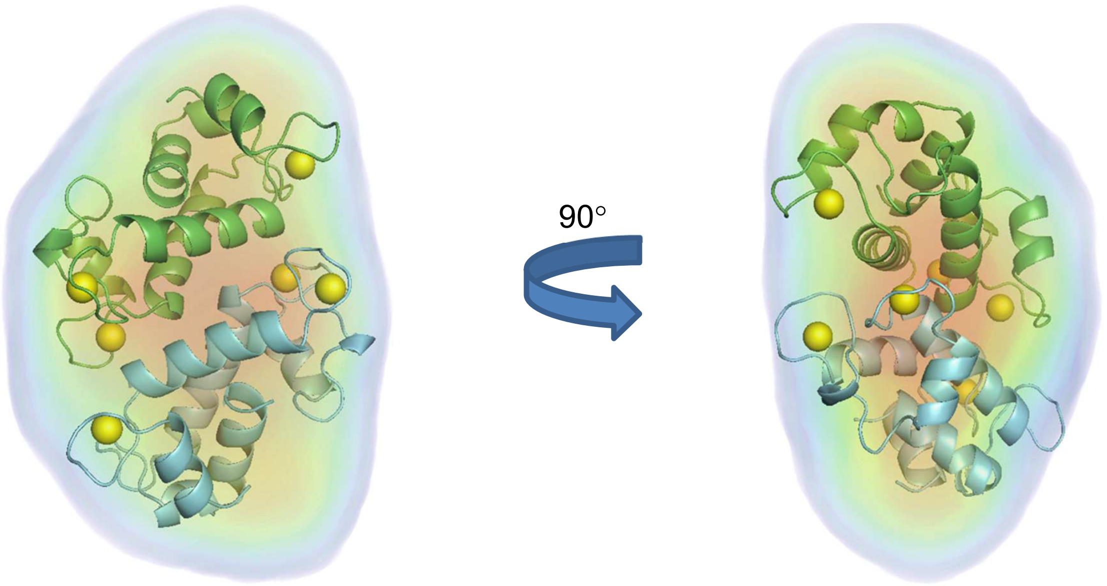

Fig. S12. La^{III}-bound *Hans*-LanM solvent envelope. DENsity from Solution Scattering (DENSS) is an algorithm for calculating ab initio electron density maps from solution scattering data.[9] The DENSS electron density map, shown as a transparent surface in the PyMOL-generated representation, overlays with the dimeric La^{III}-*Hans*-LanM crystal structure, colored here by chain (light blue and green). La^{III} ions are represented as gold spheres. The color ramp is from lowest to highest electron densities: blue (2σ) to cyan (5σ) to green (7.5σ) to yellow (10σ) to red (15σ). Manual fitting of the envelope and model was performed in PyMOL.[10] The CRYSOL fit of the calculated SAXS profile overlays with the experimental SAXS profile with a Chi-squared fit of 1.1 (**Table S6**). The left panel is identical to the La^{III}-*Hans*-LanM data shown in **Fig. 2e**.

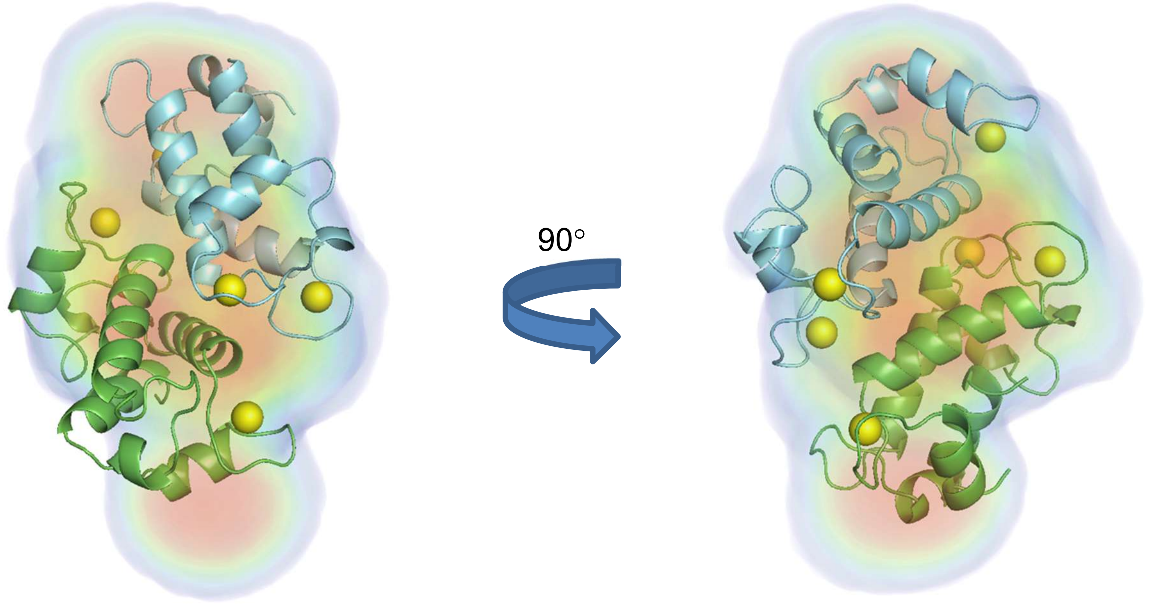

Fig. S13. Nd^{III}-bound *Hans*-LanM solvent envelope. The DENSS electron density map, shown as a transparent surface in the PyMOL-generated representation, overlays with the dimeric La^{III}-*Hans*-LanM crystal structure, colored here by chain (light blue and green) with La^{III} ions represented as gold spheres. Manual fitting of the envelope and model was performed in PyMOL. The CRYSOL fit of the calculated SAXS profile overlays with the experimental SAXS profile with a Chi-squared fit of 1.3 (**Table S6**).

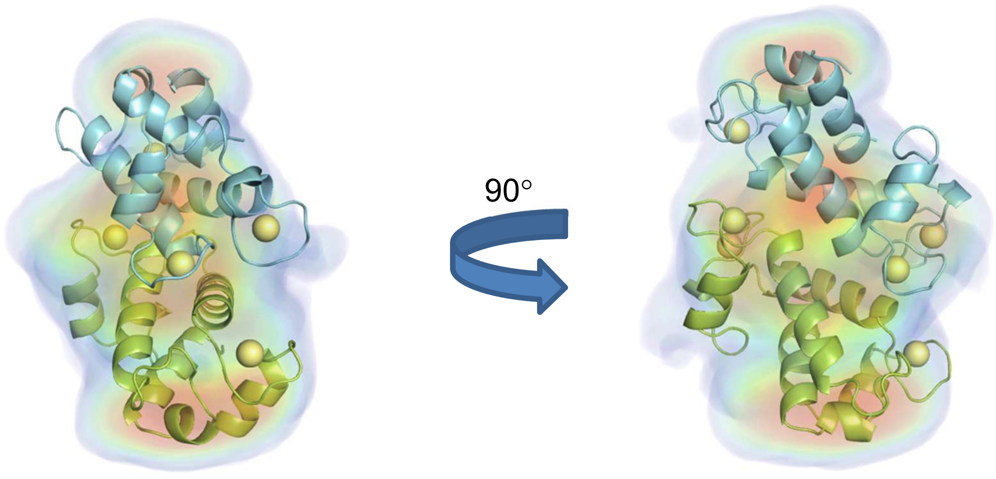

Fig. S14. Dy^{III}-bound *Hans*-LanM solvent envelope. The DENSS electron density map, shown as a transparent surface in the PyMOL-generated representation, overlays poorly with the dimeric La^{III}-*Hans*-LanM crystal structure. The CRYSOL fit of the calculated SAXS profile for the dimer overlays with the experimental SAXS profile with a Chi-squared fit of 3.8 (**Table S6**).

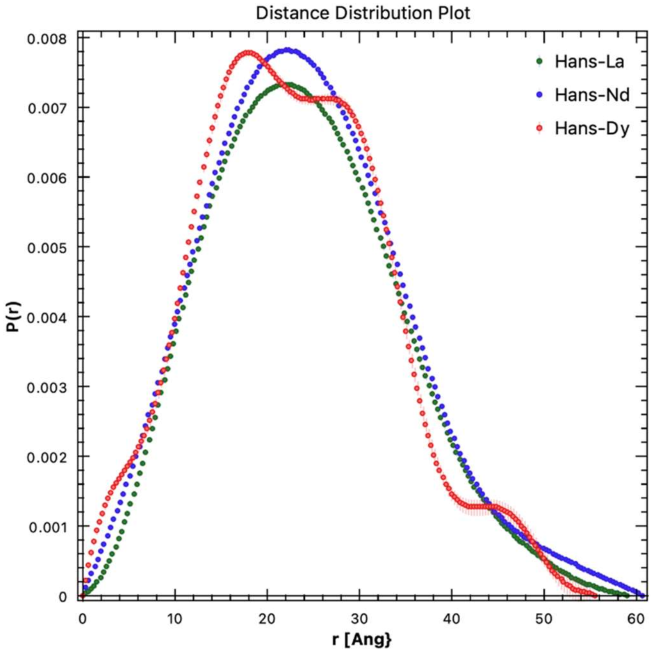

Fig. S15. Distance distribution (P(r)) analysis suggests that *Hans*-LanM undergoes a transition from a single species with La and Nd to two states with Dy. Overlay of the pair-wise distance distribution functions, P(r), for the La- (green), Nd- (blue), and Dy-bound (red) *Hans*-LanM datasets. The R_g values from this analysis – 18.5 Å (La), 18.7 Å (Nd), and 17.6 Å (Dy) – are similar to the R_g values obtained from the Guinier analysis. For La and Nd complexes, the P(r) has a bell shape, representative of scattering from a globular particle. In the case of Dy, P(r) shows three shoulders suggestive of a mixture of species (e.g., monomer/dimer mixture).

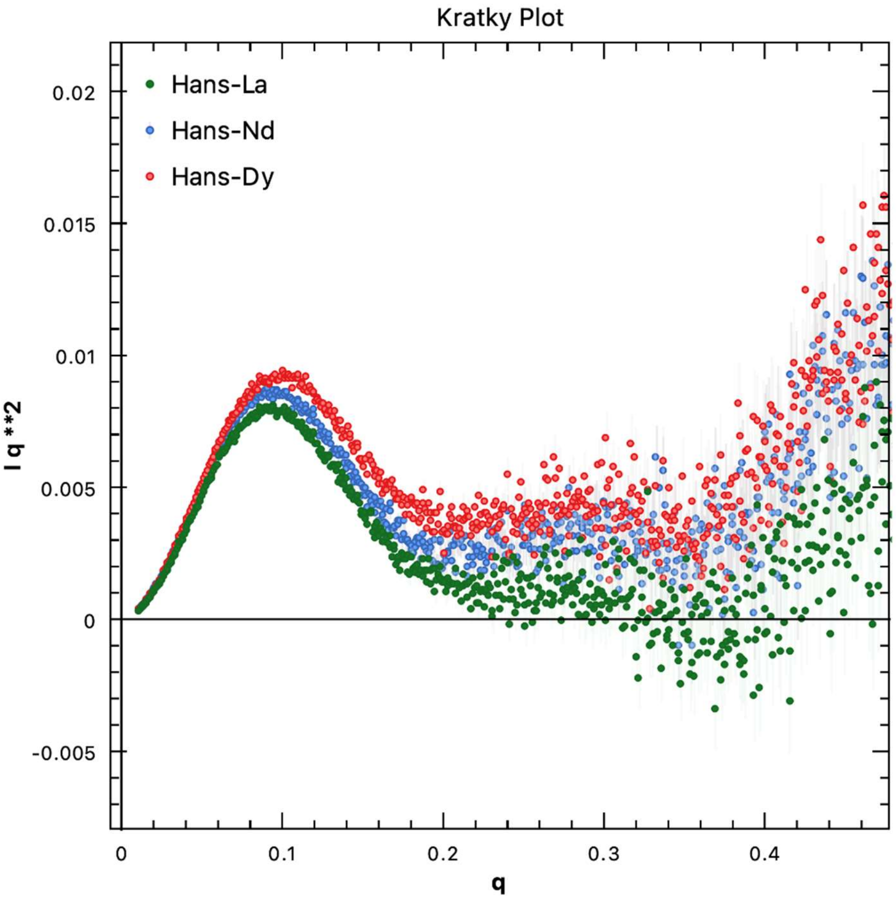

Fig. S16. Kratky plots suggest that *Hans*-LanM flexibility increases from La to Nd to Dy. Kratky plots for La- (green), Nd- (blue), and Dy-bound (red) *Hans*-LanM. Kratky plots derived from the SAXS data qualitatively inform on a protein's flexibility and/or foldedness.[11] The progressive divergence from the *q* axis from La- to Nd- to Dy-bound forms is suggestive of increasing disorder, which may relate to weaker Dy^{III} binding and reduced cooperativity (**Fig. 1d**) and/or to monomer-dimer equilibrium. Units of *q* are $Å^{-1}$.

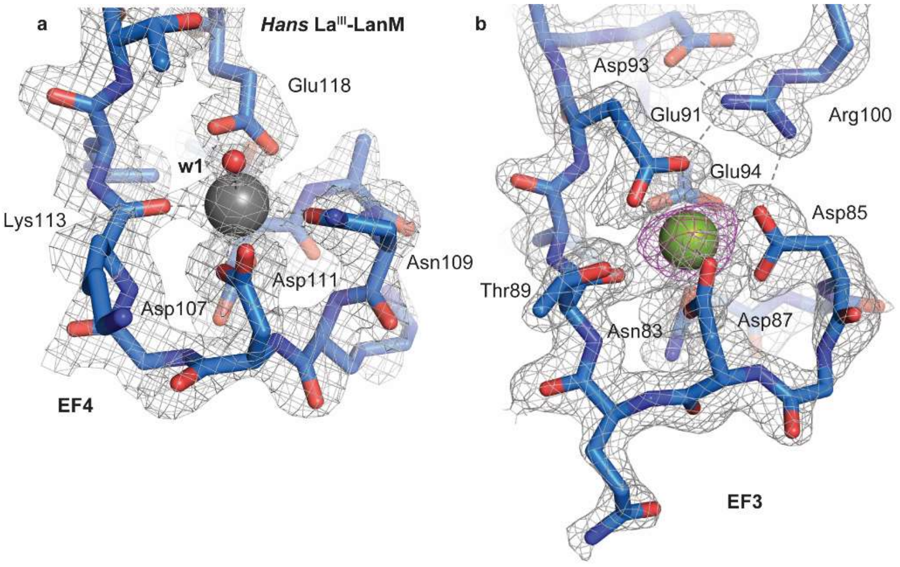

Fig. S17. $2F_o$-F_c electron density map (gray mesh, contoured at 1.0 σ) and anomalous difference map (purple mesh, contoured at 3.0 σ) of EF-hands 3 and 4 of La^{III}-bound *Hans*-LanM. **a,** In EF4, solvent is clearly indicated, and the lack of anomalous difference density shows that La^{III} does not occupy this site. The metal ion was modeled as a fully occupied Na^{I} for reasons described in **Extended Data Figure 3**. **b,** In EF3, representative of EF-hands 1-3, the electron density map suggests no solvent coordination, and the anomalous difference density map is consistent with a fully occupied La^{III} ion.

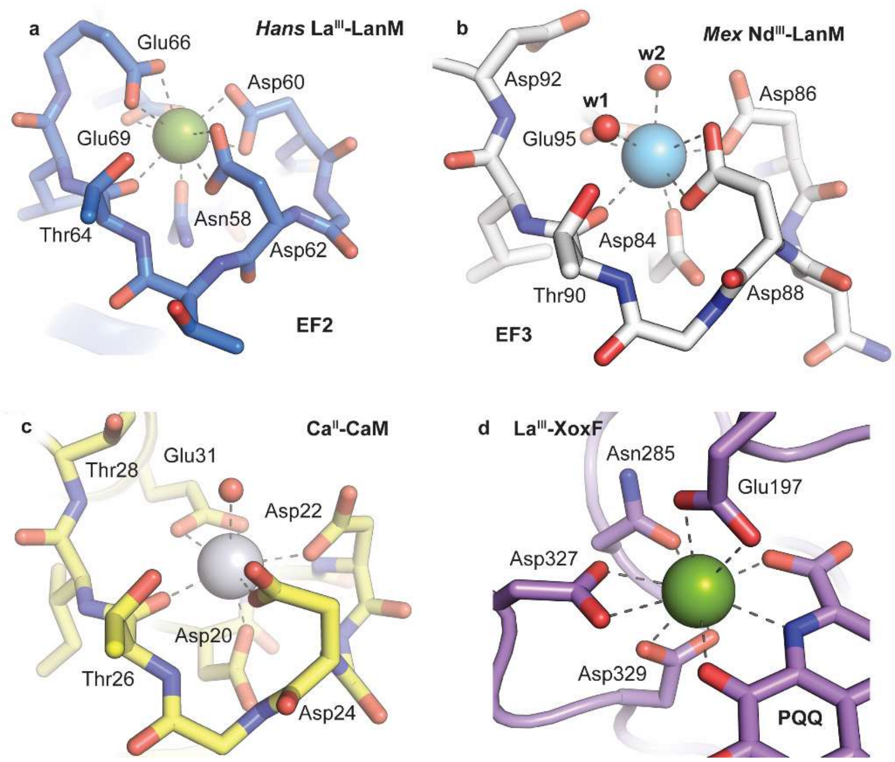

Fig. S18. Comparison of the structures of LanM EF-hands with an EF-hand from calmodulin (CaM) and with a lanthanide-dependent methanol dehydrogenase (MDH). **a,** EF2 from La^{III}-bound *Hans*-LanM. The La^{III} ion is a green sphere. **b,** EF3 from Nd^{III}-bound *Mex*-LanM. The Nd^{III} ion is an aqua sphere and coordinated solvent molecules (w1 and w2) are red spheres. **c,** EF2 from *H. sapiens* CaM (PDB code: 1CLL).[12] The Ca^{II} ion is a gray sphere and a coordinated solvent molecule is a red sphere. **d,** The active site of the lanthanide-dependent MDH, XoxF, from *Methylomicrobium buryatense* 5GB1C, metalated with La^{III} (1.85 Å resolution, PDB code: 6DAM).[13] The 10-coordinate La^{III} ion is shown as a green sphere. The enzyme also requires a pyrroloquinoline quinone cofactor (PQQ). The primary coordination spheres of Nd^{III}-*Mex*-LanM and Ca^{II}-CaM are nearly identical except the D_5 residue is monodentate in CaM (Asp24) but bidentate in *Mex*-LanM (Asp88) and there is an extra water molecule (w1) in *Mex*-LanM.

These images also highlight the consequence of *Hans*-LanM having an Asn at the 1st position of the EF-hands (N_1) versus an Asp (D_1) in *Mex*-LanM and CaM. This substitution induces a different peptide backbone structure between the 4th and 6th EF-hand positions. In *Hans*-LanM, the non-coordinated sidechain Nδ of Asn58 hydrogen bonds with the backbone CO of Asp62 residue (5th position), whereas in *Mex*-LanM and CaM the non-coordinated atom is an oxygen, enabling hydrogen bonding with the backbone NH of the 6th position residue. This difference is accommodated by *Hans*-LanM having a Gly at the 4th position in EF hands 2 and 3, whereas *Mex*-LanM has a Gly at the 6th position, and CaM has Gly residues at both positions (**Fig. S9b**).

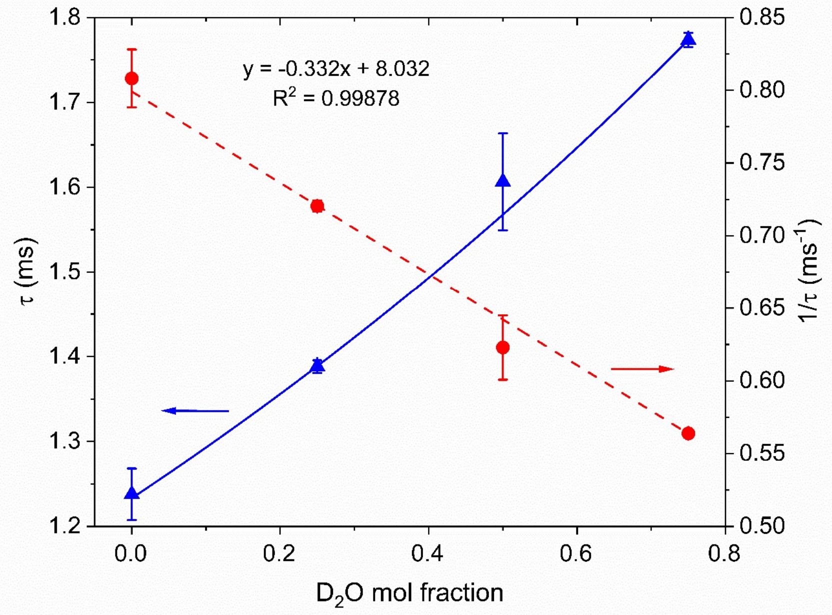

Fig. S19. Spectroscopic estimation of coordinated solvent molecules (q) in Eu^{III}_2-*Hans*-LanM using the Horrocks method. The luminescence lifetime of Eu^{III} complexes is empirically correlated with q.[14-16] Triangles: values for the luminescence decay time constant (τ, in ms, left y-axis). By way of comparison, τ_{H2O} = 1.24 ms for *Hans*-LanM but only 0.404 ms for *Mex*-LanM. Circles: 1/τ values (in ms^{-1}, right y-axis). The equation of the fit of 1/τ vs. mol fraction D_2O is used to determine q. The uncertainty in q is taken to be ±0.5.[14] The q value of 0.11 is consistent with the absence of coordinated solvent in the *Hans*-LanM crystal structures. Conditions: 20 μM *Hans*-LanM, 40 μM Eu^{III}, 25 mM HEPES, 75 mM NaCl, pH 7.0. Each data point is the mean ± s.d. for two independent samples.

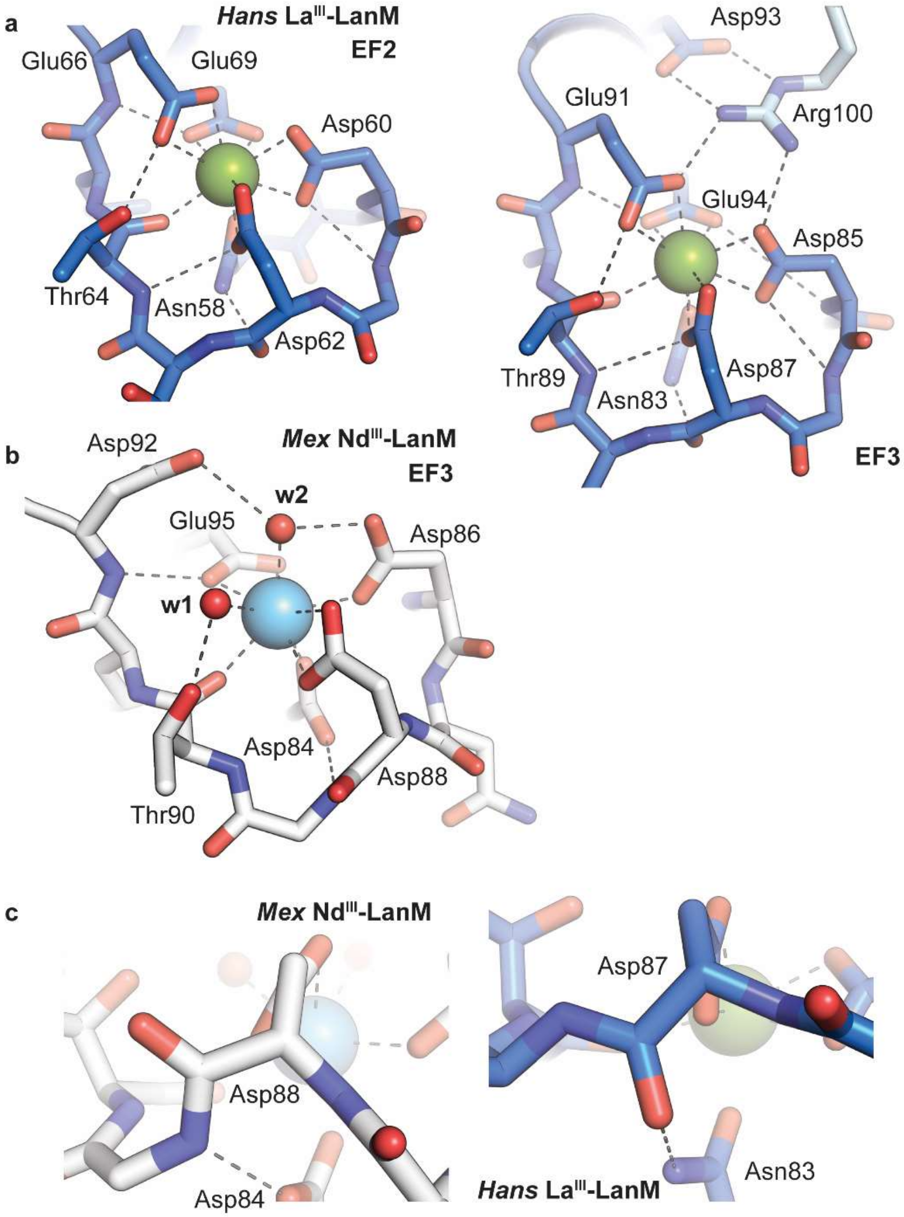

Fig. S20. Extended hydrogen bonding networks in the metal-binding sites of *Hans*- and *Mex*-LanMs. **a,** The metal sites of *Hans*-LanM display extensive hydrogen bonding between ligands and several backbone amides, as well as the sidechain of the Thr residue at the 7th position. In EF3, this network is extended further via interaction of Asp85 and Glu91 with Arg100 of the adjacent monomer. **b,** The metal sites of *Mex*-LanM feature similar hydrogen bonding patterns, but solvent molecules w1 and w2 take the place of Glu69. **c,** Enlarged view of the backbone flip that allows for hydrogen bonding interactions between the 1st position Asp and Asn residues and a mainchain amide and carbonyl in *Mex*-LanM and *Hans*-LanM, respectively.

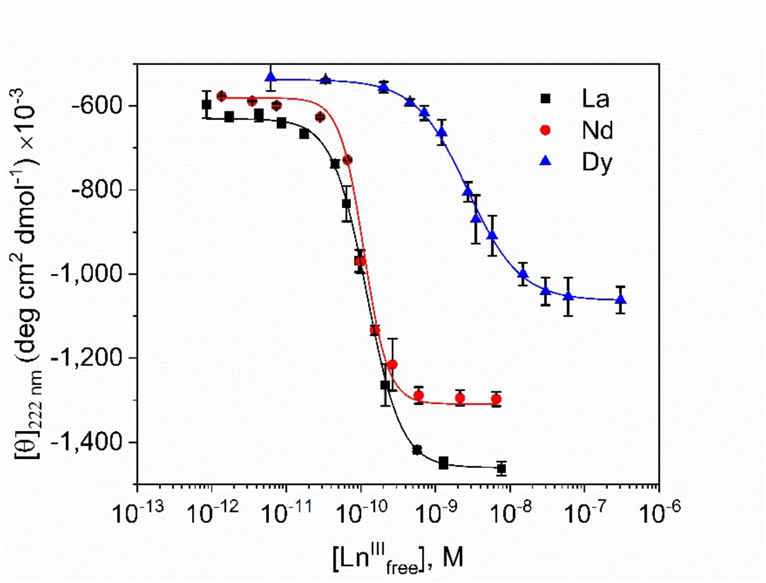

Fig. S21. Titration of R100K-*Hans*-LanM with La^{III}, Nd^{III}, and Dy^{III}, using CD spectroscopy (pH 5.0). Fitted parameters are summarized in **Table S7**. Results with Nd^{III} and Dy^{III} ($K_{d,app}$ and change in molar ellipticity) are essentially identical to those with the wild-type *Hans*-LanM protein; with La^{III}, a 2-fold weaker $K_{d,app}$ and larger change in molar ellipticity are observed. Conditions: 15 μM protein, 20 mM acetate, 100 mM KCl, pH 5.0, 10 mM EDTA (La,Nd) or EGTA (Dy), 0-10 mM RE^{III}. Each data point is the mean ± s.d. for two independent samples.

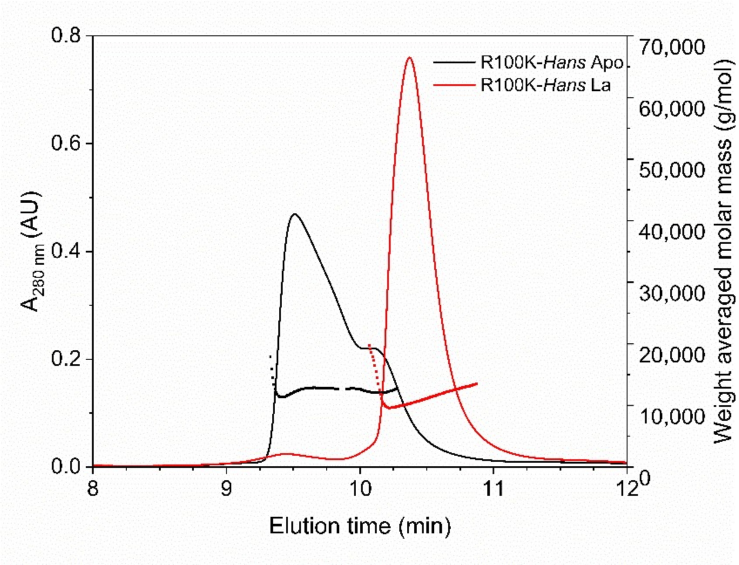

Fig. S22. SEC-MALS traces of R100K-*Hans*-LanM, as apoprotein and metalated with 3 equiv. La^{III}. The apoprotein migrates similarly to *Mex*-LanM and wild-type *Hans*-LanM (**Fig. S3**), indicative of a disordered protein. The La^{III} complex migrates as a single, sharp peak. Both samples were determined to have similar weight-averaged molar masses by MALS corresponding to a monomer (see **Table S8**). Conditions: 3 mg/mL protein, 30 mM MOPS, 100 mM KCl, pH 7.0.

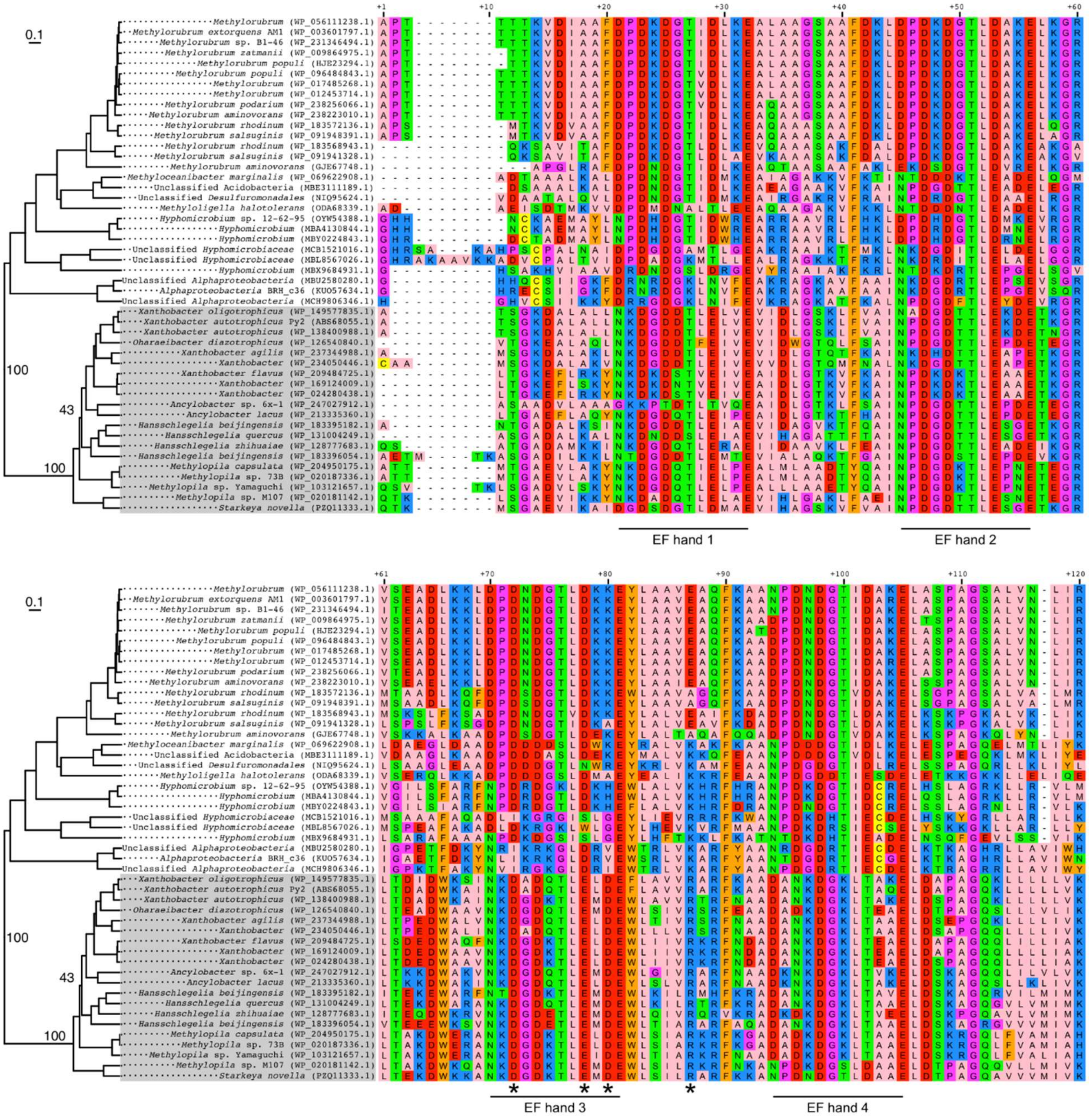

Fig. S23. *Hansschlegelia quercus* LanM and LanM proteins predicted to dimerize are phylogenetically distinct from other LanMs. The Bayesian phylogeny was constructed using a site-homogeneous model based on the Whelan and Goldman matrix[17] with invariant sites and four distinct gamma categories (WAG+I+Γ_4) under a strict clock with minimum sequence length of 106 amino acids. The monophyletic group including members of the Hans cluster is highlighted grey. Node values indicate posterior probabilities based on 10,000,000 iterations with a burn-in of 25%. The scale bar represents 0.1 changes per amino acid position. The LanM core sequence alignment used to construct the phylogeny is colored in the Zappos scheme. The four EF-hand domains are denoted with a line at the bottom of the alignment, and residues associated with dimer interaction with an asterisk. (Legend continued on next page.)

The Bayesian phylogeny constructed from this alignment supports the network structure, where the *Hans* cluster is represented as a monophyletic group and placed farther apart from other sequences. In addition, the topology of the *Hans* cluster in the phylogenetic tree corresponds to the proximity seen in the network (**Fig. 1a** and **Extended Data Figure 1**). In this alignment, R100 is in position 87, denoted with an asterisk along with the other three residues in EF-hand 3 involved in the dimerization interface (i.e., D72, E78, and D80). All four residues involved in dimerization are conserved in the *Hans* cluster LanMs, suggesting that these proteins all form dimers. Only a single LanM outside of the *Hans* cluster (an unclassified *Hyphomicrobiaceae*) has an Arg residue at position 87. The EF3 sequence in this ortholog lacks several Ln^{III} ligands, but EF2 features the D_{11} residue that (in EF3) in *Hans* mediates interaction with the Arg, suggesting that this uncharacterized LanM may dimerize along a distinct interface. No sequences other than those included in this alignment contained a basic amino acid in position 87 – instead most, like *Mex*-LanM, have an acidic residue (usually Glu).

A group of LanMs containing two cysteine residues, one near each terminus (in proximity to EF1 and EF4), was identified. Given that LanMs are periplasmic proteins, we propose that these proteins have a disulfide bond between these residues, which may provide added structural stability to LanM, but more evidence is required to ascertain the role of these residues.

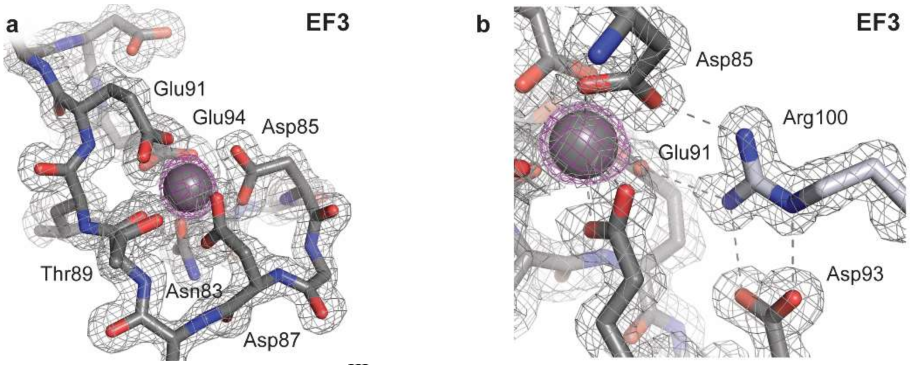

Fig. S24. Two views of EF-hand 3 of Dy^{III}-*Hans*-LanM with the 2F_o-F_c electron density map (gray mesh, contoured at 1.0 σ) and anomalous difference map (purple mesh, contoured at 3.0 σ) shown. **a,** Density associated with metal ligands is clear and no coordinated solvent is apparent. **b,** The hydrogen bonding network between EF3 and Arg100 of the adjacent monomer is also clearly visualized.

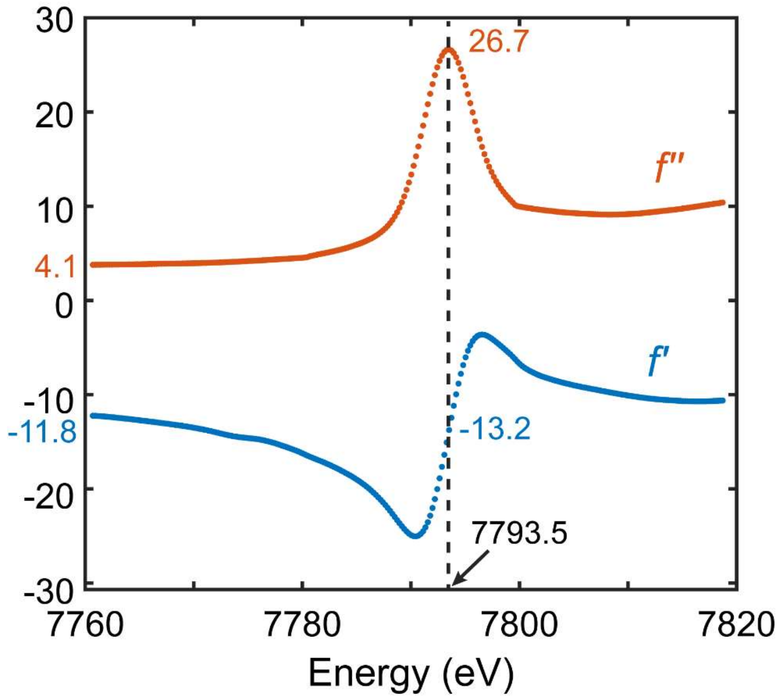

Fig. S25. X-ray absorption edge of Dy-*Hans*-LanM detected by fluorescence excitation.

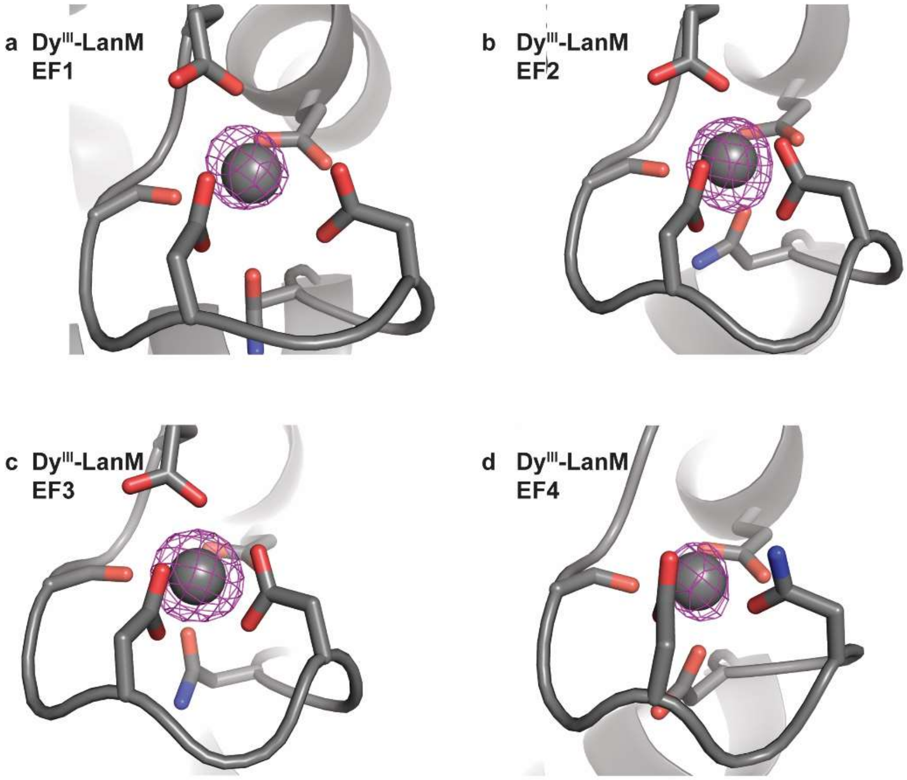

Fig. S26. Anomalous diffraction datasets collected at the L_{III} edge (7793.5 eV) for Dy on Dy^{III}-*Hans*-LanM crystals supports assignment of the bound lanthanide as the HRE, Dy. Anomalous difference electron density map is shown in purple mesh (contoured at 4.0σ) for representative metal binding sites in chain A. In all four EF hands, we observe significantly more intense anomalous difference electron density map peaks above the L_{III} edge (**Table S10**). Interestingly, EF2 and EF3 exhibit the largest anomalous difference map peaks, perhaps reflecting the biochemical observation of only two high-affinity sites in this complex (**Fig. 1d, Fig. S2**).

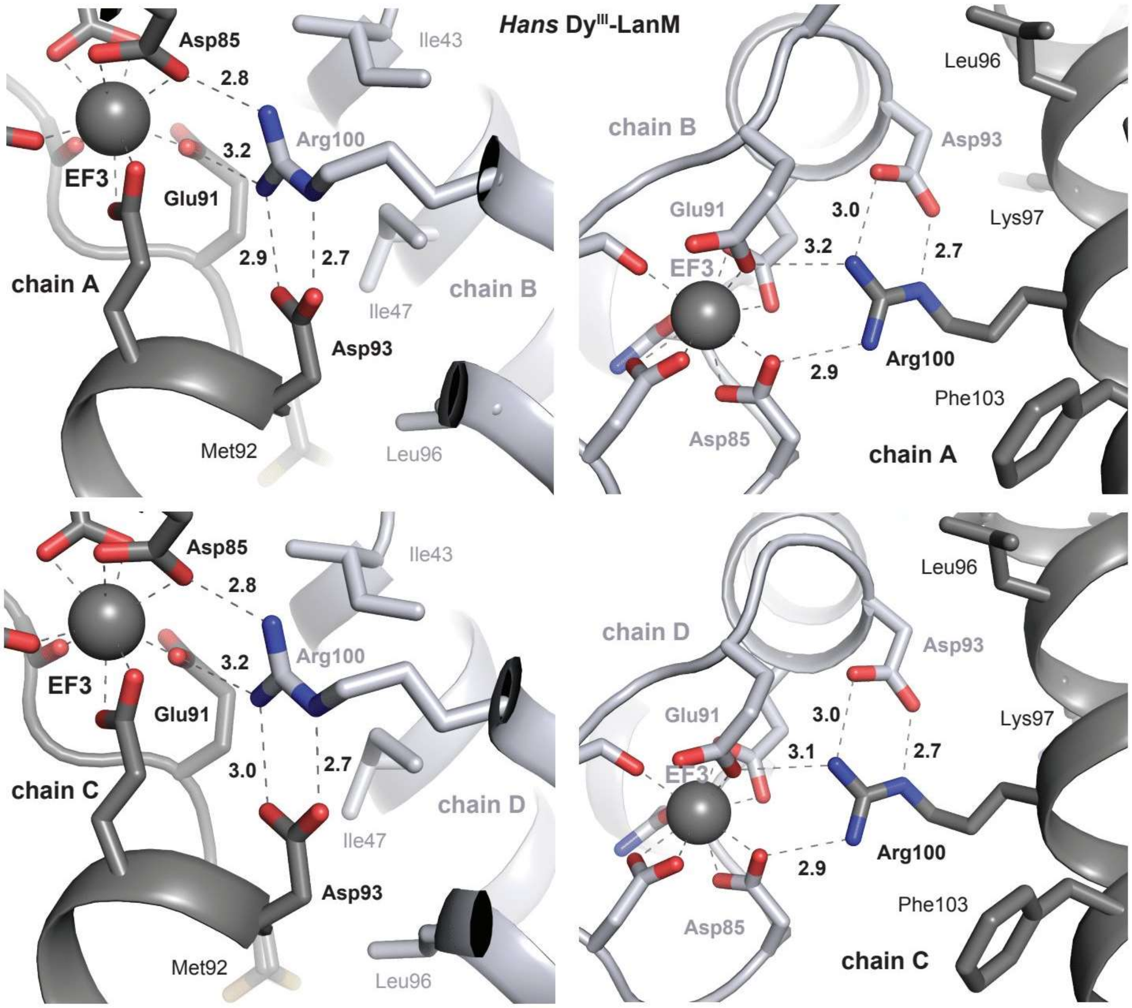

Fig. S27. All four copies of the Arg100-EF3 hydrogen bonding network in the asymmetric unit of Dy^{III}-*Hans*-LanM show the same shift to monodentate coordination in Glu91 along with lengthening of the hydrogen bond between this residue and Arg100, from ~2.9 Å in La^{III}-*Hans*-LanM to 3.2 Å in Dy^{III}-*Hans*-LanM. One of the Arg100-Asp93 hydrogen bonds also lengthens from 2.5 Å in La^{III}-*Hans*-LanM to 2.7 Å. The Arg100-Asp85 hydrogen bond compresses slightly from 3.2 Å in La^{III}-*Hans*-LanM to 2.8/2.9 Å in Dy^{III}-*Hans*-LanM. Although it is possible that forcing dimerization under the high-concentration conditions for crystallography may alter the interactions between monomers relative to those at lower concentration in solution, the La- and Dy-bound structures illustrate how the carboxylate shift of Glu91 can alter this second-sphere hydrogen bonding network, which would plausibly disfavor dimerization, an explanation strongly supported by characterization of the R100K variant (**Table S8**, **Figs. S21-S23**).

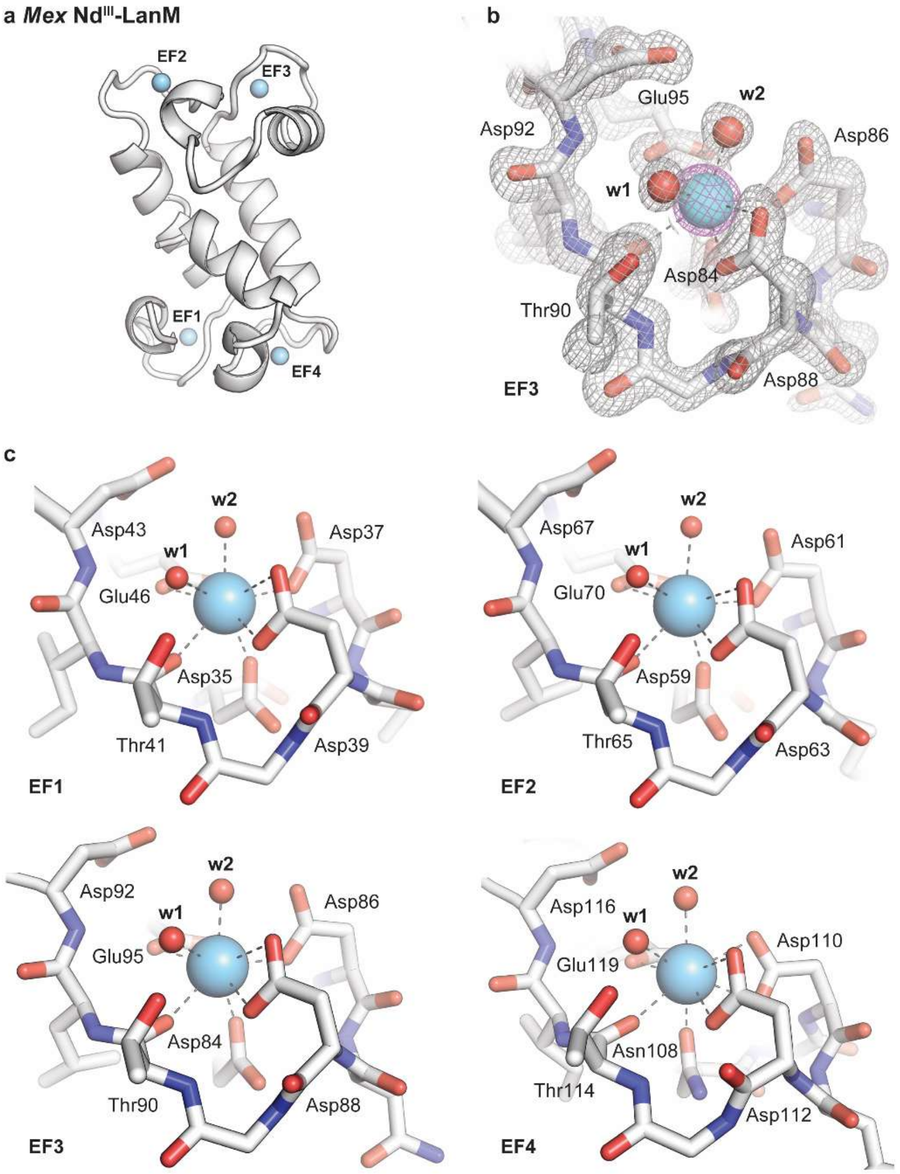

Fig. S28. X-ray crystal structure of Nd^{III}-bound *Mex*-LanM, solved at 1.01 Å resolution. **a,** Overall structure of *Mex*-LanM. The Nd^{III} ion in EF4 is present due to the crystallization conditions (3.5 equiv. Nd^{III} and millimolar protein); prior biochemical analyses have shown that the weakest binding equivalent is with micromolar K_d and that the weak site is associated with EF4.[2,18] **b,** $2F_o$-F_c electron density map (gray mesh, contoured at 1.0 σ) and anomalous difference map (purple mesh, contoured at 3.0 σ) of EF3, showing the two solvent molecules coordinated to the Nd^{III} ion. The Nd^{III} ion is an aqua sphere, and solvent molecules are red spheres. **c,** Details of the four EF-hands. Metal coordination in EF1-3 is identical, with D_1, D_3, and the backbone CO of T_7 being monodentate ligands, D_5 and E_{12} being bidentate ligands, and two water molecules (w1, w2) yielding 9-coordination. In EF4, the D_3 residue (Asp110) is bidentate. Because EF4 possesses an Asn at the 1st position rather than an Asp, the non-coordinated sidechain N cannot hydrogen bond with the backbone, which may contribute to the lower affinity of this site.

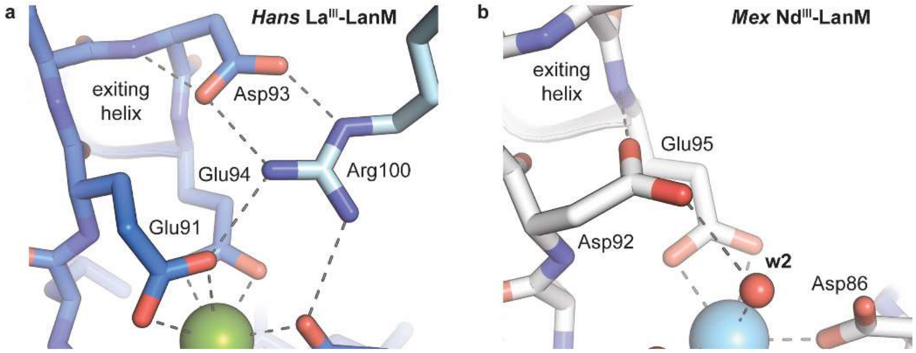

Fig. S29. Comparison of hydrogen bonding networks connecting metal-binding sites to the exiting helices in La^{III}-*Hans*-LanM and Nd^{III}-*Mex*-LanM. These extensions of the backbone CO-HN hydrogen bonding networks within each helix to include the metal sites may contribute to the overall stabilization of the folded state of the proteins in the RE^{III}-LanM complexes. **a,** In La^{III}-*Hans*-LanM, although the E_9 residues are bidentate ligands and therefore a directly analogous hydrogen bond cannot occur, Glu91 in EF3 is connected to the first backbone NH of the exiting helix via the hydrogen bonding network involving Arg100 and Asp93. The disruption of this network, and therefore a connection between the helix and the metal site, in the presence of the HREs may also contribute to the lower stability of the HRE-*Hans*-LanM complexes. **b,** In Nd^{III}-*Mex*-LanM, the D_9 residues (Asp92 in EF3) directly connect the Glu95 NH from the exiting helix to Nd^{III}-coordinated solvent (w2). The different lengths of these two hydrogen bonds that would result from coordination of different REs may contribute to the selectivity trend in *Mex*-LanM.

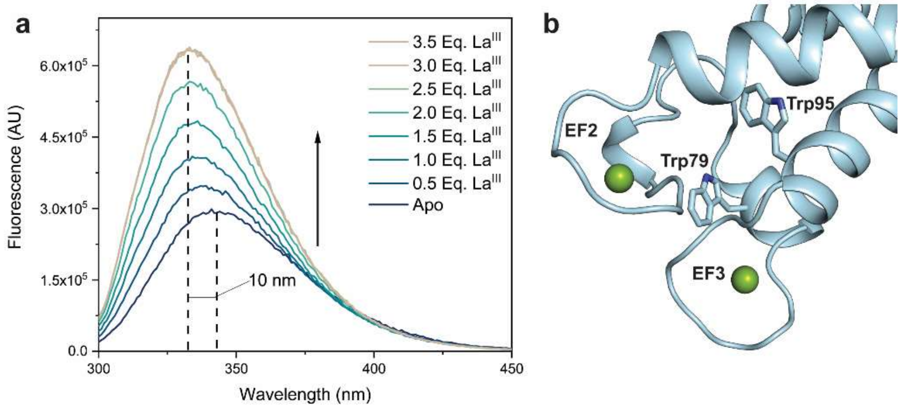

Fig. S30. Representative fluorescence emission intensity and wavelength (λ_{max}) changes from single measurements of wild-type *Hans*-LanM during titration with La^{III}. **a,** Emission spectra of *Hans* (λ_{ex} = 278 nm). La^{III} binding increases intensity 2-fold and shifts the λ_{max} from 343 nm to 333 nm. **b,** The excitation and emission wavelengths suggest the protein's two Trp residues, Trp79 and Trp95, near EF2 and EF3, primarily contribute to the spectra. Trp95 aligns with Tyr96 of *Mex*-LanM (**Fig. S1**), the fluorescence intensity of which has been shown to also be sensitive to metal binding and/or the associated protein conformational change.[2] Conditions: 20 µM protein, 30 mM MOPS, 100 mM KCl, pH 7.0.

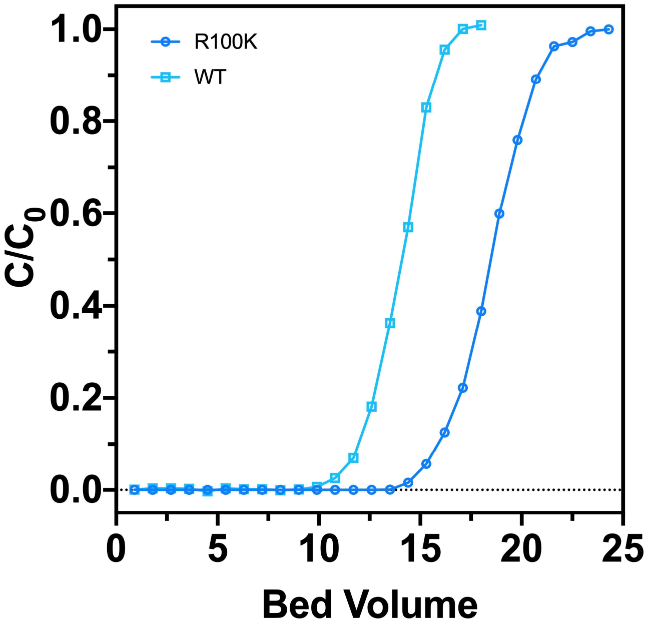

Fig. S31. Representative Nd breakthrough curves for column-immobilized *Hans*-LanM and R100K-*Hans*-LanM. Experiments used 0.4 mM Nd^{III} in 7 mM homo-PIPES, pH 5.0. For *Hans*-LanM, 4.4 ± 0.08 µmol/mL protein was immobilized, with a Nd adsorption capacity of 4.6 ± 0.23 µmol/mL (1.06 equiv.). For R100K-*Hans*-LanM, 3.2 ± 0.06 µmol/mL protein was immobilized, with a Nd adsorption capacity of 6.66 ± 0.33 µmol/mL (2.08 equiv.). Each protein was immobilized once; the uncertainties in the immobilized protein represent s.d. from 3 replicate protein concentration determinations by BCA assay, and the uncertainties in the adsorption capacities were assumed to be 5% based on our previously reported LanM column experiments.

SUPPLEMENTARY TABLES

Table S1. ICP-MS analysis of metalated *Hans*-LanM samples used for SAXS or crystallography. Samples for SAXS analysis were incubated with 3 equiv. metal ion, and soluble protein was run on a S75 SEC column to remove aggregates. Preparation of samples for crystallography is described in detail in the Methods. The lower metal loading for the samples used for crystallography may relate to the additional spin concentration step and the third equivalent binding to a relatively weak site. The data are expressed as mean with s.d. from 3 technical replicates.

	Metal ion	Protein concentration (μM)	Metal concentration (μM)	Equivalents
SAXS	La	120	336(8)	2.8
	Nd	114	349(2)	2.9
	Dy	128	269(16)	2.4
Crystallography	La	1360	2310(60)	1.7
	Dy	1450	2300(60)	1.6

Table S2. Summary of fitted parameters for CD titrations of *Hans*-LanM with La^{III}, Nd^{III}, Dy^{III}, and Ca^{II}. Apparent K_d ($K_{d,app}$) values, Hill coefficients (n), and changes in molar ellipticity at 222 nm (Δ[Θ]) are reported as mean ± s.e.m. for fits of data from 3 independent titrations. Conditions: 15 μM monomer concentration, 25 °C, 20 mM acetate, 100 mM KCl, pH 5.0.

**Metal ion**	$K_{d,app}$	n	**Δ[Θ] (deg cm^2 dmol^{-1} × 10^{-3})**
La^{III}	68(7) pM	1.8(6)	-728(57)
Nd^{III}	91(6) pM	2.0(2)	-722(20)
Dy^{III}	2600(700) pM	1.3(4)	-449(56)
Ca^{II}	60(10) μM	1.0(2)	-545(41)

Table S3. Apparent molecular weights of RE^{III}-*Hans*-LanM complexes from analytical SEC (Superdex S75). Apparent MW values were derived from **Fig. S4** and also plotted in **Fig. 2a**. Compare with SEC-MALS data in **Extended Data Table 1**. Ionic radii are given for the 10-coordinate (La) and 9-coordinate (Nd-Ho) complexes from Shannon.[19] See **Fig. S4** legend for experimental details.

RE^{III} ion	Ionic radius (Å)	Apparent MW (SEC, kDa)
La	1.16[a]	27.8
Nd	1.11[b]	27.8
Sm	1.08[b]	26.1
Eu	1.07[b]	25.5
Gd	1.05[b]	23.0
Tb	1.04[b]	21.3
Dy	1.03[b]	20.0
Ho	1.02[b]	17.9

[a] CN=10. [b] CN=9.

Table S4. Data collection and refinement statistics for the X-ray structures of La- and Dy-*Hans*-LanM and Nd-*Mex* LanM. Statistics for the highest resolution shell are shown in parentheses.

	La-*Hans*-LanM	Dy-*Hans*-LanM	Nd-*Mex*-LanM
**Wavelength (Å)**	0.97857	0.97857	0.97857
**Resolution range**	40.11 - 1.8 (1.86 - 1.8)	24.84 - 1.4 (1.45 - 1.4)	23.13 - 1.01 (1.046 - 1.01)
**Space group**	$P\ 2_1$	$P\ 2_1$	$P\ 2_1\ 2_1\ 2_1$
**Unit cell**	40.107 108.658 44.697 90 90.024 90	56 54.234 65.275 90 93.567 90	24.099 44.398 82.316 90 90 90
**Total reflections**	184147 (8072)	566280 (43337)	544124 (7577)
**Non-anomalous unique reflections**	27161 (1945)	75504 (6019)	43881 (2296)
**Multiplicity**	6.8 (4.2)	7.5 (7.2)	12.4 (3.3)
**Completeness (%)**	72.2 (29.8)	97.5 (78.3)	91.0 (41.0)
**Mean I/sigma(I)**	12.9 (1.3)	28.0 (2.3)	26.7 (2.4)
**Wilson B-factor**	17.53	12.72	16.20
**R-meas**	0.133 (1.13)	0.069 (1.08)	0.076 (0.56)
**R-pim**	0.052 (0.53)	0.025 (0.40)	0.021 (0.29)
**CC1/2**	0.995 (0.608)	0.997 (0.740)	0.986 (0.752)
**Reflections used in refinement**	25533	75499	43022
**Reflections used for R-free**	1286	1985	1949
**R-work**	0.1898	0.1614	0.1453
**R-free**	0.2088	0.1809	0.1524
**Number of non-hydrogen atoms**	3641	3979	964
**macromolecules**	3336	3342	793
**ligands**	42	14	4
**solvent**	263	623	167
**Protein residues**	440	435	105
**RMS(bonds)**	0.003	0.007	0.006
**RMS(angles)**	0.48	0.80	0.81
**Ramachandran favored (%)**	96.53	99.06	100
**Ramachandran allowed (%)**	3.47	0.94	0.00
**Ramachandran outliers (%)**	0.00	0.00	0.00
**Rotamer outliers (%)**	0.00	0.57	0.00
**Clashscore**	0.60	2.23	3.79
**Average B-factor**	26.04	25.86	24.49
**macromolecules**	25.45	24.09	22.62
**ligands**	46.04	21.95	19.42
**solvent**	30.33	35.47	33.49

Table S5. SAXS structural parameters for *Hans*-LanM in presence of different metal ions. Data were collected on an in-house Rigaku BioSAXS2000^{nano}. Buffer conditions: 30 mM MOPS, 100 mM KCl, 5% glycerol, pH 7.0. The Porod volume analysis supports the conclusion that La and Nd complexes of *Hans*-LanM are largely dimeric and the Dy complex, only ~2/3 the volume of the La and Nd complexes, is an equilibrium mixture of monomer and dimer. Note that there is less difference between the R_g values for the Dy and the La/Nd complexes than might be expected based on SEC-MALS (**Extended Data Table 1** suggests a ~2-3 Å difference in hydrodynamic radius for La/Nd vs. Dy), because the protein concentrations used for SAXS are 5-fold higher than those for SEC-MALS, and therefore the population of Dy-bound dimer is substantially larger in the SAXS experiment. Nevertheless, these RE-dependent differences are within the uncertainty of the SAXS R_g values (see **Fig. S11**).

	**La-*Hans*-LanM**	**Nd-*Hans*-LanM**	**Dy-*Hans*-LanM**
Concentration prior to SEC-MALS (mg/mL)	1.2	1.6	1.4
Guinier analysis			
$I(0)$ (cm^{-1})	2.46	2.67	2.62
R_g (Å)	18.5	18.7	17.8
q min (Å $^{-1}$)	0.01	0.01	0.01
q max (Å $^{-1}$)	0.07	0.07	0.07
P(r) analysis			
$I(0)$ (cm^{-1})	2.46	2.67	2.62
R_g (Å)	18.5	18.7	17.8
D_{max} (Å)	59.0	60.6	55.6
q range (Å $^{-1}$)	.01-.43	.01-.42	.01-.44
Porod volume (Å^3)	33261	29999	22599

Table S6. Analyses using OLIGOMER and CRYSOL software suggest that the La and Nd complexes of *Hans*-LanM are nearly completely dimeric, but the Dy complex is a mixture of monomer and dimer. The OLIGOMER program fits an experimental SAXS scattering curve from a multicomponent mixture of proteins to find the volume fractions of each component in the mixture.[20] CRYSOL evaluates the solution scattering from macromolecules with known atomic structure and fits it to experimental scattering curves.[21] Both analyses were done with two possible states: the La-*Hans*-LanM crystallographic dimer and a crystallographic monomer. The analysis indicates that the volume fractions for the La- and Nd-*Hans*-LanM complexes are preferentially dimeric, likely close to the true distributions because of the availability of the crystallographic dimer model. OLIGOMER and CRYSOL analyses for Dy condition suggests an equilibrium between dimer and monomer.

	**La-*Hans*-LanM**	**Nd-*Hans*-LanM**	**Dy-*Hans*-LanM**
OLIGOMER: Volume fraction of dimer	0.94(1)	0.88(1)	0.55(5)
OLIGOMER: Volume fraction of monomer	0.06(1)	0.12(1)	0.45(3)
CRYSOL: Chi^2 fit to dimer	1.1	1.3	3.8
CRYSOL: Chi^2 fit to monomer	22.6	16.5	8.7

Table S7. Summary of fitted parameters for CD titrations of R100K-*Hans*-LanM with La^{III}, Nd^{III} and Dy^{III}. Apparent K_d ($K_{d,app}$) values, Hill coefficients (n), and changes in molar ellipticity at 222 nm ($\Delta[\Theta]$) are reported as mean ± s.e.m. for fits of data from 2 (Nd, Dy) or 3 (La) independent titrations. Conditions: 15 µM monomer concentration, 25 °C, 20 mM acetate, 100 mM KCl, pH 5.0.

**Metal ion**	$K_{d,app}$ **(pM)**	n	$\Delta[\Theta]$ **(deg cm^2 dmol^{-1} × 10^{-3})**
La	120(10)	1.9(2)	-829(28)
Nd	99(7)	2.8(5)	-697(32)
Dy	2700(400)	1.3(2)	-525(29)

Table S8. SEC-MALS analysis of R100K-*Hans* LanM. The concentrations of the protein samples loaded to the column were 3 mg/mL (3.5 times more concentrated than wild-type *Hans*-LanM samples).

	**Peak retention time (min)**	**Molecular weight (kDa)**	**Polydispersity**	**Hydrodynamic radius (nm)**
Apo	9.55	13.1	1.043	1.32
	10.1	12.4	1.000	N.D.[a]
La	10.4	10.9	1.010	1.25
Dy	10.3	9.9	1.005	1.27

[a] N.D., not determined

Table S9. Data collection statistics for the anomalous diffraction datasets collected on Dy-*Hans*-LanM at 7760.0 (pre-edge) and 7793.5 eV (L_{III} edge of Dy). Statistics for the highest resolution shell are shown in parentheses.

	Dy-*Hans* LanM anomalous	
**Wavelength (Å)**	1.59774	1.59087
**Resolution range**	43.56 – 2.22<br>(2.30 – 2.22)	43.56 – 2.21<br>(2.29 – 2.21)
**Space group**	P 2_1	P 2_1
**Unit cell**	55.514 54.758 64.423<br>90 94.2 90	55.525 54.748 64.421<br>90 94.2 90
**Total reflections**	228251<br>(17496)	233868<br>(18403)
**Anomalous unique reflections**	36946<br>(3392)	37716<br>(3694)
**Multiplicity**	6.2<br>(5.2)	6.2<br>(5.0)
**Anomalous completeness (%)**	97.8<br>(90.4)	99.4<br>(96.8)
**Mean I/sigma(I)**	21.9<br>(10.6)	20.7<br>(9.6)
**Wilson B-factor**	26.33	25.89
**R-meas**	0.117<br>(0.252)	0.118<br>(0.240)
**R-pim**	0.033<br>(0.081)	0.033<br>(0.076)
**$CC_{1/2}$**	0.958<br>(0.608)	0.994<br>(0.981)

Table S10. Anomalous peak heights for Dy-*Hans*-LanM at 7760.0 (pre-edge) and 7793.5 eV (L_{III} edge of Dy) in units of e/Å^3. Interestingly, EF2 and EF3 exhibit the largest anomalous difference map peaks, perhaps reflecting the biochemical observation of only two high-affinity sites in this complex (**Fig. 1d, Fig. S2**). N/A: not applicable. *No heavy elements are found in EF1 of chain D in this crystal.

Crystal 1	Chain A		Chain B		Chain C		Chain D	
Energy (eV)	7793.5	7760.0	7793.5	7760.0	7793.5	7760.0	7793.5	7760.0
EF1	0.699	0.109	N/A	N/A	N/A	N/A	N/A*	N/A*
EF2	1.513	0.224	1.737	0.269	1.692	0.265	1.564	0.226
EF3	1.532	0.246	1.526	0.246	1.711	0.275	1.846	0.278
EF4	0.564	0.124	0.379	0.108	0.583	0.146	0.962	0.176

Table S11. Concentrations of citrate and malonate required for 50% desorption of La^{III}, Nd^{III}, Dy^{III} from *Hans*-LanM and R100K-*Hans* LanM. See **Fig. 4a** and **Extended Data Figure 5**. Trp emission intensity changes in *Hans*-LanM were monitored at 333 nm. Initial conditions: 20 µM protein, 40 µM RE, 20 mM acetate, 100 mM KCl, pH 5.0, into which increasing concentrations of citrate or malonate were titrated. Data represent mean ± s.e.m. (in parentheses) for fits of data from 3 independent titrations.

	**[citrate]$_{1/2}$ (mM)**	**[malonate]$_{1/2}$ (mM)**
***Hans*-LanM**		
La	18(3)	–
Nd	7.5(8)	>350[a]
Dy	1.0(1)	144(17)
**R100K-*Hans*-LanM**		
La	12(1)	–
Nd	5.6(6)	>350
Dy	1.0(1)	128(11)

[a] 350 mM was the highest concentration tested. See **Fig. 4b**.

Table S12. Distribution factors (*D*, in bold) and separation factors (SF) for selected REs for immobilized *Mex*-LanM. *D* values represent distribution of a particular metal ion between LanM and the solution in the multi-element equilibration experiment described in the main text and Materials and Methods ("Batch experiment to determine separation factors"), in which a 5 mL feed solution of equimolar REs from La to Dy (3 mM total REs, 15 µmol total, pH 5.0) reaches equilibrium with 1 mL immobilized LanM microbeads (capacity: 5.8 µmol for *Mex*-LanM, 4.1 µmol for *Hans*-LanM, 4.7 µmol for R100K-*Hans*-LanM). Larger *D* values indicate preferential adsorption to LanM. Separation factors as a function of RE identity were calculated as $D_{metal(top)}/D_{metal(left)}$. An SF of 1.0 indicates no selectivity for intra-RE separation, while SFs much greater (or less) than 1 indicate preference for a particular ion over another. See **Fig. 4c** for a plot of the common logarithm of *D* values for each metal ion.

Uncertainties are shown in parentheses, where the number is parentheses is the uncertainty in the significant digits immediately preceding: e.g., 0.86(1) means 0.86 ± 0.01, and 2.15(32) means 2.15 ± 0.32. The uncertainties are larger for HREs like Dy, especially with *Hans*-LanM and R100K-*Hans*-LanM, because the weaker binding to LanM means quantities of REs adsorbed to the column are very low compared to the LREs. Uncertainties for *D* values are standard deviations from three independent column runs. Uncertainties for SF values were calculated by error propagation of the corresponding *D* values.

		**La**	**Ce**	**Pr**	**Nd**	**Sm**	**Eu**	**Gd**	**Tb**	**Dy**
	D	**0.86(1)**	**1.52(3)**	**1.90(4)**	**2.15(32)**	**2.19(5)**	**1.68(2)**	**0.93(2)**	**0.65(1)**	**0.41(1)**
**La**	**0.86(1)**	1	1.77(5)	2.21(6)	2.50(37)	2.55(8)	1.95(4)	1.08(3)	0.75(2)	0.48(1)
**Ce**	**1.52(3)**	0.57(2)	1	1.25(4)	1.42(21)	1.44(5)	1.10(3)	0.61(2)	0.43(1)	0.27(1)
**Pr**	**1.90(4)**	0.45(1)	0.80(2)	1	1.13(17)	1.15(4)	0.88(2)	0.49(1)	0.34(1)	0.22(1)
**Nd**	**2.15(32)**	0.40(6)	0.71(11)	0.88(13)	1	1.02(15)	0.78(12)	0.43(6)	0.30(4)	0.19(3)
**Sm**	**2.19(5)**	0.39(1)	0.69(2)	0.87(3)	0.98(15)	1	0.76(2)	0.42(1)	0.30(1)	0.19(1)
**Eu**	**1.68(2)**	0.51(1)	0.91(2)	1.13(3)	1.28(19)	1.31(4)	1	0.55(1)	0.39(1)	0.25(1)
**Gd**	**0.93(2)**	0.93(3)	1.64(5)	2.05(6)	2.32(35)	2.37(8)	1.81(4)	1	0.70(2)	0.44(1)
**Tb**	**0.65(1)**	1.33(4)	2.34(7)	2.93(8)	3.31(49)	3.38(11)	2.58(6)	1.43(4)	1	0.63(2)
**Dy**	**0.41(1)**	2.09(6)	3.70(12)	4.63(14)	5.23(78)	5.33(19)	4.08(12)	2.25(7)	1.58(5)	1

Table S13. Distribution factors (*D*) and separation factors (SFs) for selected REs for immobilized *Hans*-LanM. See legend for **Table S12** for full details.

		**La**	**Ce**	**Pr**	**Nd**	**Sm**	**Eu**	**Gd**	**Tb**	**Dy**
	D	**0.86(3)**	**1.54(4)**	**1.71(5)**	**1.44(4)**	**0.99(4)**	**0.64(3)**	**0.34(2)**	**0.22(2)**	**0.15(2)**
**La**	**0.86(3)**	1	1.79(9)	1.98(10)	1.67(8)	1.14(6)	0.75(5)	0.39(3)	0.25(2)	0.17(2)
**Ce**	**1.54(4)**	0.56(3)	1	1.11(5)	0.93(4)	0.64(3)	0.42(2)	0.22(1)	0.14(1)	0.10(1)
**Pr**	**1.71(5)**	0.51(3)	0.90(4)	1	0.84(4)	0.58(3)	0.38(2)	0.20(1)	0.13(1)	0.09(1)
**Nd**	**1.44(4)**	0.60(3)	1.07(5)	1.19(5)	1	0.69(3)	0.45(3)	0.23(2)	0.15(1)	0.10(1)
**Sm**	**0.99(4)**	0.87(5)	1.56(7)	1.73(8)	1.46(7)	1	0.65(4)	0.34(2)	0.22(2)	0.15(2)
**Eu**	**0.64(3)**	1.34(9)	2.39(14)	2.65(16)	2.23(13)	1.53(9)	1	0.52(4)	0.34(3)	0.23(3)
**Gd**	**0.34(2)**	2.56(19)	4.58(31)	5.07(35)	4.26(30)	2.93(21)	1.91(15)	1	0.65(7)	0.44(6)
**Tb**	**0.22(2)**	3.93(38)	7.02(65)	7.78(73)	6.54(61)	4.50(43)	2.94(30)	1.53(17)	1	0.67(10)
**Dy**	**0.15(2)**	5.86(70)	10.49(1.21)	11.61(1.34)	9.77(1.13)	6.71(79)	4.39(54)	2.29(29)	1.49(21)	1

Table S14. Distribution factors (*D*) and separation factors (SFs) for selected REs for immobilized R100K-*Hans*-LanM. See legend for **Table S12** for full details.

		**La**	**Ce**	**Pr**	**Nd**	**Sm**	**Eu**	**Gd**	**Tb**	**Dy**
	D	**1.18(4)**	**2.14(8)**	**2.38(9)**	**1.92(8)**	**1.22(8)**	**0.88(8)**	**0.41(4)**	**0.25(3)**	**0.14(2)**
**La**	**1.18(4)**	1	1.82(1)	2.02(11)	1.63(9)	1.04(8)	0.75(7)	0.35(4)	0.21(3)	0.12(2)
**Ce**	**2.14(8)**	0.55(3)	1	1.11(6)	0.90(5)	0.57(4)	0.41(4)	0.19(2)	0.12(2)	0.07(1)
**Pr**	**2.38(9)**	0.49(3)	0.90(5)	1	0.81(5)	0.52(4)	0.37(4)	0.17(2)	0.10(1)	0.06(1)
**Nd**	**1.92(8)**	0.61(3)	1.12(6)	1.24(7)	1	0.64(5)	0.46(4)	0.22(2)	0.13(2)	0.07(1)
**Sm**	**1.22(8)**	0.96(7)	1.75(13)	1.94(14)	1.56(12)	1	0.72(8)	0.34(4)	0.20(3)	0.12(2)
**Eu**	**0.88(8)**	1.34(13)	2.43(23)	2.70(26)	2.17(21)	1.39(15)	1	0.47(6)	0.28(5)	0.16(3)
**Gd**	**0.41(4)**	2.84(31)	5.16(56)	5.73(62)	4.62(51)	2.95(35)	2.12(28)	1	0.60(10)	0.34(7)
**Tb**	**0.25(3)**	4.71(65)	8.57(1.19)	9.52(1.32)	7.68(1.07)	4.91(72)	3.53(56)	1.66(28)	1	0.57(12)
**Dy**	**0.14(2)**	8.34(1.50)	15.16(2.74)	16.84(3.04)	13.58(2.46)	8.68(1.62)	6.24(1.23)	2.94(60)	1.77(39)	1

Table S15. Amino acid and DNA sequences of constructs used in this study. The first residue of the cytosolically expressed *Hans*-LanM proteins (after cleavage of the N-terminal Met) is A24, the predicted signal peptide cleavage site according to SignalP 6.0; all residues are numbered based on the full-length sequence.

Construct	Protein or DNA sequence
Hans-LanM (full sequence, signal peptide underlined)	MKLSLKAGAA ITAFVFAASP VLAASGADAL KALNKDNDDS<br>LEIAEVIHAG ATTFTAINPD GDTTLESGET KGRLTEKDWA<br>RANKDGDQTL EMDEWLKILR TRFKRADANK DGKLTAAELD<br>SKAGQGVLVM IMK
Hans-LanM (lacking signal peptide, as expressed in this study)	MASGADAL KALNKDNDDS LEIAEVIHAG ATTFTAINPD<br>GDTTLESGET KGRLTEKDWA RANKDGDQTL EMDEWLKILR<br>TRFKRADANK DGKLTAAELD SKAGQGVLVM IMK
R100K-*Hans*-LanM (mutation underlined)	MASGADAL KALNKDNDDS LEIAEVIHAG ATTFTAINPD<br>GDTTLESGET KGRLTEKDWA RANKDGDQTL EMDEWLKILK<br>TRFKRADANK DGKLTAAELD SKAGQGVLVM IMK
Hans-LanM-Cys (for immobilization)	MASGADAL KALNKDNDDS LEIAEVIHAG ATTFTAINPD<br>GDTTLESGET KGRLTEKDWA RANKDGDQTL EMDEWLKILR<br>TRFKRADANK DGKLTAAELD SKAGQGVLVM IMKGSGC
R100K-*Hans*-LanM-Cys (for immobilization)	MASGADAL KALNKDNDDS LEIAEVIHAG ATTFTAINPD<br>GDTTLESGET KGRLTEKDWA RANKDGDQTL EMDEWLKILK<br>TRFKRADANK DGKLTAAELD SKAGQGVLVM IMKGSGC
Hans-LanM codon-optimized DNA sequence for this study (EF-hands underlined)	ATGGCAAGTGGCGCGGATGCTTTGAAGGCGCTTAACAAAGACAAT<br>GACGATTCGCTGGAAATTGCAGAGGTAATCCACGCAGGCGCAACT<br>ACGTTCACGGCAATCAACCCGGACGGAGACACAACTTTGGAGAGC<br>GGAGAGACGAAAGGACGCTTGACAGAAAAGGATTGGGCTAGAGCT<br>AATAAAGACGGGGACCAGACGTTGGAAATGGACGAATGGCTGAAG<br>ATCCTGCGTACTAGATTTAAAAGAGCCGATGCTAATAAGGATGGC<br>AAATTAACGGCTGCGGAGTTGGATTCCAAAGCGGGGCAAGGGGTA<br>TTGGTCATGATCATGAAATGA
R100K-*Hans*-LanM DNA sequence (mutation underlined)	ATGGCAAGTGGCGCGGATGCTTTGAAGGCGCTTAACAAAGACAAT<br>GACGATTCGCTGGAAATTGCAGAGGTAATCCACGCAGGCGCAACT<br>ACGTTCACGGCAATCAACCCGGACGGAGACACAACTTTGGAGAGC<br>GGAGAGACGAAAGGACGCTTGACAGAAAAGGATTGGGCTAGAGCT<br>AATAAAGACGGGGACCAGACGTTGGAAATGGACGAATGGCTGAAG<br>ATCCTGAAAACTAGATTTAAAAGAGCCGATGCTAATAAGGATGGC<br>AAATTAACGGCTGCGGAGTTGGATTCCAAAGCGGGGCAAGGGGTA<br>TTGGTCATGATCATGAAATGA
Hans-LanM-Cys DNA sequence (GSGC underlined)	ATGGCAAGTGGCGCGGATGCTTTGAAGGCGCTTAACAAAGACAAT<br>GACGATTCGCTGGAAATTGCAGAGGTAATCCACGCAGGCGCAACT<br>ACGTTCACGGCAATCAACCCGGACGGAGACACAACTTTGGAGAGC<br>GGAGAGACGAAAGGACGCTTGACAGAAAAGGATTGGGCTAGAGCT<br>AATAAAGACGGGGACCAGACGTTGGAAATGGACGAATGGCTGAAG<br>ATCCTGCGTACTAGATTTAAAAGAGCCGATGCTAATAAGGATGGC<br>AAATTAACGGCTGCGGAGTTGGATTCCAAAGCGGGGCAAGGGGTA<br>TTGGTCATGATCATGAAAGGCAGCGGCTGCTGA
R100K-*Hans*-LanM-Cys DNA sequence	ATGGCAAGTGGCGCGGATGCTTTGAAGGCGCTTAACAAAGACAAT<br>GACGATTCGCTGGAAATTGCAGAGGTAATCCACGCAGGCGCAACT<br>ACGTTCACGGCAATCAACCCGGACGGAGACACAACTTTGGAGAGC<br>GGAGAGACGAAAGGACGCTTGACAGAAAAGGATTGGGCTAGAGCT<br>AATAAAGACGGGGACCAGACGTTGGAAATGGACGAATGGCTGAAG<br>ATCCTGAAAACTAGATTTAAAAGAGCCGATGCTAATAAGGATGGC<br>AAATTAACGGCTGCGGAGTTGGATTCCAAAGCGGGGCAAGGGGTA<br>TTGGTCATGATCATGAAAGGCAGCGGCTGCTGA

Table S16. Metal ion concentrations in the synthetic feed solution for on-column Nd/Dy separation in Fig. 4d and Extended Data Figure 6. Concentrations were determined by ICP-MS analysis.

	Mean concentration (μM)	Standard deviation	Coefficient of variation (%)
Dy	18.5	0.9	4.4
Nd	344.2	4.4	5.0

SUPPLEMENTARY REFERENCES

1 Mattocks, J. A., Tirsch, J. L. & Cotruvo, J. A., Jr. Determination of affinities of lanthanide-binding proteins using chelator-buffered titrations. *Methods Enzymol.* **651**, 23-61 (2021).

2 Cotruvo, J. A., Jr., Featherston, E. R., Mattocks, J. A., Ho, J. V. & Laremore, T. N. Lanmodulin: A highly selective lanthanide-binding protein from a lanthanide-utilizing bacterium. *J. Am. Chem. Soc.* **140**, 15056-15061 (2018).

3 Mattocks, J. A., Ho, J. V. & Cotruvo, J. A., Jr. A selective, protein-based fluorescent sensor with picomolar affinity for rare earth elements. *J. Am. Chem. Soc.* **141**, 2857-2861 (2019).

4 Mattocks, J. A., Cotruvo, J. A., Jr. & Deblonde, G. J. P. Engineering lanmodulin's selectivity for actinides over lanthanides by controlling solvent coordination and second-sphere interactions. *Chem. Sci.* **13**, 6054-6066 (2022).

5 Dong, Z. *et al.* Bridging hydrometallurgy and biochemistry: A protein-based process for recovery and separation of rare earth elements. *ACS Cent. Sci.* **7**, 1798-1808 (2021).

6 Featherston, E. R., Issertell, E. J. & Cotruvo, J. A. Probing lanmodulin's lanthanide recognition via sensitized luminescence yields a platform for quantification of terbium in acid mine drainage. *J. Am. Chem. Soc.* **143**, 14287-14299 (2021).

7 Halling, D. B., Liebeskind, B. J., Hall, A. W. & Aldrich, R. W. Conserved properties of individual Ca^{2+}-binding sites in calmodulin. *Proc. Natl. Acad. Sci. USA* **113**, E1216-E1225 (2016).

8 Svergun, D. I., Barberato, C. & Koch, M. H. J. CRYSOL - a program to evaluate X-ray solution scattering of biological macromolecules from atomic coordinates. *J. Appl. Cryst.* **28**, 768-773 (1995).

9 Grant, T. D. Ab initio electron density determination directly from solution scattering data. *Nat. Methods* **15**, 191-193 (2018).

10 Schrodinger, LLC. The PyMOL Molecular Graphics System, Version 1.8. (2015).

11 Petoukhov, M. V. & Svergun, D. I. Applications of small-angle X-ray scattering to biomacromolecular solutions. *Int. J. Biochem. Cell Biol.* **45**, 429-437 (2013).

12 Chattopadhyaya, R., Meador, W. E., Means, A. R. & Quiocho, F. A. Calmodulin structure refined at 1.7 Å resolution. *J. Mol. Biol.* **228**, 1177-1192 (1992).

13 Deng, Y. W., Ro, S. Y. & Rosenzweig, A. C. Structure and function of the lanthanide-dependent methanol dehydrogenase XoxF from the methanotroph *Methylomicrobium buryatense* 5GB1C. *J. Biol. Inorg. Chem.* **23**, 1037-1047 (2018).

14 Supkowski, R. M. & Horrocks, W. D. On the determination of the number of water molecules, *q*, coordinated to europium(III) ions in solution from luminescence decay lifetimes. *Inorg. Chim. Acta* **340**, 44-48 (2002).

15 Horrocks, W. D. J. Luminescence spectroscopy. *Methods Enzymol.* **226**, 495-538 (1993).

16 Beeby, A. *et al.* Non-radiative deactivation of the excited states of europium, terbium and ytterbium complexes by proximate energy-matched OH, NH and CH oscillators: an improved luminescence method for establishing solution hydration states. *J. Chem. Soc. Perkin Trans. 2*, 493-504 (1999).

17 Whelan, S. & Goldman, N. A general empirical model of protein evolution derived from multiple protein families using a maximum-likelihood approach. *Mol. Biol. Evol.* **18**, 691-699 (2001).

18 Cook, E. C., Featherston, E. R., Showalter, S. A. & Cotruvo, J. A., Jr. Structural basis for rare earth element recognition by *Methylobacterium extorquens* lanmodulin. *Biochemistry* **58**, 120-125 (2019).

19 Shannon, R. D. Revised effective ionic radii and systematic studies of interatomic distances in halides and chalcogenides. *Acta Cryst. A* **32**, 751-767 (1976).

20 Konarev, P. V., Volkov, V. V., Sokolova, A. V., Koch, M. H. J. & Svergun, D. I. PRIMUS - a Windows-PC based system for small-angle scattering data analysis. *J. Appl. Cryst.* **36**, 1277-1282 (2003).

21 Franke, D. *et al.* ATSAS 2.8: a comprehensive data analysis suite for small-angle scattering from macromolecular solutions. *J. Appl. Cryst.* **50**, 1212-1225 (2017).
