# Supplementary material for: Enhanced rare-earth separation with a metal-sensitive lanmodulin dimer
Source: Nature. 2023 May 31;618(7963):87–93. doi: 10.1038/s41586-023-05945-5 (PMC10232371; doi:10.1038/s41586-023-05945-5)
Supplement: Supplementary file 2 — Reporting Summary [file 41586_2023_5945_MOESM2_ESM.pdf]

nature portfolio

Corresponding author(s): Cotruvo, Boal, Park

Last updated by author(s): Feb 28, 2023

Reporting Summary

Nature Portfolio wishes to improve the reproducibility of the work that we publish. This form provides structure for consistency and transparency in reporting. For further information on Nature Portfolio policies, see our Editorial Policies and the Editorial Policy Checklist.

Statistics

For all statistical analyses, confirm that the following items are present in the figure legend, table legend, main text, or Methods section.

n/a	Confirmed	
☐	☒	The exact sample size (n) for each experimental group/condition, given as a discrete number and unit of measurement
☐	☒	A statement on whether measurements were taken from distinct samples or whether the same sample was measured repeatedly
☐	☒	The statistical test(s) used AND whether they are one- or two-sided<br>*Only common tests should be described solely by name; describe more complex techniques in the Methods section.*
☒	☐	A description of all covariates tested
☐	☒	A description of any assumptions or corrections, such as tests of normality and adjustment for multiple comparisons
☐	☒	A full description of the statistical parameters including central tendency (e.g. means) or other basic estimates (e.g. regression coefficient) AND variation (e.g. standard deviation) or associated estimates of uncertainty (e.g. confidence intervals)
☐	☒	For null hypothesis testing, the test statistic (e.g. F, t, r) with confidence intervals, effect sizes, degrees of freedom and P value noted<br>*Give P values as exact values whenever suitable.*
☒	☐	For Bayesian analysis, information on the choice of priors and Markov chain Monte Carlo settings
☒	☐	For hierarchical and complex designs, identification of the appropriate level for tests and full reporting of outcomes
☒	☐	Estimates of effect sizes (e.g. Cohen's d, Pearson's r), indicating how they were calculated

Our web collection on statistics for biologists contains articles on many of the points above.

Software and code

Policy information about availability of computer code

Data collection	Fluorescence data were acquired using BioTek Gen5 3.09 software. ITC data were collected using NanoAnalyze TM software. CD data were obtained using JASCO Spectra Manager TM. SAXS data were collected at the Penn State X-Ray Crystallography Facility using the instrumentation described in the Methods.
Data analysis	ITC data was analyzed by NanoAnalyze TM software. OriginLab was used for fitting data and statistical analysis. For structure determination, HKL2000 was used to determine the space group and process the data, and Phenix was used to solve the structures. Full details of all data analysis are described in the Supplementary Information.

For manuscripts utilizing custom algorithms or software that are central to the research but not yet described in published literature, software must be made available to editors and reviewers. We strongly encourage code deposition in a community repository (e.g. GitHub). See the Nature Portfolio guidelines for submitting code & software for further information.

Data

Policy information about availability of data

All manuscripts must include a data availability statement. This statement should provide the following information, where applicable:

- Accession codes, unique identifiers, or web links for publicly available datasets
- A description of any restrictions on data availability
- For clinical datasets or third party data, please ensure that the statement adheres to our policy

All data are available in the main text or the supplementary information. Coordinates have been deposited in the Protein Data Bank with accession codes: 8DQ2 (LaIII-Hans-LanM), 8FNR (DyIII-Hans-LanM), 8FNS (NdIII-Mex-LanM).

Human research participants

Policy information about studies involving human research participants and Sex and Gender in Research.

Reporting on sex and gender	n/a
Population characteristics	n/a
Recruitment	n/a
Ethics oversight	n/a

Note that full information on the approval of the study protocol must also be provided in the manuscript.

Field-specific reporting

Please select the one below that is the best fit for your research. If you are not sure, read the appropriate sections before making your selection.

☒ Life sciences ☐ Behavioural & social sciences ☐ Ecological, evolutionary & environmental sciences

For a reference copy of the document with all sections, see nature.com/documents/nr-reporting-summary-flat.pdf

Life sciences study design

All studies must disclose on these points even when the disclosure is negative.

Sample size	CD spectroscopy, ITC, and fluorescence experiments were performed three times, with few exceptions. The R100K-malonate fluorescence competition experiment was performed once for each condition, and the stoichiometric CD titration of Hans-LanM was performed twice, as noted in the relevant figure and table legends.
Data exclusions	no data were excluded
Replication	All experiments were conducted using technical replicates (independent experiments using the same stock of purified protein), with the number of experiments indicated in each figure or table legend.
Randomization	n/a
Blinding	n/a

Reporting for specific materials, systems and methods

We require information from authors about some types of materials, experimental systems and methods used in many studies. Here, indicate whether each material, system or method listed is relevant to your study. If you are not sure if a list item applies to your research, read the appropriate section before selecting a response.

Materials & experimental systems

n/a	Involved in the study
☒	☐ Antibodies
☒	☐ Eukaryotic cell lines
☒	☐ Palaeontology and archaeology
☒	☐ Animals and other organisms
☒	☐ Clinical data
☒	☐ Dual use research of concern

Methods

n/a	Involved in the study
☒	☐ ChIP-seq
☒	☐ Flow cytometry
☒	☐ MRI-based neuroimaging
